# Transcriptional changes in dendritic cells underlying allergen specific induced tolerance in a mouse model

**DOI:** 10.1038/s41598-022-06186-8

**Published:** 2022-02-18

**Authors:** Rafael Nuñez, Maria Jose Rodriguez, Francisca Palomares, Francisca Gomez, Fernando M. Jabato, Jose Cordoba-Caballero, Pedro Seoane, Jorge Losada, Javier Rojo, Maria Jose Torres, James Richard Perkins, Cristobalina Mayorga

**Affiliations:** 1 Allergy Research Group, Research Laboratory, Allergy Unit, Hospital Regional Universitario de Málaga-IBIMA, Instituto de Investigación Biomédica de Málaga-IBIMA, 29009 Málaga, Spain; 2 Allergy Clinical Unit, Hospital Regional Universitario de Málaga, Málaga, Spain; 3 Department of Molecular Biology and Biochemistry, University of Malaga, Malaga, Spain; 4 CIBER de Enfermedades Raras, Instituto de Salud Carlos III, Madrid, Spain; 5 Laboratory of Carbohydrates, Instituto de Investigaciones Químicas (IIQ), CSIC-Universidad de Sevilla, Sevilla, Spain; 6 Nanostructures for Diagnosing and Treatment of Allergic Diseases Laboratory, Centro Andaluz de Nanomedicina y Biotecnología-BIONAND, Málaga, Spain; 7 Medicine Department, Universidad de Málaga-UMA, Málaga, Spain

**Keywords:** Antigen processing and presentation, Applied immunology, Immunotherapy, Vaccines, Prognostic markers

## Abstract

To investigate food allergy-tolerance mechanisms induced through allergen-specific immunotherapy we used RNA-Sequencing to measure gene expression in lymph-node-derived dendritic cells from Pru p 3-anaphylactic mice after immunotherapy with glycodendropeptides at 2 nM and 5 nM, leading to permanent tolerance and short-term desensitization, respectively. Gene expression was also measured in mice receiving no immunotherapy (anaphylaxis); and in which anaphylaxis could never occur (antigen-only). Compared to anaphylaxis, the antigen-only group showed the greatest number of expression-changes (411), followed by tolerant (186) and desensitized (119). Only 29 genes changed in all groups, including *Il12b*, *Cebpb* and *Ifngr1*. The desensitized group showed enrichment for genes related to chronic inflammatory response, secretory granule, and regulation of interleukin-12 production; the tolerant group showed genes related to cytokine receptor activity and glucocorticoid receptor binding, suggesting distinct pathways for similar outcomes. We identified genes and processes potentially involved in the restoration of long-term tolerance via allergen-specific immunotherapy, representing potential prognostic biomarkers.

## Introduction

Food allergy (FA) is a growing problem, affecting up to 10% of the population in some Western countries^1^. Non-specific lipid transfer proteins (nsLTPs) like Pru p 3, the major peach allergen, are important plant-derived food allergens that can lead to severe reactions and present a wide range of cross-reactivity with other foods and pollens inducing a complex clinical pattern known as LTP syndrome^2^. This syndrome can be produced by multiple taxonomically related and unrelated allergenic sources, increasing the complexity of clinical management^3^. Reactions can be severe, and the high number of plant-based foods involved leads to dietary restrictions, greatly affecting quality of life. Currently, the only effective FA treatment is allergen-specific immunotherapy (AIT), which has been shown to improve LTP allergy^4^. However, its capacity to induce long-lasting tolerance is still an issue^5^ and, given its length and expense, there is much interest in finding potential prognostic biomarkers of treatment response.

In AIT for LTP with sublingual immunotherapy (SLIT), we demonstrated the shift of the sensitization profile from a Th2 to Th1/T regulatory (Treg) pattern with increased allergen-specific IgG4 (sIgG4) and IL-10^+^Treg cell levels and decreased allergen-specific IgE (sIgE) and effector Th2/Th9 cells^6^.

Moreover, in a mouse model, SLIT with glycodendropeptides (GDPs) bound to Pru p 3 peptides with mannose dendrons as adjuvant^7^ has been shown to protect from anaphylaxis upon allergen exposure, leading to a reduced Th2 response and increased Th1/Treg cells^7^. Interestingly, we observed that different doses of SLIT (5 nM or 2 nM) can lead respectively to either temporary desensitization (animal only tolerates peach for a short time after finishing treatment), or long-lasting tolerance (protection sustained for a longer period)^7^. This model provides us with a unique opportunity to evaluate the underlying mechanisms and identify biomarkers that discriminate between tolerance and desensitization by measuring changes in gene expression induced in the key cells for the modulation of the immune response, dendritic cells (DCs). We previously demonstrated that DCs showed important changes in cell-surface marker expression following stimulation by Pru p 3 in allergic patients compared to tolerant controls^8^. Multiple studies have demonstrated that these cells are key players in immunomodulation during AIT^9^ promoting tolerance, being involved in allergen transport to submandibular lymph nodes, inducing Tregs and reducing allergy symptoms^10^. Our group have demonstrated that the beneficial effects induced by SLIT with Pru p 3 can be orchestrated by DCs, by decreasing costimulatory molecules and migration markers and promoting PD-L1 expression, which is involved in the regulatory response^6,11^.

Moreover, a previous study of DC gene expression during Pru p 3-induced anaphylaxis^12^ showed changes for genes related to signal detection (including *Cd14*), effecting changes (Fos, Fosl2), Th2 responses, mast cell activation, vesicular trafficking and protein secretion, as well as cell recruitment and cytokines, further suggesting various roles for DCs in the allergic response. Interestingly, complement genes related to tolerant responses were downregulated^12^. However, few studies have looked at global changes in gene expression during AIT. Recent work looked at the effects of AIT on expression in nasal epithelia alongside associated effects on the microbiome^13^. To our knowledge, no such studies have evaluated the effect of AIT on DCs in terms of gene expression.

Here we use RNA-Sequencing (RNA-Seq) to examine transcriptional changes that occur in lymph node DCs during immunotherapy. This cell-type was chosen due to its potential role in the induction of allergen-induced anaphylaxis described above^12^ and in orchestrating the protective effects of AIT. The latter is shown by the aforementioned study from our group showing how targeting DCs in different ways during AIT leads to marked differences in response^7^. This encompasses differences at the immunological level, as shown by differences in levels of Treg cells and regulatory cytokines from both DCs and CD4^+^ T cells, and in terms of outcome for the mouse: the way DCs are targeted can influence whether tolerance is induced or not. The use of a high-throughput approach such as RNA-seq to profile DCs in this context allows us to further explore the involvement of regulatory cytokines in tolerance induction, as well as discovering novel genes and pathways. It will also allow us to further interpret previous results from our group focusing on changes in monocyte-derived dendritic cells in patients following immunotherapy, which involved a reduction of maturation markers, and their role in Th1 and Treg processes^6^.

For this, we used an animal model of food antigen induced-anaphylaxis^14^ developed by our group. We use two different concentrations of a SLIT compound that induce different protective responses: one that induces long-term protection (2 nM), and one that induces short-term (5 nM)^7^. RNA-seq was used to measure gene expression, due to its sensitivity to detect relatively small changes in expression^15^ and shed light on the dose-induced processes and pathways that produce different protective outcomes.

## Results

**Figure 1:**
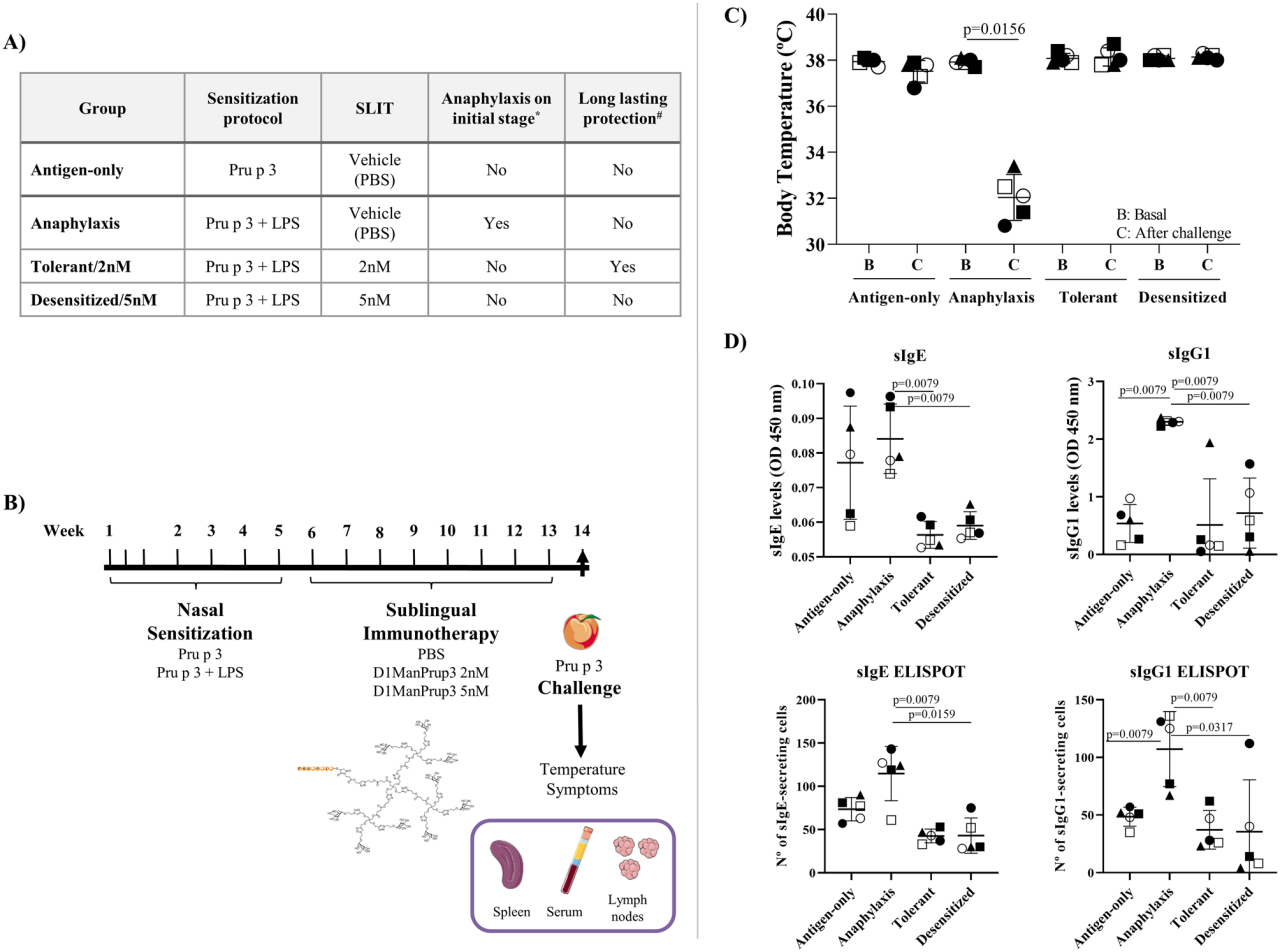
Experimental overview and physiological/immunological changes in the different groups. (**A**) Details of the sensitization and sublingual immunotherapy treatment for the different groups. *Challenge performed 1 week (1w) after the end of SLIT; #Were the challenge to be performed 5 weeks after the end of SLIT, as shown previously. (**B**) Overview of the experimental design in which mice were sensitized with Pru p 3 (Antigen-only) or Pru p 3 + LPS (additional three groups), then underwent sublingual immunotherapy, before being challenged one week after finishing treatment. (**C**) In vivo evaluation of anaphylaxis after SLIT with D_1_ManPrup3. Dots show decreases in body temperature 30–40 min after challenge with Pru p 3, 1w after ending the SLIT.. The individuals used for the subsequent RNA analysis are marked in black. (**D**) In vitro evaluation after SLIT with D_1_ManPrup3. Bars represent the mean serum level of Pru p 3-sIgE and -sIgG1 by ELISA in different groups (upper part) and the number of Pru p 3-specific IgE and IgG1 secreting cells by ELISpot assay for each group (lower part). Dots represent individual cases. Significant differences when *P* < 0.05 compared to Anaphylaxis group. The individuals used for the subsequent RNA analysis are marked in black.

All groups of mice were treated with different doses of SLIT with D_1_ManPrup3, or PBS, according to the experimental design (Fig. 1A). All mice were then challenged at week 14, one week after their final treatment in order to confirm the immunological response. Only anaphylactic mice (not treated with D_1_ManPrup3) underwent a drop in temperature (Δ6°C, *P* = 0.0156) and developed severe clinical symptoms (Fig. 1C). Tolerance at 5 weeks was observed in Tolerant/2 nM animals, but not in Desensitized/5 nM animals (data not shown), in line with previous findings^7^. Pru p 3-sIgE and -sIgG1 levels and secreting cells were significantly lower in the Tolerant/2 nM and Desensitized/5 nM mice compared to Anaphylaxis, although values were low in all groups. However, we observed Pru p 3-sIgE in the Antigen-only group to be of similar levels to Anaphylaxis groups. These results are in line with previous studies^14,16^, and suggest that not only sIgE but also sIgG1 may be required for the development of anaphylaxis symptoms. The mice that developed anaphylactic symptoms (Fig. 1C) also showed a significant change in sIgE levels after the immunization period, with more severe symptoms in those mice with higher levels of sIgE to Pru p 3 (Fig. 1D).

### RNA sequencing and read alignment

Of the 20 mice (5 per group), 17 passed quality control criteria, having an average RIN of 8.67 and ~ 33,000,000 reads per sample, of which 96% aligned to the genome, and 84% overlapped with annotated genes.

**Figure 2:**
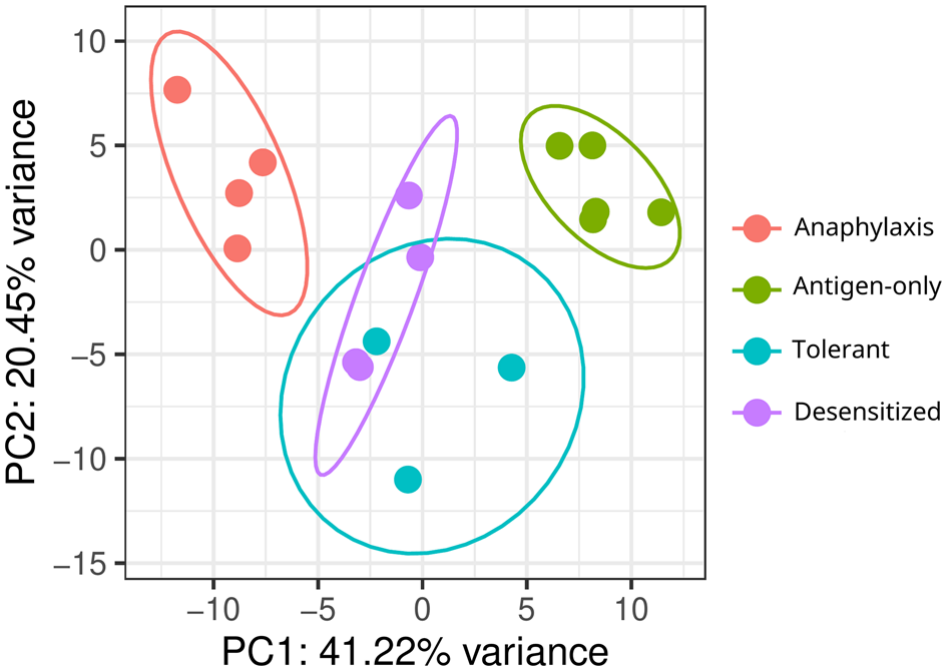
Principal Components Analysis (PCA) representation. Principal component analysis was obtained using the 1000 most variable genes. PC1 and PC2 are depicted.

To assess group separation and discard possible outlier samples, a principal components analysis was conducted (Fig. 2). The different groups show clear separation, except for the two SLIT receiving groups (Tolerant/2 nm and Desensitized/5 nm). Interestingly, the SLIT receiving groups are found together between the Anaphylaxis and Antigen-only group along the first principal component (PC1), which represents almost half of the variance, suggesting some commonality in terms of their gene expression profiles. However, they show some separation along PC2, suggesting that differences exist.

### A lower dose of SLIT induces tolerant responses and more significant and larger expression changes

**Figure 3:**
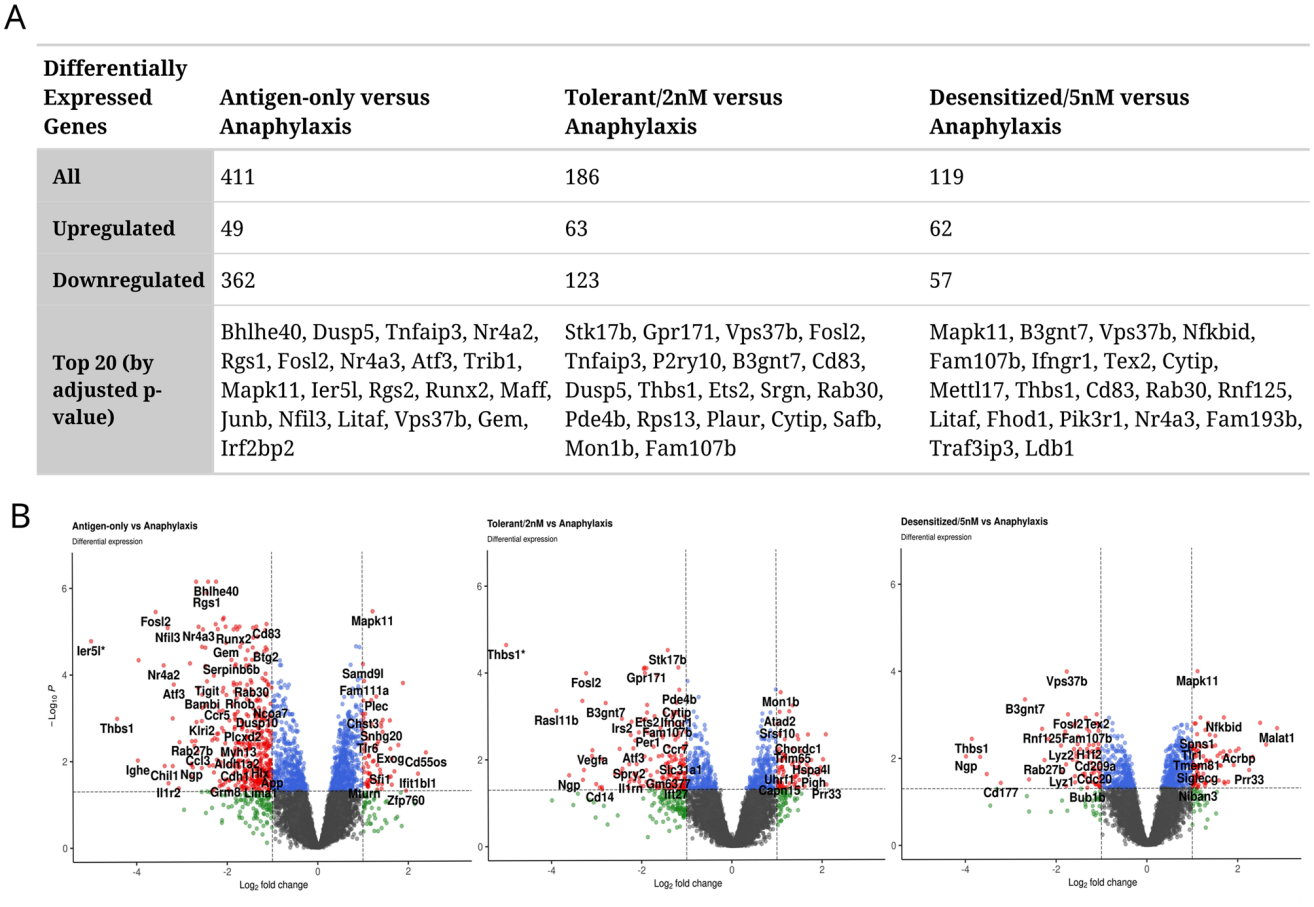
Global changes in gene expression between groups. (**A**) Table summarizing the number of differentially expressed genes (DEGs) in the different groups, Antigen-only, Tolerant/2 nm and Desensitized/5 nm, compared to the Anaphylaxis animal group (*P*-value < 0.05, abs(log2 fold change) > 1). (**B**) Volcano plots for each comparison, adjusted *P*-value vs. fold change for the different groups vs. Anaphylaxis. *DEGs with absolute Log2FC > 6.

Given that the principal aim of the study was to look at the effects of SLIT on animals that would react with anaphylaxis to Pru p 3, gene expression for the Tolerant/2 nm, Desensitized/5 nm and Antigen-only groups was compared with Anaphylaxis. The greatest number of DEGs was found for the Antigen-only group (411), as expected given that this group was never capable of developing anaphylaxis upon challenge. This was followed by Tolerant/2 nM (186), with the fewest changes being found for Desensitized/5 nM (119) (Fig. 3A). Full details of the DEGs are shown in Supporting Table 1.

**Table 1:** Top 20 enriched GO terms for each comparison.

	Antigen-only	Tolerant/2 nM	Desensitized/5 nM
	Term (adjusted *P*-value | mean log_2_Fold-change)	Term (adjusted *P*-value | mean log_2_Fold-change)	Term (adjusted *P*-value | mean log_2_Fold-change)
Immune general	Negative regulation of immune system process (3.75e-13 |− 1.60)	Negative regulation of immune system process (0.002 |− 1.88) Regulation of hemopoiesis (0.003 |− 1.04)	Negative regulation of immune system process (0.002 |− 0.59) **Defense response to Gram-positive bacterium** (0.008 |− 1.42) **Chronic inflammatory response** (0.01 |− 1.12) Regulation of hemopoiesis (0.003 |− 0.25)
Immune cells processes	**Regulation of leukocyte differentiation** (2.61e − 13 |− 1.48) **Lymphocyte differentiation** (3.74e − 13 |− 1.29) Leukocyte cell–cell adhesion (2.07e − 12 |− 1.48) Regulation of cell–cell adhesion (3.70e − 12 |− 1.58) Regulation of T cell activation (9.62e − 12 |− 1.43) Positive regulation of cell activation (4.26e − 11 |− 1.52) **Negative regulation of leukocyte activation** (2.42e − 10 |− 1.46) **Leukocyte proliferation** (4.64e − 09 |− 1.34) **Regulation of leukocyte proliferation** (6.29e − 09 |− 1.31) Alpha–beta T cell activation (6.87e − 09 |− 1.40) **Myeloid leukocyte activation** (6.93e − 09 |− 1.41) Regulation of lymphocyte proliferation (6.93e − 09 |− 1.43) **Cell chemotaxis** (6.93e − 09 |− 1.65) Lymphocyte proliferation (1.59e − 08 |− 1.42) **Immune receptor activity** (2.02e − 08 |− 1.50)	**Glucocorticoid receptor binding** (3.70e − 05 |− 1.67) Regulation of cell–cell adhesion (0.002 |− 0.89) Regulation of T cell activation (0.002 |− 0.34) Leukocyte cell –cell adhesion (0.002 |− 0.41) Alpha–beta T cell activation (0.005 |− 0.72)	Regulation of lymphocyte proliferation (0.0008 |− 0.46) **Regulation of mononuclear cell proliferation** (0.0008 |− 0.46) **Positive regulation of leukocyte activation** (0.003 |− 0.77) Positive regulation of cell activation (0.003 |− 0.77) Lymphocyte proliferation (0.003 |− 0.46) **Mononuclear cell proliferation** (0.003 |− 0.46) Leukocyte cell–cell adhesion (0.003 |− 0.70) **Regulation of leukocyte cell–cell adhesion** (0.009 |− 0.39) Regulation of T cell activation (0.01 |− 0.65) Alpha–beta T cell activation (0.01 |− 0.80)
Cytokines related	Positive regulation of cytokine production (9.44e − 14 |− 1.53) Negative regulation of cytokine production (2.75e − 09 |− 1.56) Cytokine binding (3.40e − 08 |− 1.52)	Cytokine binding (2.58e − 06 |− 1.49) **Cytokine receptor activity** (1.04e − 05 |− 0.51) **C–C chemokine receptor activity** (0.002 |− 0.75) **G protein-coupled chemoattractant receptor activity** (0.002 |− 0.75) **Chemokine receptor activity** (0.002 |− 0.75) Positive regulation of cytokine production (0.002 |− 1.57) Negative regulation of cytokine production (0.003 |− 2.37)	**Secretory granule** (0.0002 |− 1.49) **Regulation of interleukin-12 production** (0.01 |− 1.45) **Interleukin-12 production** (0.01 |− 1.45)
Cellular metabolism & signaling		**Negative regulation of phosphorylation** (0.002 |− 1.08) **Response to unfolded protein** (0.002 |− 0.66) **Negative regulation of transferase activity** (0.002 |− 1.14) **Negative regulation of MAPK cascade** (0.002 |− 0.98) **Positive regulation of cellular catabolic process** (0.002 |− 0.99) **DNA-binding transcription factor binding** (0.004 |− 1.34)	**Negative regulation of cystein**e − **type endopeptidase activity** (0.008 |− 1.80) **Negative regulation of hydrolase activity** (0.009 |− 1.67) **Positive regulation of tyrosine phosphorylation of STAT protein** (0.01 | 0.74)
Other	**Regulation of vasculature development** (7.60e − 09 |− 1.83)		

Adjusted *P*-value and mean log2FC for each team are shown. Terms unique for each comparison top 20 enriched terms are in bold.

Genes with higher expression in the comparison group are deemed over-expressed (positive log2FC), genes with higher expression in the Anaphylaxis group under-expressed (negative log2FC). More genes were detected as under-expressed in the Antigen-only (362 versus 49), and Tolerant/2 nM comparisons (123 versus 63) and over-expressed in Desensitized/5 nM (62 versus 57) (Fig. 3A,B). DEGs dispersion from the center of the Volcano plots in Fig. 3B also confirms more significant and larger changes for the Antigen-only comparison, followed by Tolerant/2 nM and Desensitized/5 nM.

Additionally, to investigate the DC tolerogenic state for each group, a group of manually curated genes related to tolerance were selected, based on a review of the relevant literature^17–21^ . To illustrate the expression differences of these activation markers we plotted a Log2Fold-Change heatmap (Supporting Fig. 2) and counts per million boxplots (Supporting Fig. 3) for these genes. Most of these genes were not detected as differentially expressed, moreover some were not even expressed, as shown as grey tiles in the heatmap. However, Il12b, Tnf, Xcr1, Ltf, Il1b, Ifngr1, Ifng, Tlr1, Tlr6, Cxcr4, Cebpb, Cd83, Cd274, Cd14, Ccr7, Ccr2 and Ccl22 were differentially expressed in at least one of the groups.

### SLIT dosage affects the expression of genes related to anaphylaxis suppression

Table 1 summarizes the top 20 enriched GO terms for each comparison. Terms uniquely enriched for each comparison are highlighted. Only 4 GO terms of the top 20 are shared across all comparisons, these terms were the negative regulation of immune system process, leukocyte cell–cell adhesion, regulation of T cell activation and alpha beta T cell activation. Although the exact GO terms differed between comparisons, they all found enrichment related to immune system processes characterized by DC-related processes.

The Tolerant/2 nM comparison found terms related to glucocorticoid receptor binding, cytokine receptor activity and negative regulation of MAPK cascade. Conversely, the Desensitized/5 nM comparison found terms such as secretory granule, regulation of mononuclear cells proliferation and chronic inflammatory response. Supporting Tables 2, 3 and 4 contain full details of gene ontology enrichment. Although different terms were found between the two treated groups, they can potentially lead to the suppression of anaphylaxis through distinct mechanisms, as described in the next section.

### Long lasting tolerance involves different mechanisms and signaling pathways compared to short term desensitization

**Figure 4:**
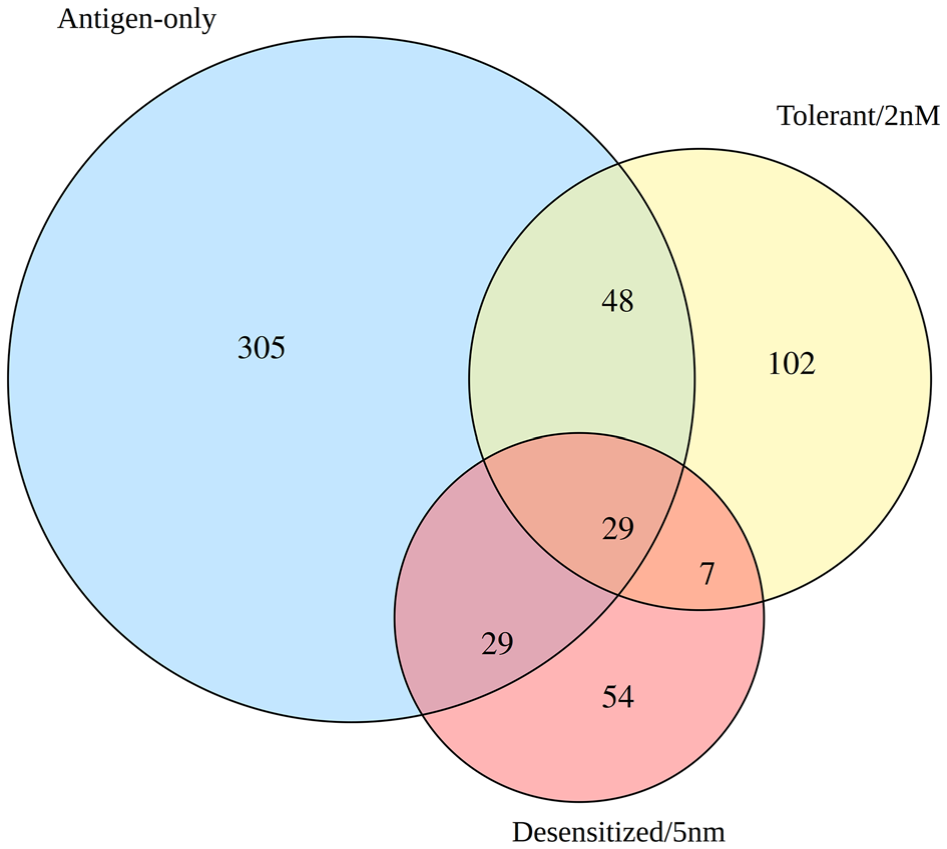
Area proportional Venn Diagram comparing DEGs lists obtained in all three comparisons.

Overlap between DEGs for each group is shown in Fig. 4. Only 29 DEGs showed significant differential expression in all three groups, unsurprising given the differences in terms of symptoms and immunological changes. The overlap between Desensitized/5 nM and Tolerant/2 nM only contained 7 DEGs that were not present in Antigen-only, somewhat unexpected given they both received the same treatment, GDP D_1_ManPrup3, albeit in different doses. Lists of DEGs for the different intersections are given in Supporting Table 5.

**Figure 5:**
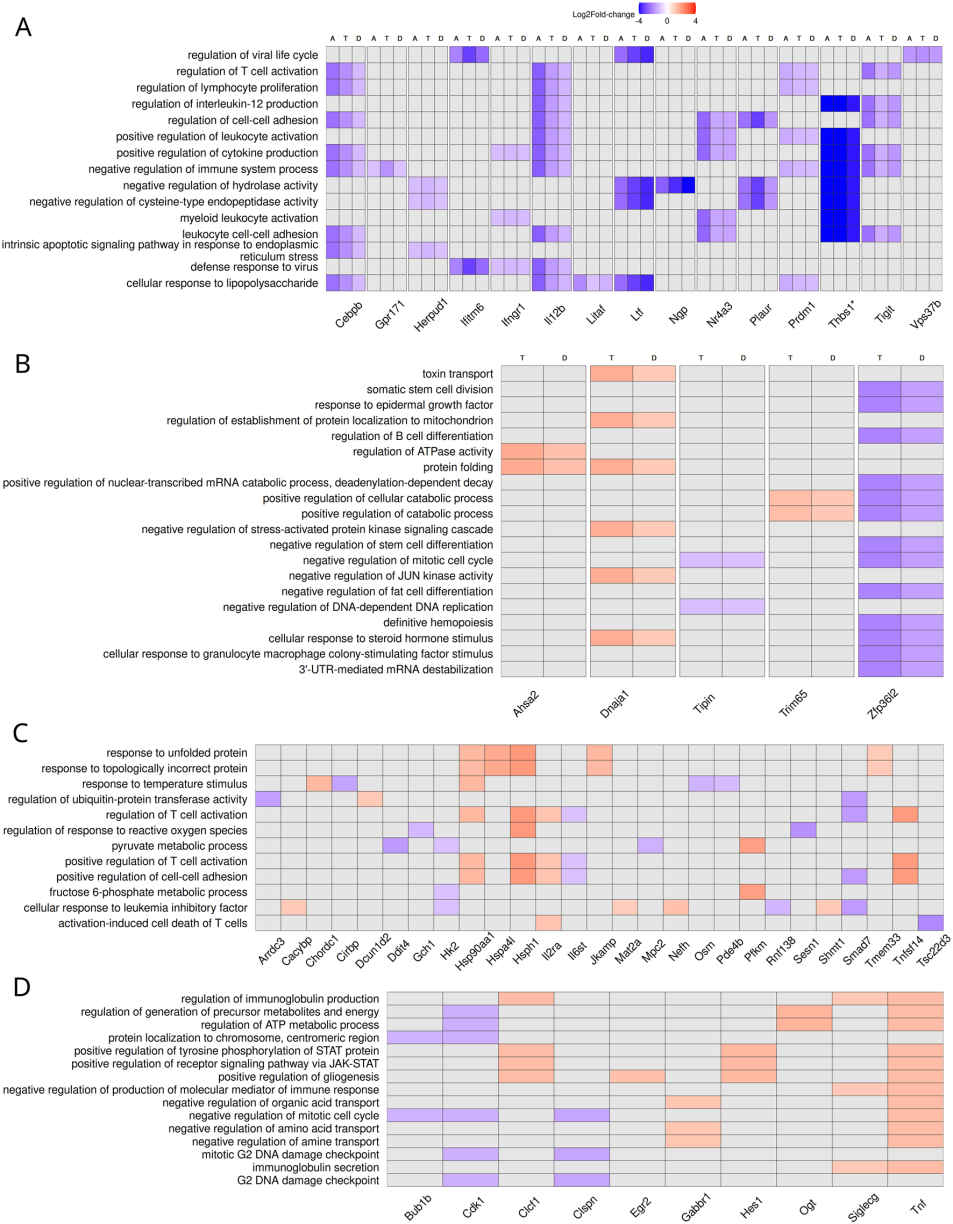
Venn Regions enriched Biological Processes Gene Ontology terms – DEGs heatmap. Heatmaps (Log2FC): (**A**) Common DEGs (A: Antigen-only, T: Tolerant/2 nM, D: Desensitized/5 nM), (**B**) Common DEGs for SLIT receiving groups (T: Tolerant/2 nM, D: Desensitized/5 nM), (**C**) Tolerant/2 nM exclusive DEGs, (**D**) Desensitized/5 nM exclusive DEGs. * DEGs with absolute Log2FC > 4.

The 29 common DEGs were used for functional enrichment analysis. Fourteen under-expressed genes showed functional enrichment for the gene ontology terms shown in Fig. 5A. All enriched terms are immune related, with links to DC functions. The differences in logFC between groups for *Il12b*, *Cebpb* or *Ltf*, could underlie different DC behaviors.

Similar analysis was performed for all Venn diagram regions (Fig. 5 and Supporting Figs. 4–6). The DEGs shared by the Desensitized/5 nM and Tolerant/2 nM comparisons are involved in more general cellular processes and apparently unrelated terms such as fat cell differentiation and toxin transport. These may represent pleiotropic processes that affect the DC transcriptional and cellular context after immunotherapy, affecting their participation in tolerance. The genes changing in Tolerant/2 nM exclusively (Fig. 5C) are related to stress responses, including regulation of response to ROS, metabolic processes such as pyruvate metabolic process, and immune regulation terms such as activation-induced cell death of T cells. Desensitized/5 nM exclusive DEGs (Fig. 5D) also show enrichment for metabolic processes, such as negative regulation of amino acid transport, and immune-related process such as the regulation of immunoglobulin production, implying alterations of DC metabolic state in addition to the expected changes in the immunological machinery, in both experimental groups receiving SLIT. These findings suggest that the different SLIT doses lead to tolerance through different mechanisms and signaling pathways with some shared components.

## Discussion

This study offers multiple insights into how AIT exerts its immunological effects. By using AIT models that led to either long-lasting tolerance or transient desensitization, we were able to investigate tolerance response mechanisms^7^. Through the use of a murine model, we could also access lymphatic tissue and thus focus on DCs, key players in the immune response orchestration^6,10^. In terms of immunological changes, there was variation in the levels of both sIgE and sIgG1 between groups, potentially due to the signals occurring during the presentation of food antigens to T lymphocytes by DCs, inducing differentiation to IL-4 and IL-13 and proliferation of Th2 cells^22^, which together with the CD40-CD40L interaction with B cells will induce isotype-switching^23^.

The patterns of gene expression detected are in keeping with the changes seen at the long-term phenotypic level. The Antigen-only animals, which although sensitized never developed symptoms against Pru p 3, showed the largest number of DEGs when compared with the Anaphylaxis group. Of both treated groups, the Tolerant/2 nM group showed fewer changes in expression, whilst the Desensitized/5 nM group showed the fewest. This could be interpreted as the effects of the AIT wearing off, although it might also be related to the induction of different pathways by different doses of AIT, this is reinforced by the relatively low number of common DEGs detected between Desensitized/5 nM and Tolerant/2 nM, despite both groups receiving the same SLIT molecule and showing similar immunological responses one week after finishing treatment. However, there is a core group of 29 genes that change in all three groups compared to Anaphylaxis. Several down-regulated genes in this group are related to Th1 activity: *Il12b* and *Ifngr1* downregulation increase IL-12 activity and therefore Th1 responses^24,25^. Ifgnr1 downregulation has also been linked to the absence of DC maturation and proinflammatory cytokines^26^, as have other downregulated genes such as *Thsb1*^27^, *Prdm1*^28^ and *Nr4a3*^29^. Moreover, two downregulated transcription factors, *Fosl2*^30^ and *Cebpb*^31^, belonging to the AP-1 and CEBP transcription factor family respectively, are related to proinflammatory pathways. This common differential expression shows absence of anaphylaxis after the challenge. Comparing functional enrichment of the DEGs detected in the Tolerant/2 nM and Desensitized/5 nM comparisons shows differences in DC pathway regulation and immune effector processes related to Pru p 3 tolerance at one week after SLIT treatment.

In the light of the above, Tolerant/2 nM detected DEGs may play a role in long-term protection. This set of DEGs includes under-expressed genes that reinforce DC tolerogenic behaviour, such as typical DC activation markers, *Ccr7*^32^ and *Cd83*^33^, including also *Cxcr4*^34^, *Cytip*^35^, *Il6st*^36^, *Rel*^37,38^, *Smad7*^39,40^, *Pde4b*^41^, *Cd14*^21^, *Zbtb10*^42^, *Sik1*^43^ and *Tnfaip3*^44,45^. Moreover, multiple over-expressed genes concur with DC tolerogenic behaviour: *Alg3*^46^, *Il2ra*^47^ and *Uhfr1*^48,49^. Interestingly, the Tolerant/2 nM over-expressed genes include chaperones and co-chaperones connected to antigen processing and presentation, and eventually to tolerance induction, such as *Hsph1*^19^, *Hsp90aa1*^50^ and *Stip1*^19^. *Dgcr8,* involved in miRNA production, was also over-expressed, potentially of interest given that these small RNAs have an important role in DC regulation^51^.

The DC tolerogenic skew is governed by mTOR signalling, as previously described in several studies^52,53^. Activation or inhibition can be involved, depending on the cellular context and TLR signals. MTOR activation has been suggested to induce tolerance in lymph node DCs^52,53^. Tolerant/2 nM differential gene expression presents a collection of genes that are tied to mTOR activation. An up-regulated gene, *Nuak1*, activates mTOR in response to calcium^54^, and is regulated by *Maf,* which is also over-expressed in Tolerant/2 nM. *Maf* is in turn regulated by mTOR to induce IL-10 production after TLR4 signalling^55^. Furthermore, the over-expressed transcription factor, *Cebpg*, activates mTOR signalling and promotes a tolerogenic state in DCs^31^. Four inhibitors of the mTOR pathway, *Ddit4*^56^, *Pik3ip1*^57^, *Mpc2*^58^ and *Sesn1*^59^, were under-expressed, endorsing increased mTOR activation in Tolerant/2 nM DCs in comparison to Anaphylaxis DCs. That said, other genes were found that may down-regulate mTOR signalling, such as over-expressed *Neil3* that activates the mTOR antagonist p53 pathway^60,61^, *Arl4d*^62^ and *Gpr68*^63^, that can negatively regulate PI3K/AKT/mTOR. Furthermore, four mTOR activators, *Slc38a7*, *Dyrk3*, *Irs2* and *Dkge*, were under-expressed.

Also related to mTOR signalling, another Tolerant/2 nM only DEG of interest was *Mat2a*, whose up-regulation is controlled by mTOR^64^; this gene encodes an enzyme with a key role in DNA methylation synthesis *S*-Adenosyl methionine (SAM). Increased abundance of SAM influences methylation and can attenuate LPS induced inflammation in macrophages^65^. Conversely, *Myc* is detected as under-expressed. Myc is an mTOR-regulated transcriptional regulator involved in DC-induced tolerance^66,67^. We also found over-expression of one of its direct targets, *Shmt1*, which is also over-expressed in dexamethasone-induced tolerogenic DCs (tolDCs)^67^.

Meanwhile, genes changing in Desensitized/5 nM only may lead to short term protection. In this group, most of the detected DEGs are over-expressed, including *Nfkbid*, an inhibitor of NF-κβ, which limits inflammation^68^ and *Tnf*, a mainly proinflammatory cytokine that induces DCs to activate IL-10 secretion by CD4 + T-cells^69^. *Tnf* is regulated, together with IL-6 and IL-1, by mTOR signalling^53^. Related to this finding, the mTOR-dependant up-regulated transcript, *Malat1*^70^, drives DCs towards a tolerogenic phenotype^71^, and is related to milder asthma symptoms^72^. In contrast, *Egr2,* also induced by mTOR and up-regulated^73^, may underlie the tendency to develop a Th2 allergic response in later weeks in these animals^74–76^. *Egr2* is closely linked to methylation in immune cells, for example it initiates DNA demethylation in IL-4/GM-CSF-driven monocyte differentiation into DCs^77^. A number of Desensitized/5 nM only over-expressed genes also activate the mTOR signalling pathway. *Zfp692* activates the PI3K/AKT pathway^78^. PI3K/AKT signalling is key to maintaining tolDCs glycolytic regulation^79^, especially in Vitamin D3 induced tolDCs^80^. *Hes1* can activate mTOR via PTENT repression^81^. *Mapk11* also activates mTOR^82^ and participates in tolDCs generation^83^. On the other hand, *Anxa1* down-regulation reduces mTOR/S6 signalling^84^.

Other Desensitized/5 nM only DEGs are downregulated but connected to transitory desensitization. For example, *Camp* encodes an antimicrobial peptide that reduces TLR activation, linked to allergic contact response^85^, and *209a* encodes DC-sign, which is needed for the *Malat1* induced DC tolerogenic phenotype^71^. However, *Ahr*, is also downregulated, deficiency of which could promote a Th2 response^86^.

The two SLIT receiving groups only shared 7 DEGs that were not found in the Antigen-only comparison. Of these, an up-regulated co-chaperone stands out: *Ahsa2* encodes a Hsp90 binding and activating co-chaperone^87^. Hsp90 inhibition in DCs leads to decreased maturation, MHCII and costimulatory surface markers, and prevents DCs from presenting antigen to T-cells and IL-12 secretion^50^.

The effects of allergen dosage on DC response have been previously described at the expression and methylation level: increasing doses of OVA leads to increased numbers of changes in both^88^. However, these results have no direct translation to our study, as we are looking at modulatory and tolerogenic expression changes compared to reactive anaphylactic mice. In this study we have shown how different SLIT doses (5 nM or 2 nM), which induce short-term (desensitization) or long-term protection (tolerance) against Pru p 3 in a mouse-model also result in distinct transcriptional panoramas, compared to anaphylactic animals. In both profiles of differential gene expression, several DEGs point to DC tolerogenic behaviour via mTOR activation after TLR4 stimulation, however the vast majority of the leading DEGs involved were specific to each comparison. A multi-faceted tolerance-related pathway such as mTOR has multiple input and output signals, with a plethora of regulating agents involved, so it is not surprising to find it involved in tolerance promotion. Additional studies could examine this further by performing RNA-seq at different time points, both during and after finishing AIT, to be able to capture all transient expression changes and to investigate how mTOR-dependant tolerance is achieved and then retained or lost, based on short- and long-term transcriptional profile divergence. Although DCs are theoretically the only CD11c expressing cells in lymph nodes^89–91^, further studies using additional markers could be performed to investigate more specific subtypes. Moreover, since differential methylation may be involved in Pru p 3 tolerance, this should be studied further. Here, we found two over-expressed genes with contrasting effects on methylation: For the Desensitized/5 nM comparison, we found DEG *Egr2,* involved in demethylation; conversely, for the Tolerant/2 nM comparison, we found DEG *Mat2a*, which promotes methylation through SAM synthesis. Future studies should focus on long term DNA methylation changes emerging after SLIT, since using expression changes solely, at protein or RNA level, could reduce the time window in which transient changes can be detected. Finally, given that both Desensitized/5 nM and Tolerant/2 nM groups showed similar immunological data upon finishing treatment, these dose-related differentially expressed genes also warrant further investigation in terms of their use as prognostic biomarkers for AIT efficacy, although it must be pointed out that this study was performed using lymph node derived material.

The study has important implication for: (i) Understanding the mechanisms underlying the changes brought about by AIT in general. (ii) Understanding the implication of lymph nodes dendritic cells specifically – something that would not be feasible in human studies, at least on a high-throughput level. (iii) Determining what differentiates successful AIT from unsuccessful at a mechanistic level (iv) Identifying potential biomarkers that might be used prognostically to determine AIT success.

## Methods and materials

### GDP synthesis

SLIT was administered as GDP, comprising a dendrimer backbone, coupled with mannose and a Pru p 3 peptide, including T and B epitopes^92^. Synthesis and characterization were carried out as described^7^.

### Food-antigen induced anaphylaxis model and SLIT experimental design

The aim of the experiment was to profile transcriptional changes occurring during SLIT in mice that would undergo anaphylaxis if exposed to the peach antigen Pru p 3. For this, gene expression in mice that had been given SLIT was compared to gene expression in mice that did not receive SLIT. This comparison was performed for different doses of SLIT capable of inducing long lasting tolerance (Tolerant/2 nM group) and short-term desensitization (Desensitized/5 nM group), respectively^7^. Therefore, three groups of mice that would undergo anaphylaxis if exposed to the peach antigen Pru p 3 were required, two of which would then go on to receive SLIT. As such, three groups of Balb/c mice were sensitized intranasally to peach using Pru p 3 alongside low doses of LPS in order to generate a Th2 response and allergic reaction (Fig. 1B)^14,93^. For this purpose, mice were anaesthetized with inhaled sevoflurane and sensitized with 20 µg of natural Pru p 3 (Bial Laboratory, Zamudio, Spain) and 20 ng of LPS (InvivoGen, San Diego, CA) with a final volume of 12 µL.

In addition to these three groups, we also included an Antigen-only group, sensitized only to Pru p 3, without LPS. Mice in this group showed no anaphylactic symptoms after challenge with Pru p 3. Gene expression for the animals in this group was also compared to the mice that would undergo anaphylaxis if exposed to the peach antigen Pru p 3 and had not been given SLIT.

Once sensitized, Tolerant and Desensitized mice were treated with 2 or 5 nM of D_1_ManPrup3 respectively via sublingual administration (10 µL), once a week for eight weeks. The same schedule was followed but with 10 µL of PBS in mice from the Anaphylaxis and Antigen-only groups. Mice were anaesthetized to ensure the compound was maintained sublingually. One week after finishing SLIT/PBS, mice were challenged with one intraperitoneal dose of Pru p 3 (100 µg) and 30–40 min after the injection, temperature and behavioral symptoms were measured as described previously (Fig. 1B)^94^. Mice were then sacrificed, lymph nodes were collected aseptically and teased to prepare a cell suspension. DCs were purified by positive selection using CD11c MicroBeads Ultrapure (Miltenyi Biotec, Bergisch Gladbach, Germany). The CD11c + cell fraction was maintained in RLT Buffer at − 80 °C until use.

### Immunological changes

The methods to measure immunological changes were similar to those of our previous studies, in which we developed the anaphylactic and tolerant models^7,14^. Briefly, Pru p 3-specific serum antibody levels (sIgE and sIgG1) were measured at basal time (day 0) and after Pru p 3-challenge in mice sera by ELISA using 5 µg/mL of Pru p 3 for coating microtiter plates (Corning, Corning, NY). The Pru p 3-specific antibodies were revealed using biotinylated labeled rat anti-mouse IgE or IgG1, (BD Biosciences Pharmingen, San Diego, CA). The ELISA results were expressed as optical density. Statistical analysis was performed by analyzing unrelated samples using the Mann–Whitney U or Kruskal–Wallis tests. *P*-values lower than 0.05 were considered statistically significant.

The number of antibody-secreting cells was measured using ELISpot Multiscreen HTS plates (Millipore, Darmstadt, Germany) coated with Pru p 3 at 5 µg/mL. A total of 1.5 × 10^5^ splenocytes were seeded and incubated for 48 h at 37 °C and with 5% of CO_2_. After washing, 50 µL of alkaline phosphatase labeled anti-IgE and -IgG1 and as control -IgM (SouthernBiotech, Birmingham, AL) were added. The number of Ig secreting cells was determined by counting the formed spots using an ELISpot Bioreader(R) 6000 (BioSys, Karben, Germany).

### RNA extraction and sequencing

Total RNA was isolated from the CD11c + cell fraction and purified using the RNeasy Micro Kit following the manufacturer's instructions (Qiagen, Venlo, Netherlands). RNA quality was verified using an Agilent Bioanalzyer, with an RNA integrity number cut-off of 8.

Poly-A enriched sequences were reverse transcribed, fragmented, and amplified using the SMARTer Universal Low RNA Kit (Clontech, Mountain View, CA). Sequencing was performed using Hiseq 4000 (Illumina, San Diego, CA), 100 base pair reads (paired-end).

### Bioinformatics analysis

A bioinformatic analysis workflow (Supporting Fig. 1) was created for this purpose and was composed of the steps described below. Sequenced reads were cleaned of low-quality bases, sequencing adapters and low-quality reads based on substandard sequencing tiles. Later, sequenced reads were aligned to the mouse genome (GRCm39) using STAR software (STAR 2.7.8a)^95^ Gene expression was quantified using Ensembl annotation (103) and featureCounts from the Rsubread package^96^. Detection of differentially expressed genes (DEGs) was performed using EdgeR^97,98^, Limma^99^ and DESeq2^100^: a false discovery rate of 0.05 and an absolute base 2 logarithmic fold change (log2FC) of 1 in all three packages were used to determine significant DEGs. Given that the aim of the study was to investigate changes induced by AIT, the Anaphylaxis group was used as the control for all comparisons, as such positive log2FC represents higher expression in the Tolerant/2 nM, Desensitized/5 nM or Antigen-only groups; negative log2FC represent higher expression in the Anaphylaxis group.

The DEGs were used for functional enrichment analysis by detecting overrepresented gene groups amongst the DEGs compared to non-DEGs, using Gene Ontology (GO), (molecular function and biological process) annotation^101^, performed using clusterProfiler with default parameters^102^, and an adjusted *P*-value < 0.05. All software is publicly available.

DEGs detection and functional enrichment was performed using the freely available ExpHunterSuite software package (https://bioconductor.org/packages/ExpHunterSuite/)^103^. 

### Animal experiments

This study was carried out in accordance with the “*Guide for the Care and Use of Laboratory Animals*” as promulgated by the National Institute of Health and the protocols were approved by the “*Animal Experimentation Ethics Committee of BIONAND*”, Malaga, Spain (Ref 07/2017). All studies involving animals are reported in accordance with the *ARRIVE guidelines* for reporting experiments involving animals^104^.

## Supplementary Information

Supplementary Information 1.

Supplementary Information 2.

Supplementary Information 3.

Supplementary Information 4.

## Data Availability

The data that support the findings of this study are openly available in BioProject (PRJNA805201).
