# Supplementary material for: Transcriptional changes in dendritic cells underlying allergen specific induced tolerance in a mouse model
Source: Sci Rep. 2022 Feb 18;12:2797. doi: 10.1038/s41598-022-06186-8 (PMC8857182; doi:10.1038/s41598-022-06186-8)
Supplement: Supplementary file 1 — Supplementary Information 1. [file 41598_2022_6186_MOESM1_ESM.pdf]

Transcriptional changes in dendritic cells underlying allergen specific induced tolerance in a mouse model.

Rafael Nuñez, MSc[a,*], Maria Jose Rodriguez PhD[a,*], Francisca Palomares PhD[a], Francisca Gomez MD, PhD[b], Fernando M. Jabato[c], Jose Cordoba-Caballero[c], Pedro Seoane[c,d], Jorge Losada[e], Javier Rojo PhD[e], Maria Jose Torres MD, PhD [a,b,f,g], James Richard Perkins PhD [a,c,d#], Cristobalina Mayorga PhD [a,b,f#].

Supporting figure 1. Bioinformatic analysis workflow.

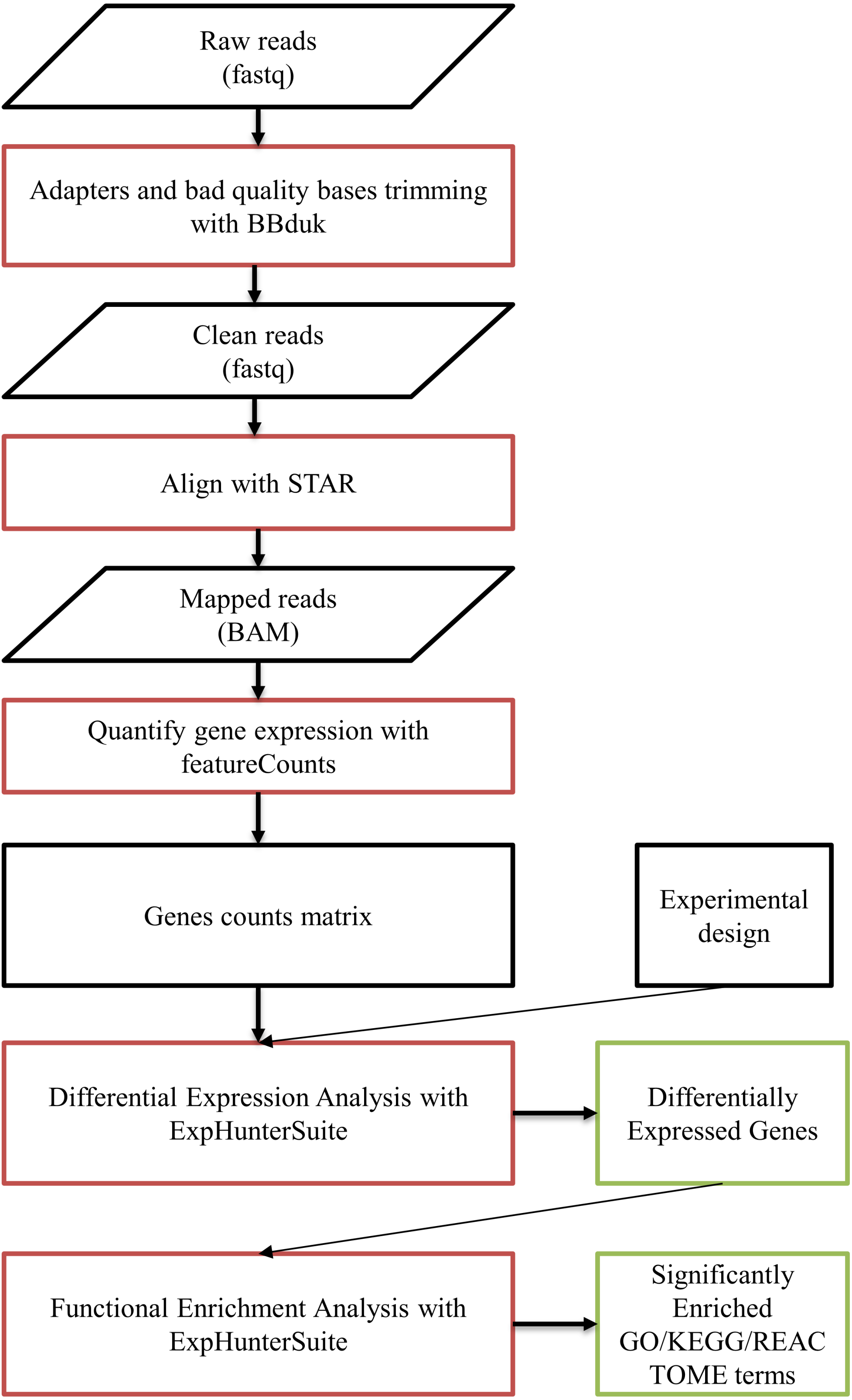

Raw reads
(fastq)
Adapters and bad quality bases trimming with BBduk
Clean reads
(fastq)
Align with STAR
Mapped reads
(BAM)
Quantify gene expression with featureCounts
Genes counts matrix
Experimental design
Differential Expression Analysis with ExpHunterSuite
Differentially Expressed Genes
Functional Enrichment Analysis with ExpHunterSuite
Significantly Enriched GO/KEGG/REACTOME terms

Supporting figure 2. Dendritic cells tolerogenic signature genes Heatmap. Genes for which no expression has been detected have been coloured white.

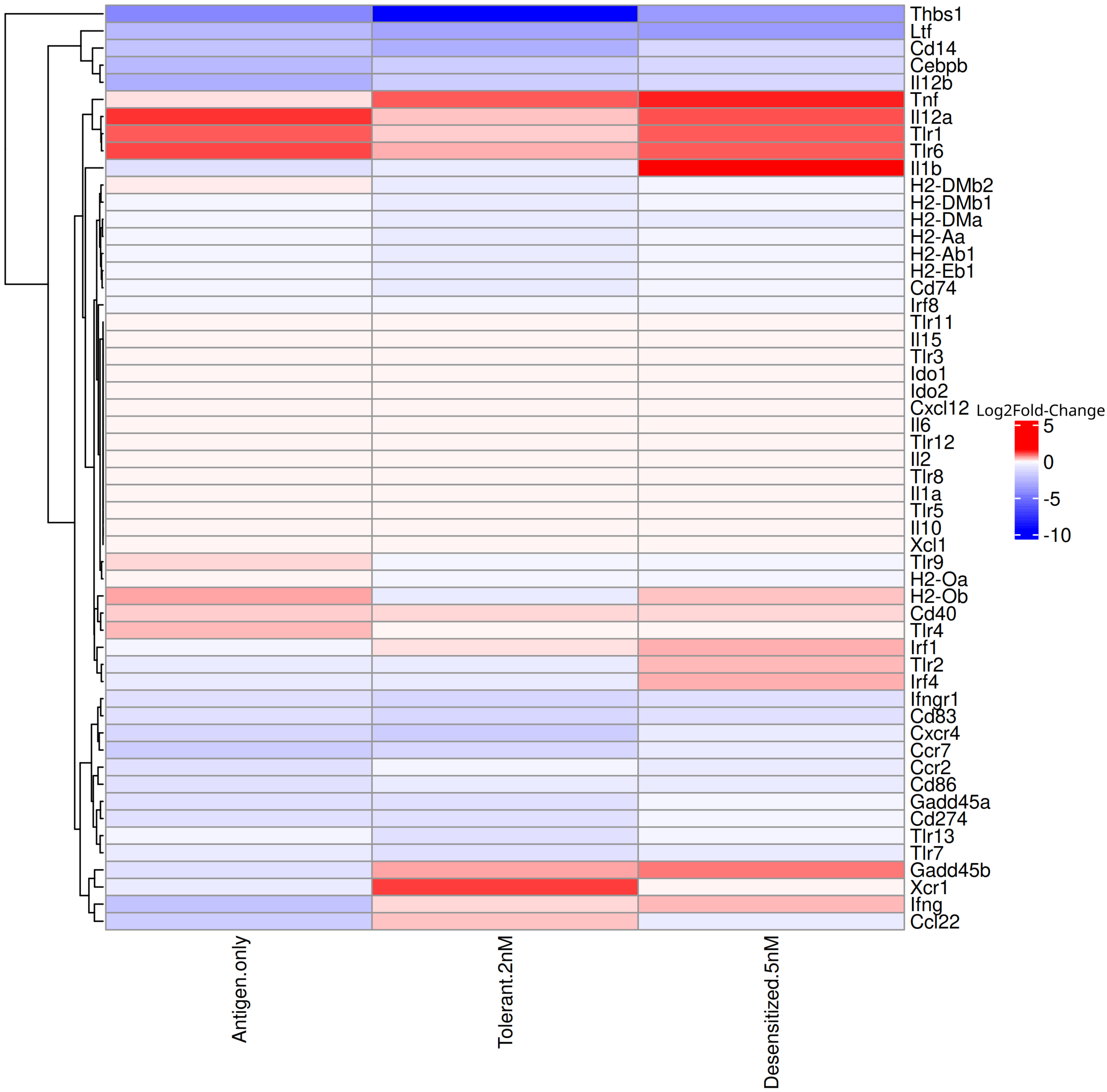
Thbs1
Ltf
Cd14
Cebpb
Il12b
Tnf
Il12a
Tlr1
Tlr6
Il1b
H2-DMb2
H2-DMb1
H2-DMa
H2-Aa
H2-Ab1
H2-Eb1
Cd74
Irf8
Tlr11
Il15
Tlr3
Ido1
Ido2
Cxcl12
Il6
Tlr12
Il2
Tlr8
Il1a
Tlr5
Il10
Xcl1
Tlr9
H2-Oa
H2-Ob
Cd40
Tlr4
Irf1
Tlr2
Irf4
Ifngr1
Cd83
Cxcr4
Ccr7
Ccr2
Cd86
Gadd45a
Cd274
Tlr13
Tlr7
Gadd45b
Xcr1
Ifng
Ccl22
Log2Fold-Change
5
0
-5
-10
Antigen.only
Tolerant.2nM
Desensitized.5nM

Supporting figure 3. Dendritic cells tolerogenic signature genes counts per million Boxplots. *Differentially expressed for this group against Anaphylaxis.

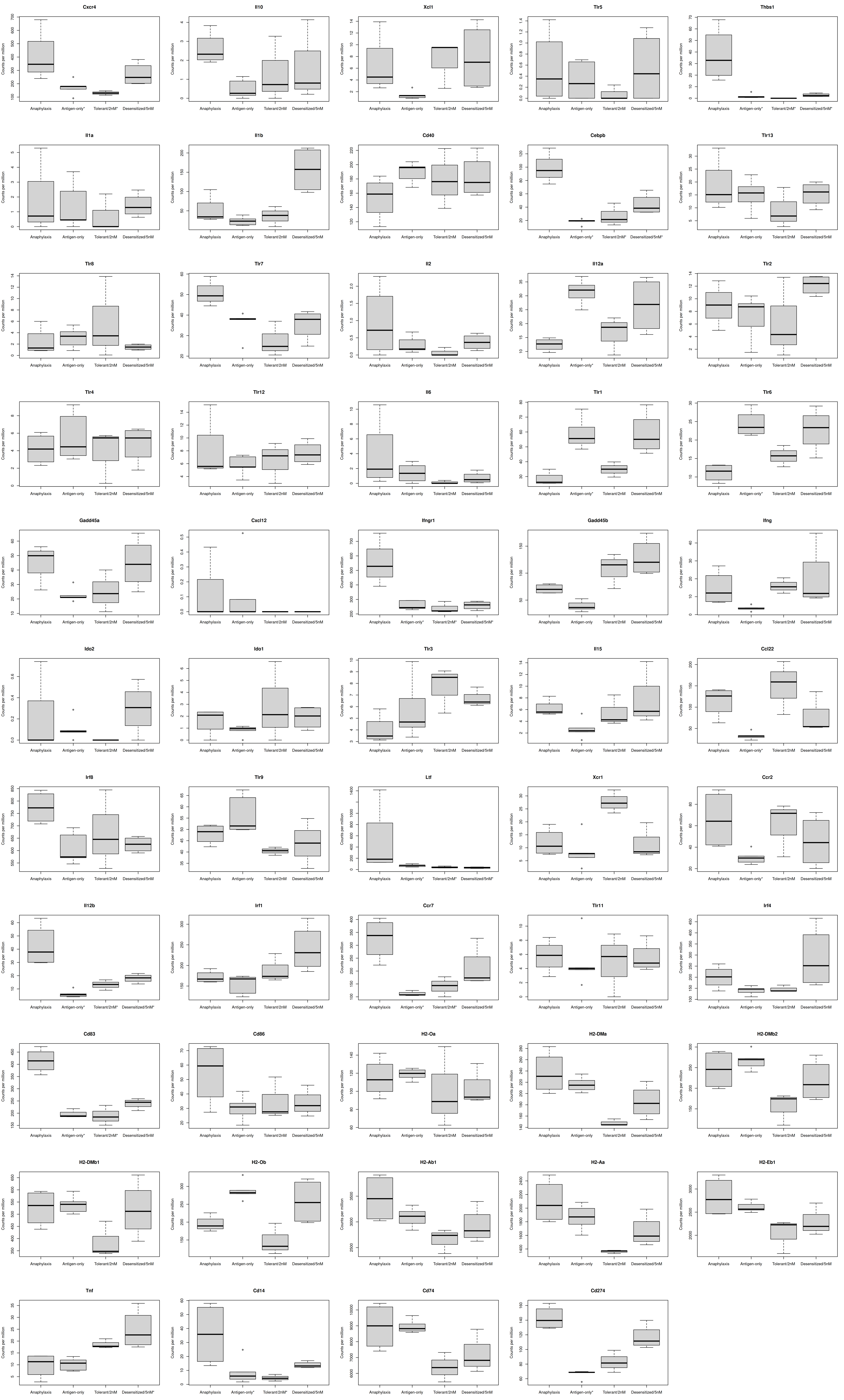

Cxcr4
Il10
Xcl1
Tlr5
Thbs1
Il1a
Il1b
Cd40
Cebpb
Tlr13
Tlr8
Tlr7
Il2
Il12a
Tlr2
Tlr4
Tlr12
Il6
Tlr1
Tlr6
Gadd45a
Cxcl12
Ifngr1
Gadd45b
Ifng
Ido2
Ido1
Tlr3
Il15
Ccl22
Irf8
Tlr9
Ltf
Xcr1
Ccr2
Il12b
Irf1
Ccr7
Tlr11
Irf4
Cd83
Cd86
H2-Oa
H2-DMa
H2-DMb2
H2-DMb1
H2-Ob
H2-Ab1
H2-Aa
H2-Eb1
Tnf
Cd14
Cd74
Cd274
Counts per million
Anaphylaxis
Antigen-only
Antigen-only*
Tolerant/2nM
Tolerant/2nM*
Desensitized/5nM
Desensitized/5nM*

Supporting figure 4. Heatmap (Log2Fold-Change) of Antigen-only exclusive DEGs.

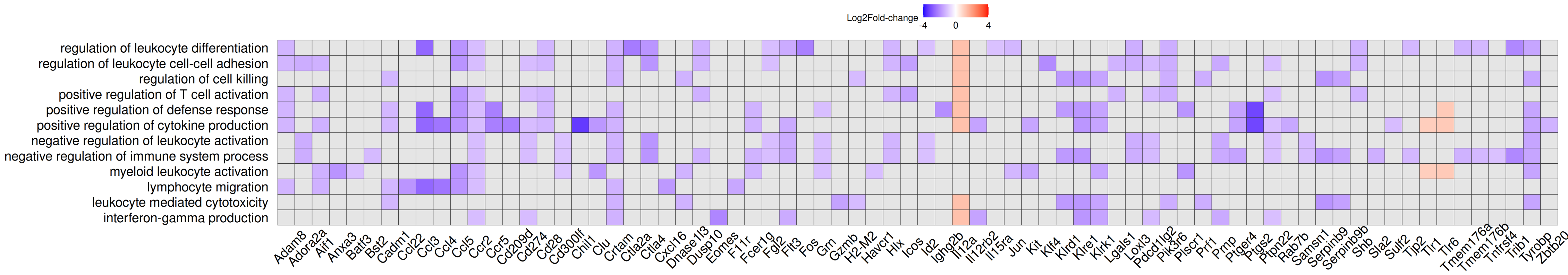
Log2Fold-change
-4
0
4
regulation of leukocyte differentiation
regulation of leukocyte cell-cell adhesion
regulation of cell killing
positive regulation of T cell activation
positive regulation of defense response
positive regulation of cytokine production
negative regulation of leukocyte activation
negative regulation of immune system process
myeloid leukocyte activation
lymphocyte migration
leukocyte mediated cytotoxicity
interferon-gamma production
Adam8
Adora2a
Aif1
Anxa3
Batf3
Bst2
Cadm1
Ccl22
Ccl3
Ccl4
Ccl5
Ccr2
Ccr5
Cd209d
Cd274
Cd28
Cd300lf
Chil1
Clu
Crtam
Ctla2a
Ctla4
Cxcl16
Dnase1l3
Dusp10
Eomes
F11r
Fcer1g
Fgl2
Flt3
Fos
Grn
Gzmb
H2-M2
Havcr1
Hlx
Icos
Id2
Ighg2b
Il12a
Il12rb2
Il15ra
Jun
Kit
Klf4
Klrd1
Klre1
Klrk1
Lgals1
Loxl3
Pdcd1lg2
Pik3r6
Plscr1
Prf1
Prnp
Ptger4
Ptgs2
Ptpn22
Rab7b
Samsn1
Serpinb9
Serpinb9b
Shb
Sla2
Sulf2
Tlp2
Tlr1
Tlr6
Tmem176a
Tmem176b
Tnfrsf4
Trib1
Tyrobp
Zbtb20

Supporting figure 5. Heatmap (Log2Fold-Change) of Antigen-only and Desensitized/5nM exclusive DEGs.

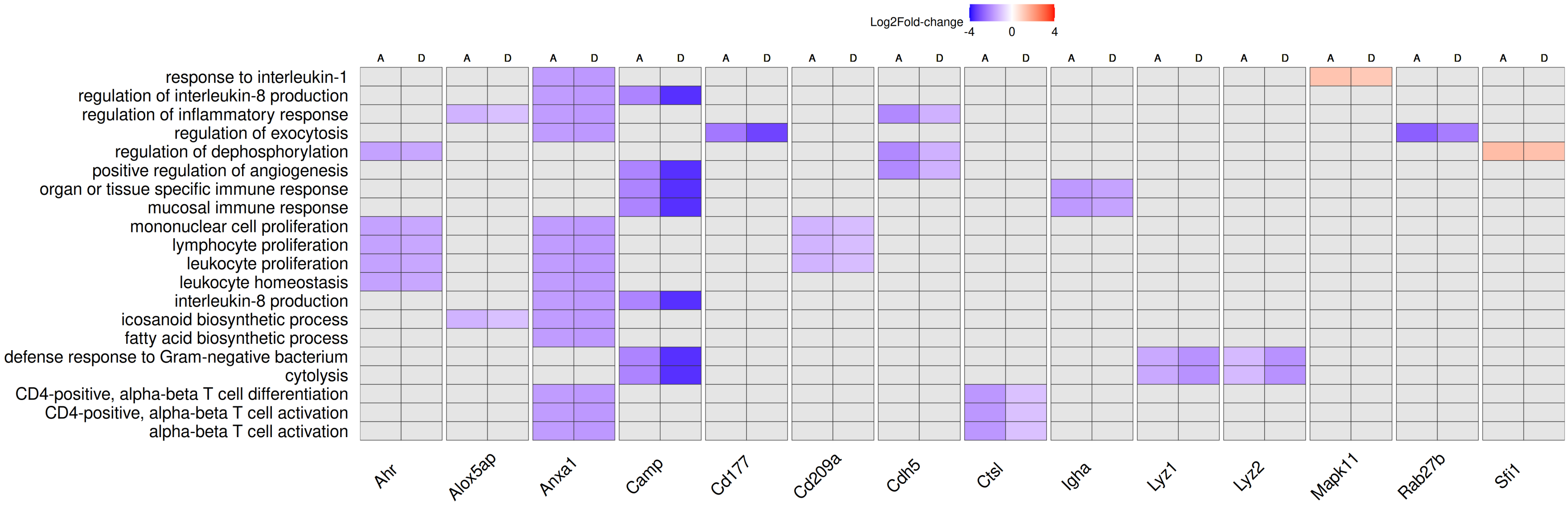
Log2Fold-change
-4
0
4
A
D
response to interleukin-1
regulation of interleukin-8 production
regulation of inflammatory response
regulation of exocytosis
regulation of dephosphorylation
positive regulation of angiogenesis
organ or tissue specific immune response
mucosal immune response
mononuclear cell proliferation
lymphocyte proliferation
leukocyte proliferation
leukocyte homeostasis
interleukin-8 production
icosanoid biosynthetic process
fatty acid biosynthetic process
defense response to Gram-negative bacterium
cytolysis
CD4-positive, alpha-beta T cell differentiation
CD4-positive, alpha-beta T cell activation
alpha-beta T cell activation
Ahr
Alox5ap
Anxa1
Camp
Cd177
Cd209a
Cdh5
Ctsl
Igha
Lyz1
Lyz2
Mapk11
Rab27b
Sfi1

Supporting figure 6. Heatmap (Log2Fold-Change) of Antigen-only and Tolerant/2nM exclusive DEGs.

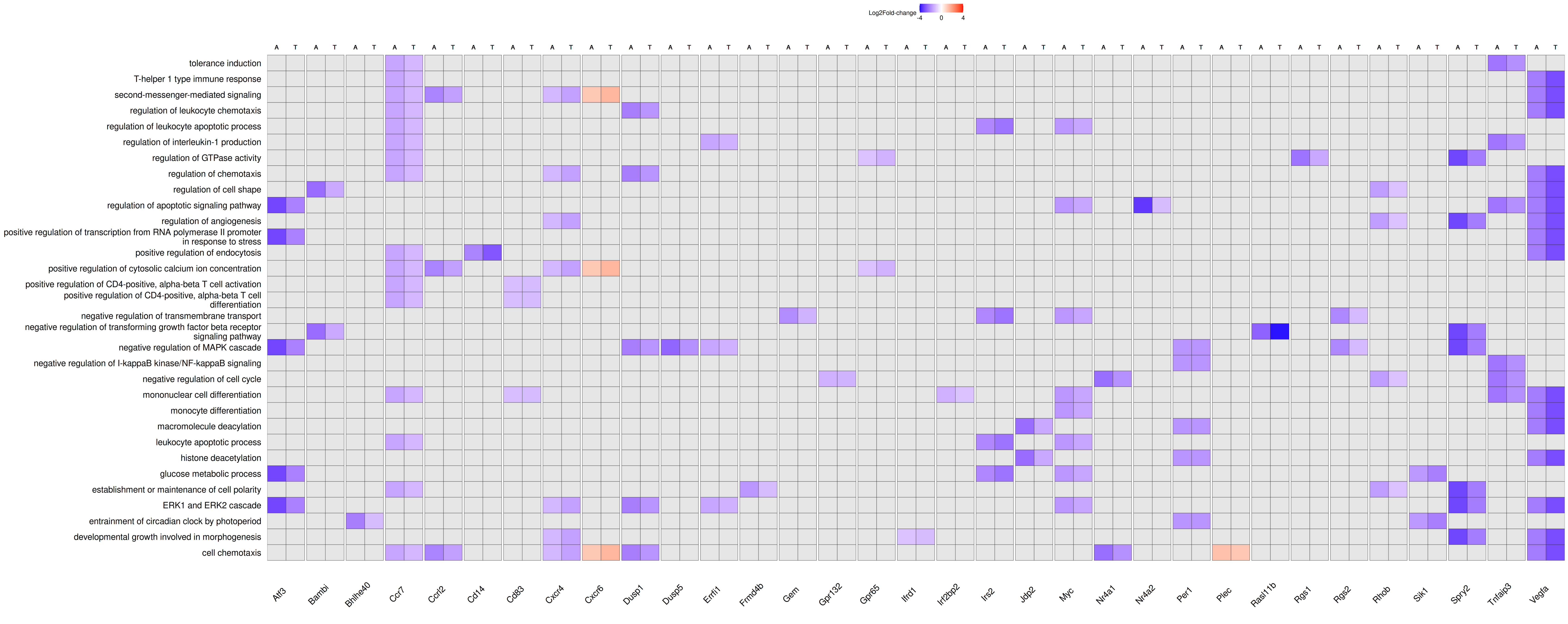
Log2Fold-change
-4
0
4
A
T
tolerance induction
T-helper 1 type immune response
second-messenger-mediated signaling
regulation of leukocyte chemotaxis
regulation of leukocyte apoptotic process
regulation of interleukin-1 production
regulation of GTPase activity
regulation of chemotaxis
regulation of cell shape
regulation of apoptotic signaling pathway
regulation of angiogenesis
positive regulation of transcription from RNA polymerase II promoter in response to stress
positive regulation of endocytosis
positive regulation of cytosolic calcium ion concentration
positive regulation of CD4-positive, alpha-beta T cell activation
positive regulation of CD4-positive, alpha-beta T cell differentiation
negative regulation of transmembrane transport
negative regulation of transforming growth factor beta receptor signaling pathway
negative regulation of MAPK cascade
negative regulation of I-kappaB kinase/NF-kappaB signaling
negative regulation of cell cycle
mononuclear cell differentiation
monocyte differentiation
macromolecule deacylation
leukocyte apoptotic process
histone deacetylation
glucose metabolic process
establishment or maintenance of cell polarity
ERK1 and ERK2 cascade
entrainment of circadian clock by photoperiod
developmental growth involved in morphogenesis
cell chemotaxis
Atf3
Bambi
Bhlhe40
Ccr7
Ccrl2
Cd14
Cd83
Cxcr4
Cxcr6
Dusp1
Dusp5
Errfi1
Frmd4b
Gem
Gpr132
Gpr65
Ifrd1
Irf2bp2
Irs2
Jdp2
Myc
Nr4a1
Nr4a2
Per1
Plec
Rasl11b
Rgs1
Rgs2
Rhob
Sik1
Spry2
Tnfaip3
Vegfa

Supporting Table 1. Details of all the genes showing differential expression in at least one of the groups compared to Anaphylaxis. Columns show FDR (adjusted p-value) and logFC for all three comparisons**.**

Supporting Table 1. Full details of genes differentially expressed

	Antigen-only.logFC_DESeq2	Antigen-only.FDR_DESeq2	Antigen-only.logFC_edgeR	Antigen-only.FDR_edgeR	Antigen-only.logFC_limma	Antigen-only.FDR_limma	Tolerant/2nM.logFC_DESeq2	Tolerant/2nM.FDR_DESeq2	Tolerant/2nM.logFC_edgeR	Tolerant/2nM.FDR_edgeR	Tolerant/2nM.logFC_limma	Tolerant/2nM.FDR_limma	Desensitized/5nm.logFC_DESeq2	Desensitized/5nm.FDR_DESeq2	Desensitized/5nm.logFC_edgeR	Desensitized/5nm.FDR_edgeR	Desensitized/5nm.logFC_limma	Desensitized/5nm.FDR_limma
**Bhlhe40**	-2.24565270440726	4.20352957742123e-35	-2.18531773211499	8.73936590007074e-07	-2.24304046340089	7.17760745911387e-07	-1.11873755591186	1.70755778325443e-06	-1.07586906651009	0.00292425536603383	-1.1714565097335	0.00156156460186529	-0.112660549812012	0.744071092068542	-0.0474121462053005	0.899576048031029	-0.132291170347307	0.646656631750067
**Dusp5**	-2.68957747507052	4.20352957742123e-35	-2.63135735599963	1.13383857936169e-06	-2.67335089636999	7.17760745911387e-07	-1.87417956435779	1.2016351339854e-12	-1.83356493469216	0.00032328444869653	-2.0341615170867	0.0001029855223723	-1.0109362650357	0.000233118890552145	-0.945014250756611	0.0126879599633874	-1.00954996294532	0.00608381514430173
**Tnfaip3**	-2.40537583046942	7.82794273609641e-31	-2.34500006272789	1.70883089898129e-06	-2.45156434551982	7.17760745911387e-07	-1.91815479466563	1.76369561790659e-14	-1.87445161541093	0.000277821032242069	-1.98454541491559	7.70744410519003e-05	-0.881882998952062	0.000980952674211763	-0.813637860562155	0.0244247001056658	-0.877979575377203	0.0109365466330463
**Nr4a2**	-3.41102325587139	1.42053184671476e-26	-3.3521289632568	1.70883089898129e-06	-3.40340431818612	6.08608813774125e-05	-1.09986217978262	0.0116614222335764	-1.06172784184242	0.0465345236070281	-1.25190475622768	0.0211384659135404	-0.140844154719807	0.815469412110489	-0.0740428975312783	0.905507924497013	-0.137575775140967	0.743512952559927
**Rgs1**	-2.42694546172807	2.42716677766124e-24	-2.37033407309601	9.3523009081234e-06	-2.49240894384866	1.29956415938385e-06	-1.46749609202273	1.29395301424318e-06	-1.43053474460152	0.00599515713964875	-1.55529893844422	0.000854472923213835	-0.601528696262726	0.0718430034156681	-0.537558392762329	0.185804092971603	-0.601849864443518	0.0657064228871846
**Fosl2**	-3.52662503075087	4.02434842684994e-24	-3.46113068221637	6.69432423087399e-06	-3.70166887210295	3.55879893543243e-06	-3.22484800849378	6.97358277601773e-15	-3.1754914631887	0.000286004448199555	-3.2576662940814	0.0001029855223723	-1.74836600035427	3.09025411521425e-05	-1.67993806072175	0.00827183148337801	-1.76417464712906	0.000995685637230599
**Nr4a3**	-2.65152152365261	2.42716677766124e-24	-2.58812333418422	6.00204728752055e-06	-2.60821221159355	7.86672124889252e-06	-1.55658969836531	2.48833991543322e-06	-1.50729319426719	0.00516032475995418	-1.51388616677501	0.00313649490323835	-1.56204880300271	5.45566621984122e-07	-1.49192412419049	0.00353127874376803	-1.64955863688568	0.00159338639086515
**Atf3**	-3.22202588397734	1.32313136952478e-24	-3.16355173180238	1.70883089898129e-06	-3.1152470890646	0.000168979337730591	-2.22587733622068	3.52951931270091e-09	-2.18203126538835	0.000731256977907823	-2.12106122724418	0.00559538701924121	0.272664974742182	0.593973368498163	0.335214171307457	0.47707779938178	0.359252508910451	0.324388758844143
**Trib1**	-2.07501003109168	3.4321518640779e-23	-2.01693770591329	2.65054952653441e-06	-2.08431224800506	4.9137546420781e-06	-0.998843620444039	0.000287648822187195	-0.957520628702062	0.0113211906817053	-1.05571134576998	0.00436202636186917	-0.16868915658078	0.645864623109139	-0.106322045158173	0.77873583669292	-0.161091667473252	0.544938927636791
**Mapk11**	1.20750366446113	1.60512541881645e-22	1.26531525671725	1.70883089898129e-06	1.21176068720342	3.55879893543243e-06	0.724401263282677	6.22046400467819e-06	0.768864211044936	0.00130869316300837	0.719922281007599	0.00352732904278951	1.10290385982604	5.39948614050537e-16	1.16739266628035	4.16050389159909e-05	1.10454768340664	0.000103247892277572
**Ier5l**	-5.176326490504	2.2491973985592e-22	-5.08694896492987	7.12983899511092e-06	-5.36582013974314	1.66534788525562e-05	-2.17125904759407	0.000621710566109268	-2.11906819042554	0.0168360412205771	-2.06670906157184	0.0215371072750291	-1.79230207854373	0.00266464345504752	-1.71861885786517	0.0221915233180815	-1.812715716125	0.0139941910045182
**Rgs2**	-2.09487268784056	8.2389757710836e-22	-2.03041530431303	7.12983899511092e-06	-2.10602173431724	5.32149854321288e-06	-1.1872389193681	2.90684879104777e-05	-1.14416037171806	0.0076555522182854	-1.22312819131291	0.00302029080670695	-0.321246986971988	0.352297750815397	-0.254738791285624	0.49266606787702	-0.319557895422466	0.294489627523661
**Runx2**	-1.87187113694815	4.62601677695926e-21	-1.81659726605839	5.15155059161785e-06	-1.83912286215207	9.0844466703e-06	-0.546704166894031	0.0620492268696561	-0.508102103437011	0.117220222153674	-0.580712375768954	0.0743925619132693	-0.882486914917444	0.000347893515770515	-0.819652469968344	0.0118233423700172	-0.86943823963145	0.00738654266376037
**Maff**	-3.92290633790232	1.54352518038199e-21	-3.84748560928869	8.47398130704746e-06	-4.05466973907584	4.60006800956485e-05	-2.20964822722625	1.36097432615113e-05	-2.16142257046731	0.00717976638997489	-2.32975437808057	0.00274888839778775	-1.25186576123671	0.0138671006700233	-1.18145188110718	0.0602867444227844	-1.24506111734696	0.0128755405056812
**Junb**	-1.74029769517073	2.16640414687456e-20	-1.67500763557104	9.3523009081234e-06	-1.71938325901293	7.86672124889252e-06	-0.819368641260387	0.00146461264541939	-0.773415602064804	0.0252520301853721	-0.819413268935678	0.0207728536138624	0.0175518928723405	0.968822464001963	0.0858867455925032	0.815882232107197	0.0581989864002405	0.868466014787387
**Nfil3**	-3.31817874819433	2.99895008086127e-20	-3.25740694775071	1.16243727170408e-05	-3.31938481290246	8.35092106905689e-06	-1.93060297279379	2.72743560024017e-05	-1.87319896090087	0.00855251471412606	-2.172970860737	0.00124105719533871	-1.61532725858618	0.000231703602917219	-1.54699781835692	0.0126879599633874	-1.61625645451372	0.00179484315536396
**Litaf**	-2.04137327684228	1.03778619023986e-19	-1.98099748631797	6.20767595717631e-06	-2.03035453621873	7.86672124889252e-06	-1.0684322117695	0.000303901233897763	-1.02305767775087	0.0109862164077153	-1.10520057941436	0.00526996105594141	-1.35003384064488	3.81535230971466e-07	-1.28364363081036	0.00250337348097098	-1.38449125018217	0.00119575545770063
**Vps37b**	-1.82176706171798	3.44645857176193e-18	-1.7592894047797	6.69432423087399e-06	-1.78170771877997	7.86672124889252e-06	-1.94419690833827	3.77058276532801e-15	-1.89825420811634	0.000122083323879367	-1.9787312810102	8.269676597756e-05	-1.78229000839919	6.59939977240091e-15	-1.71133347979739	0.0001428242724116	-1.77354724143845	0.000103247892277572
**Gem**	-2.03364382683504	3.44645857176193e-18	-1.97235299543041	1.16500363876819e-05	-2.02388398481119	1.86244364325591e-05	-1.2445563743761	3.67107934204725e-05	-1.20451358389085	0.00717976638997489	-1.41383954647974	0.00276311206924827	-0.175073084696587	0.676215979296419	-0.108625597376941	0.804331033471681	-0.173675362826157	0.61296630411977
**Irf2bp2**	-1.35923010793186	1.05820001538447e-16	-1.29867512566706	9.3523009081234e-06	-1.36381219740934	7.86672124889252e-06	-1.0597348926765	1.96222698171112e-07	-1.01312873165548	0.00130869316300837	-1.0587307092335	0.00104438912306263	-0.739755399511143	0.000253947246719706	-0.671897863439412	0.0096784764327275	-0.72263596536794	0.00624828236291838
**Cd83**	-1.13344101759349	1.29279234519667e-16	-1.07823844556166	1.07819615446946e-05	-1.13145874422739	6.88374543887632e-06	-1.17301670781527	5.08329862815375e-13	-1.13217709834583	0.000193347024322987	-1.21198950137889	7.70744410519003e-05	-0.850877687859609	1.27889799431602e-07	-0.787279074127072	0.00198453718894313	-0.840165310031292	0.00119575545770063
**Cytip**	-1.34278449947267	7.83573656168983e-17	-1.28223546757477	1.12985099296593e-05	-1.31247290744572	1.35558421894324e-05	-1.23667954922073	1.64221524968841e-10	-1.19080670340866	0.000364287069033122	-1.20935210712255	0.000502253967394178	-1.02950870840055	5.8688160066784e-08	-0.961021926557967	0.00198453718894313	-0.990800842337588	0.00179484315536396
**B3gnt7**	-2.56128884816444	1.05902949559094e-16	-2.49524934977041	1.4960472814796e-05	-2.56265800702317	2.28980530785224e-05	-2.80285455273352	1.21064184543976e-14	-2.75654191960429	0.000230361962000272	-2.80213169114209	0.000502253967394178	-2.72597721029553	5.39948614050537e-16	-2.6535764806901	0.0001428242724116	-2.65288316434447	0.000448049760568061
**Pmaip1**	-1.76816937040269	1.64303695877265e-16	-1.70938798695105	2.5211057003631e-05	-1.79848633419236	9.0844466703e-06	-0.916064091370106	0.00153791787781822	-0.877543369456588	0.0252434363233612	-0.982207503230714	0.00979509350295841	-0.189233269413836	0.613113615461883	-0.125270496054027	0.754838145105888	-0.195233240490887	0.523088161555186
**Ppp1r15a**	-1.67888628968571	8.85738384908618e-17	-1.61741037256791	3.28403109412716e-05	-1.67069781485323	1.86244364325591e-05	-0.619607698270947	0.0341009062283833	-0.57731850195055	0.104914082975282	-0.700711636304365	0.054829399623452	-0.0779842905379926	0.846500114374887	-0.0118621371454681	0.979162538230422	-0.0594802163937419	0.877044549383313
**Il12b**	-2.80933051999602	8.0529566691956e-17	-2.75208724216253	1.31951752991575e-05	-2.86008057395694	5.49832352152148e-05	-1.7333297253634	5.60508417513621e-05	-1.69109527429594	0.00751286362845955	-1.76171891009779	0.00512203496682248	-1.29196799902208	0.00172624804820162	-1.22780104200785	0.0208611198054175	-1.28500077738722	0.00757323000038996
**Tex2**	-1.42989172643608	6.36323137273382e-16	-1.37192271932365	1.91748034490354e-05	-1.43536824960681	8.35092106905689e-06	-0.39888907450381	0.134477386475804	-0.356443535786422	0.221415124236041	-0.390395933273923	0.123366175977223	-1.1169920954502	5.8688160066784e-08	-1.05210865314288	0.00235585726559431	-1.12760056314458	0.000995685637230599
**Cd69**	-1.71732243	7.58749421035637e-16	-1.66168955	1.91748034490354e-05	-1.70985987	2.29364073081565e-05	-1.003074498711	0.000324520901207853	-0.96103493334	0.0127995074614026	-0.99518780120	0.0105276090969099	0.330049476695088	0.309921630802432	0.39363972146668	0.224733862336628	0.369576210256355	0.191039312826866

	434262		444117		259365		58		981		8173							
**Nr4a1**	-2.53029167856434	1.66285957148499e-15	-2.46627051513784	2.82291984814142e-05	-2.55617662599251	9.0844466703e-06	-1.90024274994775	1.87590141884612e-06	-1.85379662296102	0.00346960674980325	-1.95648194660627	0.00119091568208696	0.33006418866535	0.538865471339202	0.394828846712608	0.421658052278058	0.24556367115759	0.578073147043615
**Cd274**	-1.16467590382815	6.86925653379866e-15	-1.10664726516704	1.67821936525709e-05	-1.15581550728172	9.48741040685052e-06	-0.827022627454448	1.36097432615113e-05	-0.783748646164649	0.00403913872791058	-0.870109177084938	0.00159499969919324	-0.359184705485708	0.0811809422917733	-0.293340599977301	0.178984986487408	-0.352655774992925	0.0756565808836603
**Adora2a**	-1.42873550489201	3.66490924108631e-15	-1.36704989382015	2.29319045282027e-05	-1.4245425890182	1.41146017925408e-05	-0.412418920855319	0.126717745881662	-0.366831118219346	0.214340160203215	-0.418988169093265	0.111794207500327	-0.584285419064354	0.0113278862862479	-0.516156714798999	0.0662566152011937	-0.601584939310124	0.0161775244500567
**Klf6**	-1.5726350083162	1.86539416465312e-15	-1.51710188310015	3.46185998851356e-05	-1.50795344978664	2.79321193217332e-05	-1.09672873501365	1.44098293295806e-05	-1.05689007373233	0.00708034899419556	-1.04635671297473	0.00563916579949571	0.00642445472537784	0.989643193426014	0.0679443726020531	0.868116145349229	0.0514724422479843	0.881976123268142
**Cebpb**	-2.49007279682423	7.01611236620508e-15	-2.42327509745795	5.58171887181358e-05	-2.48849178960528	2.38876325507916e-05	-1.91345499612439	1.52725947787317e-06	-1.85609545428252	0.00565921462367517	-1.97576519966018	0.00208787985897363	-1.23641601931146	0.00181406717864384	-1.1656396277051	0.0306532354330621	-1.21103945079268	0.00700658075677624
**Serpinb6b**	-1.91426780780872	3.42116524369106e-15	-1.85666625353948	9.63337817103851e-05	-1.90492445860722	4.5916609906589e-05	0.0660905856400008	0.901670621629132	0.103818351227235	0.84702428194272	-0.04873649192201	0.93063000025142	-0.604352965870453	0.0712886607901468	-0.539400284090411	0.194495078664355	-0.671928562169379	0.0817924028462642
**P2ry10**	-1.44812636706178	2.21874800693397e-14	-1.38442756659116	3.02902577236286e-05	-1.42707817760587	2.38876325507916e-05	-1.67775885527045	3.37623435711537e-14	-1.62735456404601	0.000193347024322987	-1.63553460631555	0.000116001717630597	-1.09755009472868	8.95686643001081e-07	-1.02761979963137	0.00294661378218721	-1.05856484375955	0.00237125864146425
**Ptger4**	-1.60202961991075	7.42858894020388e-14	-1.54226525493504	3.28403109412716e-05	-1.58778715528189	4.44213072790652e-05	-0.778899718543406	0.0078568195902368	-0.736336533947089	0.0399989489143465	-0.792795040012787	0.0277351424659676	-0.482413364837724	0.10287540642327	-0.41706857153848	0.194485146243693	-0.494480240013133	0.0806520865849337
**Fos**	-2.21022525029951	3.00619655690572e-13	-2.14819033286739	4.32705667331822e-05	-2.21185517065919	8.95935257105811e-06	-0.626413655922994	0.181394502406265	-0.583056309881958	0.210740095620142	-0.566269931072967	0.169571546785984	0.179114703523861	0.749721588058389	0.242286366255274	0.615024748013074	0.229708200571257	0.558478751375046
**Btg2**	-1.15619977171502	1.53037740682781e-13	-1.09496224892515	4.17508809594781e-05	-1.15576520904074	2.38876325507916e-05	-1.00494101343539	2.05637243228704e-07	-0.958795098720581	0.00179843306965406	-0.986006245552993	0.00129617832470211	-0.0982706903565791	0.732694357191899	-0.0329635148446959	0.915275471696029	-0.0834438105786288	0.734829008925667
**Tigit**	-2.47649942628895	5.9258493047312e-14	-2.40943970239469	2.8888877428284e-05	-2.41290788138287	0.000143204313756384	-1.39642685391056	0.00126716725024873	-1.34925498690032	0.0186291033768396	-1.45534581988865	0.0118739002204113	-1.80636809189418	1.94909563698493e-06	-1.73306195840158	0.00330251942803938	-1.83983706106757	0.00246042819851195
**Dusp1**	-2.21608150785312	9.55732597378175e-13	-2.15627109560543	4.84298090760151e-05	-2.33754323559684	6.88374543887632e-06	-1.86406353174982	1.31890359561763e-06	-1.82036808496768	0.0026195986136617	-1.91819216997033	0.000559361803615894	-0.686385198683617	0.115095974991263	-0.620649507217846	0.16689783337343	-0.680567296064169	0.0749624803915623
**Dnajb9**	-1.52551270049716	1.94031162693475e-13	-1.4611431658606	4.58725172170281e-05	-1.51181844644767	6.11059917775613e-05	-0.679760954294228	0.0188789018488343	-0.635693724834285	0.0636204871061107	-0.720375595893963	0.0436650650797463	0.307417554696058	0.33216350561618	0.375399596533594	0.23058258380685	0.322602659088762	0.251747883330888
**Anxa2**	-1.45883409810703	3.7340506590909e-13	-1.40159020877489	4.84298090760151e-05	-1.44691766933585	4.5916609906589e-05	-0.173998514869434	0.639229284903074	-0.130803890920066	0.725310561585262	-0.192136652040115	0.55479443744152	-0.859204139842264	0.000469256654703453	-0.79504788495001	0.0142028056282986	-0.879308731303761	0.00666744961982888
**Gpr132**	-1.36289741938003	6.02197678858362e-13	-1.30134432681272	4.58725172170281e-05	-1.346595488185	3.22946405961422e-05	-1.31638773729576	9.32326568250728e-09	-1.2698020104252	0.000845581566003926	-1.29080875303642	0.000923101651125473	-0.751409749141882	0.00132137677401967	-0.68376571857919	0.0205870930193371	-0.773231636245502	0.00870707534811546
**Ctsl**	-1.81471901856969	2.59229153994825e-13	-1.75925190803425	6.13406477445806e-05	-1.813084181219	5.84274904009147e-05	-0.383475132657173	0.344761842088027	-0.338913771813345	0.433042170025463	-0.393809725833453	0.29265613493568	-1.07209122095314	0.000389561478238641	-1.00767274157466	0.0146744613638367	-1.097451786383	0.00624828236291838
**Grn**	-1.12513351707725	7.21033771058587e-13	-1.06920038680114	5.58171887181358e-05	-1.12286675315985	2.6774531126004e-05	-0.292482912521415	0.241042650887399	-0.248833580396166	0.339967007503442	-0.317032494373328	0.198377163764497	-0.815853521266034	1.30464742286885e-05	-0.751464686144057	0.0056631480703662	-0.808153888434094	0.00317316819447119
**Ctla2a**	-2.30312643765541	3.13177513052706e-13	-2.2407980901965	4.58725172170281e-05	-2.29632982073708	0.00010552343847559	-0.803055346472059	0.077464151686639	-0.754992455175434	0.137310187337858	-0.843158233643798	0.0682942764820969	-0.915653248782454	0.0235974408901612	-0.849439170166008	0.0767283682967303	-0.940852988347688	0.0232007570833069
**Myc**	-1.83397651274125	2.00269412958057e-13	-1.77576825429661	0.000173614209852289	-1.83607412537087	6.37355997648071e-05	-1.56191923756188	3.65618789835379e-07	-1.51779900527674	0.00558627618344427	-1.55351016480729	0.00275639919608607	-0.480713198794721	0.180638631907927	-0.416143467723281	0.330751748428653	-0.540453615562028	0.148038913775533
**Ccr7**	-1.59434509043115	2.05045486039258e-12	-1.5402407400832	0.000131749573750212	-1.56075893157533	6.24891111776238e-05	-1.25305037751887	1.15199931108026e-05	-1.21299574120808	0.00717976638997489	-1.27051213548117	0.00352732904278951	-0.703238974081917	0.0167571770036984	-0.639846058776678	0.0819429209057193	-0.740605252076903	0.0319409071979359
**Rab30**	-1.4854098465106	3.56936552828498e-12	-1.42382704969187	4.84298090760151e-05	-1.4379172439318	0.000154004965575292	-1.73478619400532	4.63019806016933e-12	-1.68810236016115	0.000277821032242069	-1.68994558534899	0.00101748997815111	-1.30103022386766	1.19768869617193e-07	-1.2329797086884	0.00198453718894313	-1.25543871068869	0.00246042819851195
**Ccr5**	-2.2587736140984	7.37984226024176e-13	-2.20056619227572	7.22949784919115e-05	-2.17886826187358	0.000519085594937192	-0.316073275424255	0.570744062813537	-0.276400512543452	0.630069696381444	-0.322204549537787	0.529674871519908	-0.699570031343336	0.105383780862252	-0.633942867494876	0.190160239169736	-0.75148539780274	0.0959016964841651
**Klri2**	-2.59279514187309	5.45619428529289e-13	-2.5362892062498	0.000130660767420714	-2.52141458145782	0.00118184828684624	-0.573093413883128	0.318206704601067	-0.528435878892077	0.408646735557907	-0.583842813152439	0.339384467930737	-0.512443983707401	0.348057411758437	-0.445376462119693	0.45843327156945	-0.586576924929944	0.262046559735641
**Klrc1**	-2.64772074898965	1.13598867943561e-11	-2.58173637964849	9.15647245117342e-05	-2.65415229819433	0.000202730163503014	-1.82134548489041	0.000237189558781814	-1.76733804794141	0.0121503263811975	-1.80496421823056	0.010030542050775	-1.33214105828533	0.00542295769896473	-1.25997636092702	0.0346883326618292	-1.41031226604578	0.00932106195007179
**Ptp4a1**	-1.17364309615041	7.604175853486e-11	-1.11707451353042	9.59526286219995e-05	-1.2028233094459	2.92703896270223e-05	-0.980623929221168	1.36097432615113e-05	-0.936371254336825	0.00450817389859154	-0.997700613856147	0.00208263495925281	-0.376818223156069	0.128061489339222	-0.311993617976963	0.234757998812364	-0.367463004168705	0.089856001924514
**ENSMUSG00000105160**	1.86719603853348	1.72649120673792e-11	1.92329092201835	7.64761235519088e-05	1.89323629923538	0.000162263405501454	0.903448763876902	0.0192368197683963	0.942542651349889	0.0402192873528051	0.88783515777663	0.0984795302548978	1.50325721844723	2.67301474100492e-06	1.56450520764113	0.00258396353296751	1.4766496599668	0.00525371475903645
**Bambi**	-2.58066257167678	1.06055817646379e-11	-2.51552299453468	7.64761235519088e-05	-2.52524103039345	0.000290431598259208	-1.43035599865851	0.0037989008207442	-1.38513828873058	0.0252520301853721	-1.57127807491716	0.0128180890829517	-0.741514822418146	0.143088043287462	-0.672953179032188	0.194485146243693	-0.651276255758427	0.122539901084725
**Jdp2**	-2.55711239402254	4.27699123955324e-12	-2.49489126714159	0.000110986114401082	-2.56978281294298	0.000585998025028289	-1.5279085569194	0.00160720426703259	-1.48466381897939	0.0252520301853721	-1.52154091086664	0.0286159965233741	-1.15869783454224	0.0127735471883985	-1.08983124219618	0.060448130071287	-1.1083768922725	0.0410153363339683
**Thbs1**	-4.34759229193269	1.62789285279074e-12	-4.28208689700336	0.000167711288116775	-4.63511663699164	0.00103534214468113	-10.1721359824274	1.3469093629912e-11	-9.87951009370484	0.000193347024322987	-8.9958718872356	2.2736112938443e-05	-3.87212135864421	2.98237632656283e-08	-3.8136480392172	0.00258396353296751	-3.86676003926977	0.00359384083136747

**Serpinb9**	-1.86493714997498	1.13598867943561e-11	-1.807864655915	0.000315649632438819	-1.86116188659509	0.000173274839355271	-0.255740532045136	0.606814543577459	-0.219616873679663	0.684074308443701	-0.407651413200815	0.363661721552193	-0.57869606099084	0.132023172312947	-0.514564262794049	0.259132027526375	-0.694283849123892	0.0849386958736228
**Rhob**	-1.75157945195978	1.06055817646379e-11	-1.69153016757803	0.00025483544524354	-1.67325954669808	0.000288393904203606	-1.04972912473756	0.00214948495173248	-1.00996650724798	0.0336279166973048	-1.08512653245748	0.0258148929108197	-0.270086035538741	0.526403848188353	-0.204471053287346	0.662935324206282	-0.219096282751202	0.596414645128905
**Eomes**	-2.11391055706661	1.06055817646379e-11	-2.05477057422693	0.000337117472269478	-2.0961592332739	0.000249011907834907	-0.704031642319161	0.125055436175859	-0.659701770759401	0.232920009766702	-0.732258356834543	0.141916514163573	-0.915896672014368	0.022808848520567	-0.846574353441908	0.102875276500257	-1.03740404071152	0.0288414644988357
**Per1**	-1.85995347955407	2.01975462878312e-11	-1.80110638431625	0.000411922596400193	-1.80619718255283	0.000249011907834907	-1.86666130636929	2.23848790903209e-08	-1.82763867177646	0.00356071687173101	-1.86055756640205	0.0024331867301368	-0.55131989916449	0.159895592654086	-0.48591216773325	0.306967084292952	-0.513876193821473	0.221750550387287
**Ifngr1**	-1.15054279127714	1.68346825454326e-10	-1.08803277250188	0.000130660767420714	-1.11472723035826	0.000144477697006661	-1.24267124112967	7.45432103246863e-09	-1.19259469386582	0.000760788030789109	-1.20846988403168	0.000854472923213835	-1.15929993045657	1.84356843118443e-08	-1.08875842681345	0.00198453718894313	-1.11545386232241	0.00159338639086515
**Mxd1**	-1.5747159527743	4.52109176393433e-11	-1.51865917574936	0.000459669300313365	-1.54256149403637	0.000173274839355271	-0.392397286420277	0.304965018219067	-0.356372440299756	0.430625570583205	-0.430075303823672	0.287887653105142	-0.418829268195557	0.229047830836088	-0.356673613763853	0.39473126196048	-0.42617866395372	0.2398897249182
**Bcl2l14**	-2.50687866071527	5.52055158064058e-11	-2.45075688118338	0.00042405292057341	-2.45900885910438	0.000522816543966231	-0.989225732402052	0.0696936555888348	-0.961396899016041	0.156173376029002	-1.14358729701901	0.0594401432752619	-1.55125463665469	0.000792027302670682	-1.49435673435091	0.0261720100022732	-1.61073672662426	0.00932106195007179
**Fam107b**	-1.30120381919502	2.42312161905468e-10	-1.24060981268237	0.000283674323527972	-1.23182617429132	0.000399042175271125	-1.46321475520251	1.64978175277975e-09	-1.41601826078029	0.0010870054841503	-1.38565365476081	0.00144733568711509	-1.36517303161753	4.86169985075454e-09	-1.29607868917864	0.00198453718894313	-1.27747220140617	0.00213205519032263
**Fam111a**	1.01867101113871	2.62764554153013e-09	1.07603991041342	0.000131749573750212	1.02537945171768	0.000144477697006661	0.400142567849184	0.108243940131746	0.445696299350245	0.0968465562739727	0.409872716119238	0.135061398027262	0.584458811424919	0.00611802461797047	0.648673642511113	0.0152181550513476	0.575875563903784	0.0247306754629684
**Snx18**	-1.22998858297032	1.73589586323977e-09	-1.17049832163396	0.000269825042077325	-1.22225151165477	0.000123154206128718	-0.498457588524761	0.0891599423310693	-0.453771972791239	0.156050638400093	-0.507256121723877	0.0876520469337403	-0.682632537911774	0.00675796318071093	-0.617270476351001	0.0437984128739164	-0.679771211521842	0.0150932442491428
**Rab27b**	-2.87919251318875	1.34985572474778e-10	-2.80357510517804	0.000218292784592241	-2.62250957465852	0.00345813845252537	-1.67751003884899	0.00452651219901054	-1.62028324861833	0.0361641075770965	-1.45980217844115	0.0734714201530886	-2.31600366302349	6.70436083069226e-06	-2.2356031987105	0.00519687621506871	-2.20373304893875	0.0111380629159527
**Jchain**	-1.46415518656227	1.14764961165326e-09	-1.4073845131685	0.000673979280564047	-1.53064925609401	0.000136713329093432	-0.0801516455423905	0.87395541085099	-0.0343507379499274	0.954574892626516	-0.0751406578901745	0.876178947673196	-0.758109709252584	0.0127735471883985	-0.691037119819066	0.0857878915058446	-0.802210586672876	0.0290094807968121
**H1f2**	-1.71210411747003	3.05599770668782e-09	-1.65857485298348	0.000234709796054196	-1.72888335020504	0.000160133845113964	-1.36741222475077	0.000154973739138409	-1.32709183476196	0.00879967713021352	-1.46557200127808	0.00512203496682248	-1.3067512951745	0.000116120225170135	-1.2411827220809	0.00827183148337801	-1.25678421376856	0.00503418430344143
**Nek6**	-1.7833538830385	2.41442703721435e-09	-1.72504972530309	0.000305213729142687	-1.81939583370631	0.000168979337730591	-0.416510196668632	0.388771543190753	-0.374549916430819	0.454109363477683	-0.425930246044316	0.28163018309339	-0.522271646754471	0.210898586275292	-0.458225481206142	0.308076570309357	-0.621518892553246	0.0775541015273506
**Herpud1**	-1.14744452365341	4.78560919849125e-09	-1.09119640135243	0.00037036091511065	-1.1772330342005	9.626063450996e-05	-1.28902633798338	2.82230721058562e-08	-1.24351700172836	0.00130869316300837	-1.26975420789264	0.000825016567990643	-1.07056152576568	1.71293992788202e-06	-1.00675351766378	0.00364018834343927	-1.04934606982985	0.00179484315536396
**Plaur**	-2.50463665585327	6.8335598344409e-09	-2.44332154380617	0.000394174519261009	-2.45114782136606	6.38734375263579e-05	-3.3302527947125	1.96288212233071e-11	-3.2770736014313	0.000791479522458753	-3.30581933834458	0.000446348686195453	-1.82691381286693	0.000383616209289231	-1.7580078645776	0.0114605030847885	-1.87961393200724	0.00296411756678496
**Ccl5**	-1.86629136915372	6.11085681249197e-09	-1.80486683700442	0.000327009634147332	-1.86273114319221	0.000154004965575292	-0.557453215189819	0.261815299135447	-0.515564476854361	0.297509114366382	-0.627917892317352	0.18280940097765	-0.0664856425999295	0.915947147197804	0.00058352720740341	0.998615208777374	-0.157853725417205	0.743126695440944
**Plec**	1.28718060422294	4.24969379104675e-09	1.34553886536338	0.000342488433962586	1.28185374084663	0.000330753796265709	1.17878026913372	1.20155074259194e-05	1.2191524312313	0.00367764193850275	1.09937368602855	0.00787344899270234	0.913893622108342	0.000496021727002311	0.976955281932793	0.00950050390327811	0.901677179985306	0.0139949100455182
**Atp1b1**	-1.35481295064161	4.76355259580291e-09	-1.2990020476376	0.000377210894905221	-1.32002484313128	0.000408865946530728	-0.295987098778213	0.442826860850898	-0.253030575581374	0.52486940096874	-0.275962794947439	0.462838704520981	-0.592948179891499	0.0490427157832905	-0.528389457093156	0.125543857093319	-0.611302294753954	0.0650969137976316
**Id2**	-1.09467837153571	1.24207972956864e-08	-1.03737085935632	0.00044115374515351	-1.11473423419017	0.00017652817488279	-0.453199809804111	0.100528597303736	-0.411572283817833	0.170888174080287	-0.484350417271094	0.105269303324123	-0.449644933626532	0.0803119978431297	-0.384218318763108	0.174982699734634	-0.477534226059448	0.0756565808836603
**Srgap3**	-1.37378820189227	6.73106761878141e-09	-1.31667441041877	0.000368008206282566	-1.35116494596075	0.000394798765910928	0.276768853564132	0.481915886962228	0.318221444293286	0.407099345869549	0.30518308940883	0.331225124805234	-0.500235691387774	0.114912643679349	-0.436770202572842	0.213288344115012	-0.509708611034279	0.0963909668169736
**Dusp10**	-1.37822951603171	5.84393754494089e-09	-1.31412742013187	0.00024536455243111	-1.30793841805896	0.000782658730824735	-0.961257884126605	0.00165504148800084	-0.910583503905601	0.0188858332845946	-0.891280853789257	0.0367776134532736	-0.717282956095123	0.0151671475634766	-0.645308116019497	0.0571170851063383	-0.694789393138012	0.0450176114139778
**1700017B05Rik**	-1.18068708384469	2.6754277152184e-08	-1.11948196087076	0.00044115374515351	-1.17548617238295	0.000135526731385919	-1.32589079973969	1.65756267522160e-07	-1.2779341433623	0.00170654722198551	-1.31135848585798	0.00107839594793348	-0.68825752148023	0.00817522841124309	-0.623743632876617	0.0453320576411499	-0.676763251480234	0.0141234238098208
**Gpr171**	-1.09944172106953	4.48711404143321e-08	-1.03654594561639	0.000363727997439259	-1.08818464561956	0.000168979337730591	-1.88659889394362	8.93767604119168e-17	-1.83853419933862	6.27406623717596e-05	-1.91281466458522	7.98549596377835e-05	-1.09417247484358	1.2186931731187e-06	-1.02613555458454	0.00258396353296751	-1.07667273202281	0.00163955316281712
**Plscr1**	-1.86251218676696	1.06756722234582e-08	-1.80779159512224	0.000327009634147332	-1.81845470791941	0.000834087838250891	-0.94669489625953	0.0328848648299121	-0.902382719374262	0.0772458434350107	-0.861057184694117	0.0913417391492545	-0.905260020563312	0.0255877665258174	-0.839573430503055	0.0772452067747407	-0.857331740963308	0.0505371755907382
**Tyrobp**	-1.56743374723899	2.26485777508977e-08	-1.50540421223359	0.000494830830181734	-1.59028510673111	0.00036952162656154	-0.730205966939404	0.0617373979944809	-0.684154626006133	0.124446287386291	-0.717773654774291	0.0907110974180144	-0.530951355402008	0.165576837370296	-0.464415567748582	0.26418245813876	-0.555745746596886	0.111336485877856
**Spry2**	-3.22653373446775	2.05978204926563e-08	-3.16971071255799	0.000357071279027317	-3.18338528887123	0.00102254866897176	-2.15083258739858	0.00435149482952493	-2.10582998221013	0.0247574068985899	-2.55606051314569	0.0129116080302428	-1.03153384666292	0.199041083085266	-0.963143397583496	0.211722647542587	-0.989258390341936	0.0915862656477992
**Rell1**	-1.43796357421488	5.83596759862778e-08	-1.37945374714048	0.000525007723830849	-1.43439251416907	0.000296027358746958	-0.695439937615309	0.0584881618433448	-0.648113985154985	0.116710417798494	-0.680932239704201	0.076550557764864	-1.11324040008491	0.000312652118063915	-1.04731111998522	0.0116774114332987	-1.09857971942803	0.00624828236291838
**Dntt**	-2.10255162048004	3.25845094430021e-08	-2.04700776492293	0.00044115374515351	-2.1179453199877	0.000680639745203143	-0.448237308040324	0.47307730945235	-0.40496624686482	0.515310341892703	-0.664781878347667	0.189555091128933	-1.61449141019694	0.000251123343547389	-1.54875169028305	0.0096784764327275	-1.63455030606983	0.00624828236291838
**S100a6**	-1.32578127156098	6.18429283809987e-08	-1.26624326874271	0.000442356039485683	-1.31456343235737	0.00036952162656154	-0.412813532357113	0.263383462379071	-0.368544124248521	0.331996199308339	-0.521579398467326	0.129771321428029	-0.761748440274768	0.010420706322597	-0.695885341422737	0.0487105016364895	-0.766466588589656	0.0208938826300542

**Spty2d1**	-1.15981216200695	6.81862540948335e-08	-1.09871895956252	0.000623916767556555	-1.17104486910107	0.000280194755870619	-0.765593944183114	0.00726234845120251	-0.719160580941236	0.0399989489143465	-0.752364871714835	0.0277554104248715	-0.22043330143714	0.520762775271888	-0.155216327414593	0.662894858297566	-0.247168138267919	0.370075513258532
**Gfod1**	-1.52193795016539	7.47124498891526e-08	-1.46107795006887	0.000609922738864101	-1.51818620582081	0.000402158238899232	-0.714702465613975	0.0683879255438233	-0.669365424846324	0.131318291780868	-0.686259337654867	0.0913417391492545	-0.471545962532036	0.22448243475631	-0.40784433547064	0.332199390111813	-0.445227861277001	0.170046469998783
**Fgl2**	-1.13213747076313	1.03381250160927e-07	-1.07661387012986	0.00064369134854488	-1.13361044037545	0.000311616929743483	-0.543699247496775	0.0670138504982106	-0.50167993791933	0.129778884863354	-0.536217535728055	0.0827492009327586	-0.479580967894405	0.0873630145114663	-0.415923300057156	0.179270207389431	-0.47630619241425	0.0755610711253508
**Gas7**	-1.69659183106574	4.0968216530538e-08	-1.64225573392853	0.000525007723830849	-1.64351056398423	0.00125667085243776	-0.406715954734856	0.415083234725843	-0.367721926729375	0.476002977992287	-0.460720185656026	0.331981827396764	-0.630325757371608	0.12848802900263	-0.570342783289339	0.209282240959411	-0.572588601401962	0.161254722707397
**Klf4**	-2.01776476594576	2.16606886465922e-07	-1.95129004891551	0.000498785842181648	-2.05158886030354	0.000296027358746958	-0.499645200885347	0.432836760797495	-0.463087193539115	0.422330641221017	-0.640389510223027	0.1854723138205	0.331079114004539	0.604824635869466	0.396802223772709	0.463920563907482	0.347886421536853	0.401323442847635
**Emb**	-1.40705307524396	8.32965442595649e-08	-1.34277074053798	0.000764246064811074	-1.34098239848486	0.00108063497675159	-0.5031213847876	0.198616691544077	-0.451949410933041	0.286059682328374	-0.453266788001889	0.279514858321168	-0.938696396281821	0.00292684431862967	-0.866666827715707	0.0332016451676374	-0.931840059400282	0.0233068699535049
**AW112010**	-1.42885673210229	9.9490740713148e-08	-1.36825488769052	0.00121315514305105	-1.44085691748726	0.000605846328637251	-0.644934416370911	0.089279635949278	-0.603270426431708	0.177249950671008	-0.709346520316635	0.0965267012174702	-0.385227268736103	0.329810918232557	-0.319632103798122	0.468846140411895	-0.502754350566031	0.167233782384947
**Frmd4b**	-1.85202965751387	1.14958754159642e-07	-1.78475984641136	0.000675658039198376	-1.81942693875171	0.00102259585486623	-1.18499161596566	0.00988082922045156	-1.12999802877719	0.0480366194759740	-1.15868782717607	0.0464284018789028	-0.603621456992515	0.210037791718245	-0.531149238259896	0.302370662363704	-0.623647890797873	0.143302844855995
**Siglech**	-1.62094301869445	7.00321205233502e-07	-1.56498825211399	0.000525007723830849	-1.56804895841129	0.00022662637400054	0.0433670764308872	0.949345716686345	0.0844389082960203	0.877568746169473	0.0192468318284024	0.972056183556957	-0.759940733443992	0.017309702558932	-0.697231947267838	0.101622757705863	-0.745256631945155	0.0541592130429092
**Hhex**	1.1549778733446	3.82052991602291e-07	1.2123411216221	0.000673979280564047	1.21603516394972	0.000402158238899232	0.89343034620706	0.00218319056306104	0.936834487529105	0.0157745498868776	0.963220541711164	0.0134052951874248	0.912337688777465	0.00066300296493138	0.975809415768585	0.00938201880677654	0.980814378288037	0.00700658075677624
**Ncoa7**	-1.05821099421061	2.50931236154535e-07	-1.00280797205172	0.00100608285893961	-1.03635906432958	0.000476568455287032	-0.334506894227586	0.288400796794571	-0.295106595749482	0.379711122759674	-0.344882833687193	0.260718320343293	-0.557544601184945	0.0323718460818784	-0.49394961757796	0.103739999163719	-0.599395206571884	0.0373729988174825
**ENSMUSG00000108292**	-1.34049455163598	2.16606886465922e-07	-1.28357610846901	0.000652836443704175	-1.29528870965617	0.000878755110595172	-0.182910123741253	0.693923936652214	-0.139878953609593	0.759370845905713	-0.181063629916897	0.643257006142962	-1.1076975176319	0.00019727926980723	-1.04521327791238	0.0096784764327275	-1.06000062532327	0.00726089061074744
**Samsn1**	-1.19093348014852	2.08593898871809e-07	-1.13649578512373	0.00113119179910181	-1.17315075866299	0.000613307144351515	-0.196720032095834	0.628145946395767	-0.159239093783759	0.705397104573377	-0.260475479212887	0.484063016157129	-0.733518477879708	0.00933227765988472	-0.669785737972543	0.060543852173206	-0.778777395701737	0.0237530022158686
**Plcxd2**	-1.70132947103876	1.56719047108848e-07	-1.63471744754022	0.000615150934812872	-1.61728857684244	0.00165268359421697	-1.97216412629918	2.8169728140968e-07	-1.92033455355763	0.0020614538929574	-1.89191147103172	0.00858700275306178	-1.20267318939307	0.00165374655404251	-1.1296582456075	0.0204696273794423	-1.21734203151864	0.0165632891733874
**Bag3**	-1.38836518404252	3.38354875900265e-07	-1.32663708319817	0.000778167219591534	-1.39869873519463	0.000836920215495676	0.221307915961799	0.640344254576993	0.264800113403659	0.553646763447438	0.116651517218822	0.787678871564803	-0.573320010790998	0.109642535794218	-0.50596485969958	0.194395362626552	-0.621007240477056	0.0806516169626725
**Myh13**	-1.7916558374915	1.18551820973763e-07	-1.73593519519719	0.000609922738864101	-1.7081500748096	0.00370393048447722	0.518967118953812	0.303688528265182	0.554436816027423	0.275625374930090	0.485786517983172	0.278653676826458	-0.934702920023494	0.0239266547398099	-0.874199239090769	0.0721365821918845	-0.880513040854455	0.0725795492846912
**Irs2**	-2.08116693163289	2.12128627451161e-06	-2.02422781031611	0.000729183563526702	-2.10662506889685	0.000203826203819212	-2.35666502184181	6.86973599989885e-06	-2.33130296692165	0.00296831691924805	-2.59528984540159	0.00115504083990569	-0.719052813372032	0.241393731070934	-0.653809754772058	0.253148352505856	-0.874667199132562	0.0536955994652746
**Trp53inp2**	-2.36103912113579	1.41032268902261e-07	-2.29893712741745	0.000731115734607341	-2.30681626875663	0.00328493422285501	-1.93293910792278	0.000564202886842262	-1.88922741714067	0.0167577931756727	-1.84451512965543	0.0332007830395922	-0.82830904505722	0.166745238842539	-0.764792511550055	0.234757998812364	-0.88983381477746	0.12392576754652
**Csrnp1**	-1.22113185555848	5.13163221256295e-07	-1.15906771755682	0.00113119179910181	-1.2087331569878	0.000762656679315343	-1.07257683261963	0.00039134914180367	-1.02060087008654	0.0153486220924168	-1.05318330263204	0.0131608848663991	-0.136802121616852	0.748554024361149	-0.0693115767699346	0.882768304846599	-0.138873165869382	0.689391397373187
**Sla2**	-1.39800728514759	6.70863518646039e-07	-1.33714371182585	0.000910271799687227	-1.38156514694759	0.00081728576184155	-0.306778500971474	0.506518391401112	-0.261888146893774	0.574068071522870	-0.307032108763496	0.430536443067035	-0.984262714607564	0.00292684431862967	-0.916708573109159	0.0279006418257681	-1.03238798067746	0.0110339723890773
**Rilpl2**	-1.07030349448946	8.52110459581161e-07	-1.00907201771563	0.000918427747060252	-1.05794883129356	0.000878755110595172	-0.506925181158504	0.0963012869450433	-0.462107409289017	0.152669968057494	-0.533252052651981	0.104856925025944	-0.230621916432075	0.493891846953817	-0.163333993154167	0.626819901187592	-0.247054082661927	0.386554235644695
**Hmgn3**	-1.07337250286311	9.47600892980742e-07	-1.01575476966814	0.00086461537382731	-1.06660582181423	0.000955668940442291	0.407887851441829	0.202122828825356	0.44694007097237	0.157365821530320	0.277219123378626	0.362007074268851	-0.456701157556306	0.114420008472473	-0.392209192843906	0.195699799818648	-0.478858517837887	0.0963909668169736
**Ptprs**	-1.41499563350963	4.89707753107851e-07	-1.36152738032265	0.00110416504748258	-1.34918417714003	0.00213700083679591	0.15313347625194	0.773483438426553	0.188826248697756	0.705184774993674	0.199693931849856	0.652539185533322	-0.604717071856534	0.10287540642327	-0.545180706256701	0.190097427380602	-0.581876650385295	0.130859069779177
**Tlr1**	1.00294213535061	1.93099370359524e-06	1.06253794340612	0.000675658039198376	1.00026439992526	0.00103534214468113	0.260998314881076	0.453225517512703	0.304951225898593	0.359476974167655	0.252903388038116	0.504797304539784	0.988083573084431	3.65791879586377e-05	1.05265882910723	0.00353127874376803	0.959760551477115	0.00525371475903645
**Cox6a2**	-2.10198239982565	5.50449588965333e-07	-2.04226831511342	0.00121315514305105	-2.05561106078341	0.00203183510198348	-0.748903519353822	0.226734807635319	-0.704548767600555	0.298991985908399	-0.933562500826977	0.129589286365432	-1.10889063795974	0.0337893664007151	-1.04534079397095	0.0958861987386598	-1.14343512712381	0.0395059979090889
**Chl1**	-3.08802311847715	3.3242408599664e-07	-3.01217720583095	0.00122145438223325	-3.03282011942695	0.00361878283912766	NA	NA	NA	NA	NA	NA	NA	NA	NA	NA	NA	NA
**Dhrs3**	-1.7152929200357	8.48189424881735e-07	-1.64738712158103	0.00100252643326734	-1.63490694188116	0.00183537396290287	-1.29159915266509	0.00384358430727182	-1.23313233681278	0.0301288210893993	-1.22760085722146	0.0430737562044397	-1.02028985895597	0.0159161613849068	-0.947247619560759	0.060448130071287	-0.939877527399101	0.0453253448224276
**Tgif1**	-1.19981189693163	1.35410421481083e-06	-1.13821897428137	0.00113119179910181	-1.19434212693623	0.00102254866897176	-0.875371827777157	0.00667434247096923	-0.83310321859275	0.0354490975310011	-0.998237099264356	0.0178786651314678	0.0237726041981066	0.964336629828283	0.0904595678995606	0.834322343131541	-0.0110647901939911	0.974860835135961
**Cd79b**	1.02351810818216	1.93099370359524e-06	1.08213890137128	0.00100608285893961	1.07237111526314	0.000831041641194292	0.327948277792356	0.32519090685781	0.372072387006056	0.273053340652625	0.363986182366391	0.297144810495521	0.503330140429943	0.0717191104517533	0.567941176171304	0.0783406636442762	0.509369255355297	0.099799371964378
**Itm2c**	-1.20212499153608	1.23869802836731e-06	-1.14223950442309	0.00163896071159957	-1.20073892701756	0.000836920215495676	-0.0273598332248145	0.957651202933289	0.0145021781854844	0.979798138303763	-0.0647973400962338	0.885497444295974	-0.332438386449183	0.36251141082356	-0.267333867334369	0.497709869800622	-0.444229579465134	0.164863326234103
**Cd7**	-	2.041567046	-	0.000749702	-	0.001145450	-	0.373759824671	-	0.36523894148	-	0.31481686259	-	0.2774508172909	-	0.293229259874	-	0.122649459563

	1.98178000973873	82065e-06	1.91994567225689	513338999	1.91930107553173	82852142	0.588404709064345	157	0.539992369977425	6111	0.564658395757986	1738	0.645376458914591	64	0.574190153999183	931	0.766644267670139	249
**Prdm1**	-1.43306925607403	4.67915625617073e-06	-1.37373524481912	0.00181886482902598	-1.52144747039033	0.00022662637400054	-1.24948165676301	0.00149254515603019	-1.20043210596095	0.0225268594482433	-1.23523006889534	0.00998977766545106	-1.19063366623425	0.00101686872915241	-1.12495276771503	0.0187197084210407	-1.2029704159777	0.00503418430344143
**Mrgpre**	1.57572564422633	2.49373952646195e-06	1.63925566818851	0.000983471464061492	1.59286049975388	0.00118184828684624	0.596537052755909	0.231307186101515	0.640927454299447	0.20521341657413	0.399950412518822	0.527124733095642	0.973202909999422	0.017309702558932	1.04074970500675	0.0356552784417836	1.0157421774421	0.0468733852041469
**ENSMUSG00000053541**	1.39964579677907	1.74525632942187e-06	1.45293493316547	0.00166985749439772	1.48562117718445	0.00114545082852142	0.893687800598574	0.0220300651243186	0.931497331022872	0.0557733856619334	0.92792308270483	0.0724701935669873	0.827975190189843	0.0230174675925868	0.887553169551725	0.0572218846943586	0.799901400354864	0.0867964777491087
**Klrd1**	-1.76778798719059	6.70863518646039e-07	-1.7082388951907	0.00137017944603946	-1.7383346191626	0.00367125473557739	-0.336345904021751	0.579332038498612	-0.2920779891653	0.645447396279827	-0.32987254246232	0.578193405036165	-0.133289688402765	0.837824954162131	-0.069059944173582	0.92263770696848	-0.224801419856469	0.666595573810664
**Cdh5**	-2.06338636503909	6.82468035124355e-07	-2.00854780059491	0.00133400168219321	-2.03363049792519	0.0038756120838036	-0.0206779427914387	0.981241757184748	0.0135470156100718	0.988265281028821	-0.024594233901089	0.973835098791277	-1.33205133612652	0.0074655685570368 2	-1.27316342334392	0.0472913380553374	-1.4309157065441	0.0273552843544206
**Siah2**	-1.33435998508189	1.7352063941293e-06	-1.27063107828238	0.00117888487680253	-1.30557492891694	0.00174074657514443	-0.152766765246844	0.770469984147495	-0.113240328445994	0.822969208104857	-0.324160779744294	0.430430360114226	0.0164572268217582	0.978514936377786	0.081865614500969	0.872344174923907	0.0472501436986303	0.911420021004747
**H1f0**	-1.39077193780914	3.36449995852174e-06	-1.33788937957718	0.00129396840292783	-1.39493151091194	0.000834087838250891	-0.306169387132149	0.536989985032451	-0.265581044754227	0.591003915552755	-0.326600812874216	0.410536148710705	-0.6361337687733	0.10143895644636 6	-0.570646793280121	0.176381677571477	-0.755084442447888	0.0401807229227631
**Kit**	-1.52942710377736	2.00533848727783e-06	-1.47330218840373	0.00199641929105398	-1.56804599828467	0.000912575227475701	-0.600150795043005	0.204368280820352	-0.557753513340242	0.292411931752813	-0.58755128602418	0.19393124218496	-1.02420271700683	0.00831154601951255	-0.96071213408709 2	0.055180127154629	-1.08656202175505	0.0170138023779642
**Clu**	-1.85142863907122	7.93722692895868e-07	-1.80135108069025	0.00135355811851032	-1.73675668750882	0.0038756120838036	-0.211315489927331	0.762446551582433	-0.171966767312737	0.809448648971289	-0.0706701293615547	0.91907342846232	-0.775635601407756	0.11707343204115 4	-0.71820158161435	0.20274518980857	-0.852991422344193	0.0970206894864076
**Dstn**	-1.24524214370051	2.69137790727861e-06	-1.18889889984057	0.00163896071159957	-1.23575639680758	0.00108462323484265	-0.492107732599208	0.207235520821913	-0.451755670006397	0.280206009981891	-0.457005565319638	0.226045191352898	-0.775218790970835	0.0167743705634141	-0.713611201455217	0.0687468360448994	-0.753372168607565	0.0364372790582743
**Cxcl16**	-1.77619101999798	6.18829458845034e-06	-1.72065540391675	0.00117888487680253	-1.76438577074757	0.000721663101377042	-0.11423173131354	0.887910258786265	-0.0818543080064959	0.90636616928201	-0.282723832220931	0.608816829301938	-0.545879079891899	0.340119232930083	-0.484813975860668	0.362106521182443	-0.625279669000208	0.170046469998783
**Ccrl2**	-2.08739882777317	3.62948634465763e-06	-2.02183787080519	0.00211842147930892	-2.38721180080721	0.000704914258943287	-1.62705881175362	0.00454006003337134	-1.58672193994435	0.039014260345439	-1.72943101058974	0.0194630727478607	0.00765276313296249	0.992513445153508	0.072337864437306	0.935186721660103	0.00189532256657933	0.996587566584231
**Prr5l**	-1.69382973012088	1.30004452095092e-06	-1.63437979514174	0.00124922700188721	-1.5955692306847	0.00364658445293795	-0.0696551973805722	0.921863275869943	-0.028342844903633	0.969401916312636	0.0185903264004597	0.975852549036834	-0.300433972433551	0.579289558186422	-0.238211813890105	0.658740299336241	-0.192010184344385	0.674701129562028
**Shprh**	1.37151017461958	3.36449995852174e-06	1.42670340691468	0.00137017944603946	1.43478609777421	0.001346420670796	1.09857384437652	0.00361141890765094	1.13935978517914	0.0209374973754576	1.18558195518005	0.0238501918220 23	1.05324420431201	0.0024489374796946	1.1126721377417	0.0170300090775159	1.14688621804247	0.0165632891733874
**Aldh1a2**	-1.83362211061599	3.90112658020642e-07	-1.78069148243946	0.00243852272536463	-1.72990727292301	0.00694406330032287	0.0854433465160505	0.926859120729869	0.116576036403401	0.880068359846076	0.0302432164156656	0.968840577321452	-0.650031513419781	0.308131310986979	-0.594146768628053	0.32869281860131	-0.709843715990004	0.203827536214766
**Slc49a4**	-1.29984105452178	6.70110449469487e-06	-1.24434852240641	0.00184195227249874	-1.33800098483272	0.000588939093220215	-1.13381976709767	0.0018132583611707	-1.09156251903054	0.0223260304076448	-1.12542494794741	0.014091850342075	-0.650640113671566	0.0765916470057401	-0.58739052263864	0.152086836763429	-0.642214244576172	0.0553368139711203
**Cpne2**	-1.76311131444746	8.37745361583231e-07	-1.71126003944078	0.00181227902899556	-1.6688215661267	0.00545969688150763	-0.0140039651045223	0.985262847105204	0.0214574976171574	0.979798138303763	0.0942117426308793	0.889520792164197	-0.545409054720241	0.28277827858294 5	-0.490031315557678	0.391700396394012	-0.478733244838923	0.34878527481973
**Snhg20**	1.38890513068665	4.82116415142783e-06	1.44696239478805	0.00107647358237651	1.4020923692693	0.00162279464103504	0.264174575669919	0.620279035357305	0.309054331864091	0.535446026774889	0.326887803041583	0.579333632340654	1.21294628985724	0.000496021727002311	1.27715468806698	0.00827183148337801	1.24860185349092	0.00932106195007179
**Lad1**	-1.5750156319801	1.0523992724245e-06	-1.5245486448617	0.00206634289276405	-1.52824968720625	0.0038756120838036	0.448459276351929	0.377967079016733	0.481622725213986	0.375733700308024	0.434120541416615	0.366235215688281	-0.350161038434613	0.48128187332751 6	-0.29112637226755 2	0.600564610774447	-0.382422814713496	0.414648326399962
**Socs3**	-2.13065409521888	5.36340379916864e-06	-2.06387446480641	0.00113119179910181	-2.12583023541183	0.0017205466628859	-0.857233206944848	0.214716909730821	-0.818164957995642	0.213482723575583	-1.14107444697526	0.0823365485578746	-0.225605261986976	0.788174794072575	-0.153874015378138	0.839142812081786	-0.324056126959372	0.561522422627099
**Ets2**	-1.12501251488422	4.36623120049465e-06	-1.06367634478366	0.00187038484721339	-1.08435811296477	0.00152464081471051	-1.91198975194451	2.27699728810561e-12	-1.85861831618135	0.000286004448199555	-1.84039399328265	0.000841505679690564	-0.722309264828859	0.0157051797304704	-0.65329150319695 2	0.0671566317648455	-0.715593013827502	0.036217965712668
**Dnase1l3**	-1.30504779110799	3.98740324243122e-06	-1.24732675278689	0.00395547337998526	-1.36827357666167	0.000940220910900205	-0.609663542753404	0.127767408961453	-0.570191220693714	0.241701032270747	-0.609185623534079	0.150780489678442	-0.130961490801366	0.798045449466214	-0.0679919233791643	0.908273018268411	-0.153596445141292	0.7131140032146
**Chn2**	-1.16455344730175	6.93589085664228e-06	-1.10235186225446	0.00197241957785018	-1.16426675196131	0.00112123438837546	-0.500921185460925	0.179196809059106	-0.454075187881928	0.252454025697032	-0.482220990517084	0.18186160000035	-0.486547415864614	0.153891626057441	-0.417695897994946	0.259065711429369	-0.530730263380892	0.0913969418122202
**ENSMUSG00000081662**	-1.14201686886992	5.6962608454878e-06	-1.08322648495552	0.00156206554556275	-1.12675498787609	0.00193404279853685	-0.469753976874967	0.20155865326827	-0.41862108428022	0.267299263442749	-0.402021902899936	0.289905913716464	-0.75750010504163	0.0127735471883985	-0.6875358472646	0.0536625197927594	-0.740528828166109	0.0389311267734193
**Flt3**	-1.47445019825237	2.54409822713939e-06	-1.4216528241683	0.0036410245232746	-1.43635081598556	0.00206724368246457	-0.368913845997316	0.473742903294338	-0.329825981657319	0.575925923030882	-0.286707896855647	0.567647034096147	-0.752059683001244	0.0615481454074416	-0.694233140070019	0.175026609105636	-0.783206998497904	0.0729286625340911
**Dnah8**	1.17435980014591	6.20078163332478e-06	1.22700601388702	0.00219248312526658	1.19298211579771	0.00143374070072076	0.842862621044039	0.0129102477438987	0.880797699947661	0.0444686174221149	0.843440365054633	0.0490876131822416	0.706118100348332	0.0292177937609382	0.76925150548883 4	0.0618777198305	0.66677959822058 5	0.0817758944464728
**Cdh1**	-1.89307901369229	1.12280713178514e-06	-1.84543898724436	0.00136357723311314	-1.72590067777904	0.0134418193362435	-0.192193694302484	0.794794501768118	-0.159429889437674	0.829732936141518	-0.131230014116858	0.870041976458184	-0.819489043200123	0.10709001005315 3	-0.762630162083418	0.185804092971603	-0.74399799984874	0.214983670686702
**Rnf125**	-1.7836408532043	1.15382779735599e-05	-1.72574437937655	0.00218201162465416	-1.90297928169142	0.000834087838250891	-1.32169819364908	0.0116370967172272	-1.27266405012081	0.0502036096183526	-1.43195284032029	0.0234760364074715	-2.3307605636696	1.27889799431602e-07	-2.259637241188	0.00258396353296751	-2.31661846660803	0.00213205519032263
**Arpin**	-1.52034049647789	3.8163488719702e-06	-1.46701399938734	0.00150762885552655	-1.50327346108853	0.00375444813645615	0.166713521689407	0.785681263082712	0.203792278420094	0.717806257117443	0.142888290269212	0.796781362798341	-0.0862094839836976	0.887524575563495	-0.0251558909444779	0.968019233844017	-0.143284311949845	0.762306918554614
**Sik1**	-1.75981165891209	2.99939647716769e-05	-1.70249371106807	0.00217644064491383	-1.87579560747713	0.000350009228507763	-2.21738058448703	7.77373469971651e-06	-2.1754201517354	0.00474125927919992	-2.25035641853732	0.00135343510499697	-0.771134729959868	0.171289452090525	-0.705913195412151	0.210824740455987	-0.831713372380678	0.0769867572483751
**Il2rb**	-	2.083123307	-	0.001593180	-	0.000836920	-	0.455552498295	-	0.45862212004	-	0.39489609514	-	0.2106648484063	-	0.243694502445	-	0.084162379789

	1.59006197950493	01515e-05	1.52588812543389	15415706	1.56435054857272	215495676	0.451131068025166	184	0.402150833999559	7373	0.426748282515643	2742	0.638327920768065	73	0.567149606693844	198	0.772628764397814	7452
**Dgcr8**	1.03086872833184	1.64175991416329e-05	1.08295365010783	0.00136246849480111	1.07310180747373	0.00132754088610033	1.08540560764481	0.000168034901561783	1.12170221019127	0.00577631018880274	1.00980137895703	0.0106759039701533	0.934725578157486	0.00068048092254543	0.995346686253369	0.00827183148337801	0.980172132771518	0.00757323000038996
**Ahr**	-1.63484836275487	1.10381853493287e-05	-1.5693624403804	0.00217884891503066	-1.61508669178517	0.00136485722713076	-0.941196763397549	0.0607050094922119	-0.888448432207995	0.120372032574454	-0.912024550948266	0.076550557764864	-1.49114961513536	0.000440449367991939	-1.41952111704413	0.0132910947540386	-1.64811645728197	0.00483375084872292
**Mmp25**	-1.81749112315599	2.11038368034081e-06	-1.76390762462517	0.00171550726532126	-1.64390216829204	0.00969222646313837	-0.230800177265979	0.742493089539858	-0.199519002650067	0.777170518135349	-0.129697003251528	0.85916872172075	-1.06787135239055	0.0232606451992233	-1.01145063171268	0.0798967804542738	-0.979607950784064	0.089856001924514
**ENSMUSG00000086825**	1.75000714219868	3.17243253332242e-06	1.8063032852784	0.00261289301779279	1.80966734673423	0.00438162724909791	1.25370254946036	0.0103575490247985	1.29743942062332	0.0455992394064165	1.34757257014967	0.0683920615819224	1.95598767634191	2.14872094536016e-06	2.0157330263937	0.00485982028158782	1.92237691639803	0.00700658075677624
**Plk2**	-2.65228971219202	1.7015543660722e-05	-2.58600607571606	0.00172769696207115	-2.76492792674025	0.00124559256032476	-1.73657597197271	0.0348987509593051	-1.71437218529466	0.0615188732911837	-1.93656239613886	0.0234654233971623	-0.47082416660686	0.649068278695438	-0.407669484440184	0.64808854207308	-0.497915841797515	0.362771920897365
**9930111J21Rik2**	1.4057592968374	5.58670735563499e-06	1.45796206856746	0.0030998904182555	1.46102627802023	0.00216341154077216	0.801256487034823	0.0578911697660336	0.837599345391645	0.109152629077672	0.681016959477122	0.216124223775514	0.743591766421668	0.060398460714803	0.803914195142337	0.107189360859804	0.696734598253641	0.156599894818302
**Fscn1**	-1.8836220803581	3.75127542397207e-05	-1.82716814865276	0.00217912321210125	-1.86331917803814	0.00050113363554286	0.0402196030587216	0.965275712465365	0.0757817841938705	0.927994134120108	0.0178444834318112	0.979652245716782	-0.358377773797253	0.629851266217818	-0.295949851632587	0.657072824726129	-0.481729089146827	0.33479957121105
**Anxa3**	-1.88676270779138	2.22837755401141e-06	-1.83363488357796	0.00295127073303588	-1.79902812236588	0.0070957663265185	-0.185289098290879	0.807870898804764	-0.150839996490206	0.853909268584883	-0.278558089599658	0.686394628539068	-0.412448068367481	0.503284686763068	-0.354347977725532	0.609789556806109	-0.571564718877632	0.302031470812064
**Cd63**	-1.56956929233509	8.63735102285375e-06	-1.5142451493483	0.00207579515358269	-1.52295826775347	0.00281297640167746	-0.563469867725182	0.283363855147402	-0.522845485649336	0.34919899619216	-0.562840486329442	0.264180334385923	-0.432193980077867	0.39988888065964	-0.370498518511168	0.490674923727475	-0.602741616153926	0.15507846216748
**Zmynd15**	-1.84307593257912	2.953238983393e-06	-1.78417174409973	0.00346631037364173	-1.81208680070664	0.00499915475003416	-0.0206794819794312	0.980345573761504	0.011542415515326	0.98986558820229	-0.220533178136633	0.740743485033994	-0.4698374529597	0.424153171987065	-0.410018817529181	0.544832933099507	-0.651775037296269	0.221750550387287
**Ctla2b**	-2.62818692176118	6.93589085664228e-06	-2.55632701861708	0.00234953798098262	-2.8854911552388	0.0034074259573505	-0.615018547430693	0.51196108706591	-0.573202552313396	0.543970344326632	-0.719666766737897	0.336158133087765	-0.667792166798283	0.431694500180438	-0.597272950813931	0.4828956327382	-0.848641934334701	0.197708097327137
**Tmem176a**	-1.35524254463765	8.82037944514881e-06	-1.29778380306661	0.00255905198292606	-1.31795869666424	0.00293605685837103	0.285526053932046	0.577297887937366	0.319602906981146	0.528941054623109	0.140887702445853	0.774302495514571	-0.326705969984048	0.481061487269614	-0.264581097672314	0.59282321476865	-0.428168256868492	0.270286499754698
**Gm4874**	-1.26221089988947	1.32108503340771e-05	-1.19966168787624	0.00184984101139331	-1.22656147357057	0.00274395936574862	-0.109152787242764	0.843437014499715	-0.0589810591754663	0.913305556953846	-0.0943126993289235	0.84966619402506	-1.02169877015189	0.00236886741439478	-0.949829788449467	0.0220914558502971	-0.998630197926727	0.0195638117092137
**Jun**	-1.26175954296918	3.87191466855149e-05	-1.20176148913643	0.00187430824707099	-1.21979998514292	0.000940220910900205	-0.063689207229069	0.900031219267568	-0.0217144577935003	0.97071028334936	-0.00690033465303682	0.988272035657099	0.466004236442376	0.152930189563561	0.529928392980032	0.177688401270921	0.544164512235419	0.115744969336681
**Nudt17**	-1.62290896140941	6.17083169908461e-06	-1.55922452998705	0.0022888454350398	-1.62327961904599	0.00500444759591743	0.756202287381817	0.127951011964627	0.792413991219768	0.146373333737761	0.58179036524756	0.203199260016617	-0.305451268446855	0.591643742260861	-0.241896807276118	0.686409777525536	-0.40584829137371	0.40371989495993
**Mreg**	-1.28725191302766	1.12416779261242e-05	-1.22799184568344	0.00275159470979964	-1.29472253640057	0.00232843171292822	0.152569370513706	0.780132856837469	0.188632728520202	0.714591022849921	0.0157704962499904	0.975870487484014	-0.0557191863970842	0.919526676565849	0.00804572169538474	0.990101350400389	-0.136469679164258	0.74087696321767
**Pdzd4**	-1.61247250902997	6.66748214189132e-06	-1.55811987966728	0.00211842147930892	-1.57614617004498	0.00558413512373769	0.17823256599177	0.790511459848783	0.215192380099115	0.732617462130143	0.100090767304043	0.874494532854801	-0.925950067016975	0.0369499434122657	-0.865890753894125	0.0995281379800046	-0.940741403552505	0.0723217050408768
**Ccl22**	-1.85129755486958	3.17579462753629e-05	-1.79571355051665	0.00167185903479853	-1.82035026756502	0.00163761802092519	0.357357374641475	0.642156304291091	0.389794014528025	0.54315902186236	0.271805953077172	0.628983998026957	-0.678286793651202	0.276936211769394	-0.618705161122678	0.280531174447999	-0.790698991910862	0.108564208468796
**Glrx**	-1.10791847174982	2.14135739852354e-05	-1.04705341066219	0.0021980473741783	-1.10398304162108	0.00203183510198348	-0.210391715323036	0.636415425787099	-0.164186959913513	0.704929024659364	-0.271215065415383	0.467267559686081	-0.49189886872183	0.150395185729521	-0.422338909206134	0.238429898393884	-0.544825364521388	0.0963909668169736
**Peli2**	-2.18202282577111	1.69851552955042e-05	-2.11534024077481	0.00290473944212945	-2.27605109808069	0.00229999798182933	-0.482939159458114	0.5615786225989	-0.450646026025318	0.587738617699647	-0.733352539059008	0.236467937403551	-1.15192835360047	0.0683065644141678	-1.0860063993417	0.122247145458837	-1.31587499770132	0.0357026841644685
**Atp2b4**	-1.07322225014986	1.97718135473612e-05	-1.01724564973021	0.00284031954605135	-1.07916607940832	0.00202393499193686	0.277677549607409	0.495939948470728	0.317108235474371	0.421177691185687	0.266173409365645	0.431728743266992	-0.443067327739821	0.188663659148466	-0.38038578936759	0.292201076464056	-0.474282851726081	0.134352547736943
**Ifrd1**	-1.0696872412899	2.10464974729376e-05	-1.01201886908646	0.0021799067033093	-1.0485788482028	0.0025003524474261	-1.17533817165693	0.000110150327722219	-1.12929843851565	0.00762387399151509	-1.13787789766234	0.0126370923564494	-0.439203249050984	0.191190130949947	-0.371777912664654	0.285349489195283	-0.447525524699676	0.156803355399406
**Tbc1d4**	-1.71653930344325	3.4440334773131e-05	-1.66502422746435	0.00205190311393164	-1.60561959553026	0.00163761802092519	-0.534218880718296	0.414664031356781	-0.502612103634593	0.40552861756916	-0.490670819570788	0.349050433664693	-0.694866499032483	0.220576790915149	-0.63636853037094	0.247808167981978	-0.699064011611637	0.132315454360187
**Cd209a**	-1.29899088091639	2.63831104032938e-05	-1.24294192286401	0.00261289301779279	-1.30393651473527	0.0017205466628859	-0.410042907357917	0.393472668186691	-0.367929755589879	0.449654059237431	-0.425332954695587	0.305525055225064	-1.14703595023677	0.00118508872565309	-1.08373694451565	0.0181778281296368	-1.16486162980475	0.00932106195007179
**Exog**	1.59567807716233	1.24421427482108e-05	1.65054659880812	0.00187430824707099	1.58853844293879	0.0053435568765641	NA	NA	NA	NA	NA	NA	NA	NA	NA	NA	NA	NA
**Aif1**	-1.33814262119814	2.99939647716769e-05	-1.27928222922263	0.00285078260013578	-1.40035425998821	0.00152815364729295	0.0185216050190389	0.977767823770427	0.0590629240493295	0.926886157258045	-0.0696435104526532	0.889389327764938	-0.344807744542245	0.46947082704023	-0.279153222304374	0.569614182470303	-0.365143148505685	0.317337343305714
**Grasp**	-1.68732371076755	3.60911173397598e-05	-1.62769101476664	0.00354964726950426	-1.68188331483936	0.00117096975565783	-1.37512953238294	0.00823374351238228	-1.32535452323487	0.0453873477225682	-1.55018166988499	0.0164529568261361	-0.758489931221182	0.153437777522347	-0.693447800115415	0.23418087886016	-0.754814674137243	0.0814334544216266
**Tjp2**	-1.23591205701206	2.14353812537484e-05	-1.17911298097494	0.00265063091090716	-1.22376258902106	0.00293698898596676	-0.0939748432878547	0.867731669064662	-0.0530744410437106	0.927891738995661	-0.130410393820482	0.782534724943523	-0.522599756315471	0.169603127824332	-0.459085606906	0.264382549047351	-0.516352099581721	0.149380949140498
**Tmem176b**	-1.22736780077574	2.14353812537484e-05	-1.17078954565397	0.00418066824847906	-1.24270144716415	0.00205405231000214	0.0884850342618267	0.879633769615985	0.123858467646772	0.822161948532423	-0.0319021232799354	0.951425712399066	-0.430525553479768	0.289654870814163	-0.367665594109369	0.41091188634818	-0.517204670688598	0.158666733938561
**Klre1**	-1.82348707995824	1.43972811366174e-05	-1.7643165449859	0.00255268041843989	-1.86501665615806	0.00565342418191241	-0.678892850330987	0.269672721811295	-0.637858732363861	0.32568558374439	-0.643098596660143	0.305100887073854	-0.0477488154335063	0.955676884840282	0.0147553540289955	0.986014805717597	-0.137417356890342	0.812590385367088

**Sh3bp4**	-1.53381780729487	1.45209120896102e-05	-1.47630470427199	0.00325776490998035	-1.51802980708581	0.00499915475003416	0.216196988506869	0.735497358785061	0.250843951590325	0.684833130062833	0.151192082858025	0.797134496649282	-0.434765275915816	0.398012336649033	-0.374818466809492	0.501830891714966	-0.481965587149596	0.298441241706697
**Alox5ap**	-1.31743202895972	2.18743153699527e-05	-1.26187055282645	0.00514197187644287	-1.32155812077499	0.0022298312054306	-0.718583995573194	0.092144585466518	-0.677344397224432	0.18434858758861	-0.776567048012455	0.0987491269007575	-1.10786210915218	0.00218942220111783	-1.04747319337609	0.035763726773699	-1.05100040764322	0.0195638117092137
**Ccl3**	-2.6754152041637	2.2064549496191e-05	-2.61039075556377	0.00204105089758268	-2.63728767906314	0.00578854227501271	-1.57176447837669	0.0670917011803252	-1.51223929504922	0.0995399521704522	-1.52711566154221	0.102699865572565	-0.226347654373962	0.844326618907732	-0.158548362126414	0.882917825541877	-0.179553196991967	0.801369694955509
**Epha2**	-2.27078443165812	1.28144481187009e-05	-2.21938962017406	0.00232538871458567	-2.16749243578353	0.00926490096969248	-1.29199358729704	0.0639061807036538	-1.25061203423245	0.113060236216745	-1.33087679319803	0.117012165364211	NA	NA	NA	NA	NA	NA
**Gm12185**	1.32101473216093	2.72403163842649e-05	1.37470085642779	0.00295127073303588	1.34726589594753	0.00358714819282462	0.537926398516273	0.247353316921475	0.577157697532882	0.249504276173592	0.517359132600626	0.369717202952474	0.962133375564967	0.0106576788820831	1.02139684018396	0.0370692493405793	0.848563072814523	0.0822208868152441
**F11r**	-1.53521326242558	3.19539628192919e-05	-1.47352387094361	0.00382223592087131	-1.5540365532752	0.00275303836835099	-0.498031429125598	0.372544761927829	-0.453011473442345	0.417254596378502	-0.519535355443013	0.281795485314553	-0.521074529090904	0.297912152477596	-0.453330287414775	0.360699897983746	-0.592655198282497	0.150340518707004
**Rbpms**	-1.5097932671917	2.60630925590689e-05	-1.44636080697118	0.00296878684597315	-1.50656131158629	0.00455102263821353	0.269701604504502	0.654234746070102	0.31421024645002	0.574515217791693	0.221905719571198	0.656295800615398	-0.455521784918499	0.365077577103254	-0.388753477076423	0.435983068835044	-0.522963233050055	0.234689536165079
**Tlr6**	1.08269213901151	5.87966460901183e-05	1.13746780088313	0.00206634289276405	1.10999501305454	0.00311497838904992	0.444799321178697	0.266357374465813	0.486904427831492	0.222579948451672	0.424878997134478	0.355230633848285	0.965369980119837	0.00172238830979833	1.02894238589923	0.0114102259499935	0.930956709795655	0.0201677547788638
**Clec12a**	-1.16145236026489	3.15258376141123e-05	-1.10627281747247	0.00398666810412281	-1.14329964804456	0.00311198039752625	-0.251513293660304	0.594156558438067	-0.21022372139256	0.664674206436835	-0.288563786143534	0.495409195969484	-1.0621160551691	0.000940420929762346	-0.999682718287673	0.0203730255132842	-1.05607363102076	0.0129741150964912
**Marcksl1**	-1.05882704881299	1.66083965077449e-05	-1.00501676673332	0.00615416096254756	-1.02082786582148	0.00394089628358324	0.155472322082329	0.729955821962158	0.192030675907299	0.677466717530847	0.115758788509037	0.815579938816072	0.0377682952017225	0.939296469819964	0.100217714189022	0.835823920792553	0.0142776094566142	0.973413047387774
**Man1c1**	-1.33310175688378	3.17579462753629e-05	-1.27454062025905	0.00325776490998035	-1.31017176242435	0.00401025077591437	0.264647328779142	0.626862254548429	0.308696283340429	0.553463607240946	0.268235346084166	0.546923091268502	-0.743108618160266	0.0648924344252302	-0.677626006179045	0.135347709986024	-0.831773351966283	0.0569111957183086
**Gnb4**	-1.39337090213131	3.95521918748889e-05	-1.33658006752698	0.00340591334551374	-1.40786953060741	0.00320639335557757	-0.222799025499584	0.711607085921891	-0.18477425383107	0.754532504410938	-0.286897293832851	0.555280103600107	-0.328981199239198	0.526302555980687	-0.265595434266797	0.616471470129926	-0.421807576296841	0.298299725917708
**Jaml**	-1.18424760305411	3.34029445033168e-05	-1.12977592974611	0.00332084383501411	-1.15207873104151	0.00395896501399789	-0.195081091382054	0.701304337352514	-0.156683537291122	0.75395974449398	-0.223362618049933	0.618940361575321	-0.771343418122818	0.026076455241614	-0.709158441715749	0.0835829978907259	-0.803141361461607	0.0442745738997502
**Havcr1**	-1.15918189905985	2.61561205717858e-05	-1.10729086317623	0.00263927322751753	-1.08043353689635	0.0074734688490224	0.376585430787368	0.374931778835199	0.413733602314361	0.317141366414665	0.396106460643589	0.305525055225064	-0.981450532933789	0.00207728862496727	-0.921991603586504	0.022419514072983	-0.881290127810261	0.0382565087070335
**Cd55os**	2.29116577740787	2.36036055175749e-05	2.34239563978558	0.00354964726950426	2.55272565711756	0.00653096047053452	NA	NA	NA	NA	NA	NA	NA	NA	NA	NA	NA	NA
**Ttc39a**	-1.7599451315884	1.43972811366174e-05	-1.70570521084179	0.00363739861348277	-1.70635008266265	0.0134853286680636	0.117244212856187	0.885961896808331	0.148486764601571	0.850414969965809	-0.0547256026506003	0.945894624901144	-0.213685588985707	0.758810085670877	-0.154831578561668	0.837859652495916	-0.243678847075206	0.693877644530193
**Nrgn**	-1.20820714637606	5.14957720489714e-05	-1.14900130733715	0.00309670471340002	-1.1549236492481	0.00449593521535025	-0.521876989792412	0.226734807635319	-0.475140108460401	0.284318316765764	-0.518704934789732	0.235709170911849	-0.864113411024755	0.0150088604156968	-0.797080558293087	0.0566777340014509	-0.794845780765777	0.0464244327312094
**Gdf11**	1.03127626843934	8.68030800976175e-05	1.08895273995218	0.003544666878199	1.06284098735494	0.00237281806127253	0.421676934667872	0.280127656057462	0.47084549850274	0.249540876010025	0.458445899074397	0.272631393513047	0.560021138596118	0.0980723744743434	0.62265459922952	0.10649280439964	0.614492298283775	0.089856001924514
**Basp1**	-1.59160387098353	9.3969246288363e-05	-1.54069844667255	0.00275159470979964	-1.52907480409678	0.00286634924444731	0.0702134087897263	0.933922652323785	0.103005407073277	0.880676736654694	0.0184132923185071	0.978518127743334	-0.436053770853083	0.478642028488196	-0.377523814297351	0.503439073549538	-0.493616699999853	0.302013639382116
**Cadm1**	-1.24070893702558	7.12512096963784e-05	-1.18561492167039	0.00490107685421013	-1.26658382788624	0.00222952813378759	-0.416280303270865	0.390444274486351	-0.376117756507533	0.458503935200679	-0.384846716790736	0.363302344080049	-0.670878355874975	0.0924839183936005	-0.60899358406902	0.176398081777934	-0.720714061418853	0.0617067322949439
**Fmnl2**	-1.58352725212695	1.37458225897127e-05	-1.53352861024151	0.00587800648527032	-1.46784839892928	0.00978452699190465	-0.415863637048301	0.482080957063414	-0.381628808715602	0.575831444467327	-0.313786943086123	0.620405819917556	-0.3379590413719	0.555740444196994	-0.28215569142976	0.671351589770406	-0.324682531842323	0.544938927636791
**Kmt2a**	1.03953423547998	4.02409369403911e-05	1.09686877264141	0.0051714889575987	1.04348195019871	0.00411432934893085	0.784734023430121	0.0173901962510572	0.826303316310444	0.0571795191149631	0.727493036168844	0.0826954907028555	0.798791630474976	0.00840211423803725	0.862657664583215	0.0403084041672386	0.775648087866028	0.0469385663120546
**Ptpn22**	-1.12112616651663	2.80921005928112e-05	-1.06078249127469	0.00703440400449493	-1.0883619207186	0.00466876576899879	-0.962232313849638	0.00466323171995158	-0.917870929647339	0.0505503225477341	-0.908763499938474	0.0473790090525925	-0.845594464270704	0.00831154601951255	-0.776847622245977	0.0709883238946723	-0.807857730408797	0.0484151558843864
**Mpeg1**	-1.27103717559599	0.000210092987527463	-1.21767982386381	0.0037976434133112	-1.27191114074304	0.00118165965951825	-0.556173770196824	0.270008144466693	-0.513103956114597	0.280712877629542	-0.575618431397495	0.172084606663463	-0.722459562252759	0.100898666432856	-0.661190325865072	0.134875481433061	-0.69600810600504	0.0737475984108781
**Zfp7**	1.01788291107748	8.91503328740858e-05	1.07858461610454	0.00207579515358269	1.00750219937387	0.00514812012687161	1.18564196734585	0.000115191682243726	1.2302644006224	0.00461070053916201	1.15579243491057	0.0086563007370959	0.840763452435116	0.00544092400358014	0.903347228503049	0.0165876177761412	0.84517033108213	0.0300804913419632
**Cd209d**	-2.15139625519299	7.01565981994355e-05	-2.08579978802505	0.00482345632209665	-2.39099112462584	0.00314096655878796	NA	NA	NA	NA	NA	NA	NA	NA	NA	NA	NA	NA
**Slfn8**	1.3042977784908	8.85298307934745e-05	1.36254790145829	0.00295430356050291	1.29690934803974	0.00412608294378162	0.958343488714148	0.0270771575553155	0.995503293449812	0.0492177116017334	0.734373723081346	0.16701086745952	0.904841921123405	0.0239907372384571	0.968217771341896	0.0439339637346893	0.888379645294098	0.0657064228871846
**Sytl3**	-2.16103338328008	2.99939647716769e-05	-2.09062584335327	0.00349931303592157	-2.15461410851696	0.0105906980656213	-1.22966830142282	0.0784158375379513	-1.17681208701835	0.137208847554669	-1.2381950681079	0.136923187827533	-0.179443575229151	0.845546137447182	-0.108386539173622	0.907749703255411	-0.46276207429398	0.47675094147077
**Errfi1**	-1.56487519369967	0.000111922966269765	-1.50079456057228	0.00522798346138642	-1.57880329814466	0.00203183510198348	-1.2938194426863	0.0118452164724507	-1.25106002726163	0.0464186391837508	-1.49552981257248	0.0153070651912076	-0.905137671244485	0.0698182965340684	-0.8364332938192	0.124828699729812	-0.998549592233329	0.0324176802035087
**Bst2**	-1.19700665565819	6.2323169271621e-05	-1.14410683398348	0.00541193976449198	-1.18145316802725	0.00414455420739935	-0.356041840425141	0.455579381347335	-0.311561234926012	0.537325644758084	-0.392368803912139	0.38782689348201	-0.698988210934907	0.0623935820747896	-0.635107136611154	0.147711045986719	-0.752886018338752	0.0641360722042809
**Chil1**	-3.44002853631569	3.21655952616179e-05	-3.38570491405067	0.00346049404570618	-3.29606872819759	0.0128024147933158	NA	NA	NA	NA	NA	NA	NA	NA	NA	NA	NA	NA
**Vwa5a**	-1.11446544	5.66349569037802e-05	-1.05939209	0.00514197187644287	-1.07974929	0.00501753191818622	0.0110804788673371	0.985006164997718	0.0464003743446932	0.936947840121096	-0.03153225389	0.951438818809568	-0.35497140119694	0.371461944988643	-0.2940500721543	0.494196337864597	-0.3961111767685	0.278705021227158

	065082		476931		013236						01454				86			
**Atp1a3**	-1.13549131208792	7.45085108404256e-05	-1.07728011505048	0.00520421894221554	-1.12941743428074	0.00401025077591437	-0.146908927446897	0.780703449529234	-0.104999398050622	0.846139390971612	-0.085935737350359	0.864968612553886	-0.621704243152334	0.0882871917685269	-0.560431999060176	0.176188233591068	-0.568428684310727	0.121401982151965
**Insl6**	-1.12248182931686	9.90032072240516e-05	-1.06841822800331	0.00527272286959051	-1.10873019612343	0.00339599625783488	-0.605143303304488	0.124409449006988	-0.561428661917662	0.195569912330087	-0.661972769627991	0.103847965797458	-0.636593485383321	0.0745127460043034	-0.570916708494855	0.153155227614835	-0.66043204279618	0.0614206047571168
**Ppfia4**	-1.30922227637433	6.55966866964526e-05	-1.25497704902053	0.00425542011475559	-1.25552575224617	0.00646918103177725	-0.193032900933864	0.744735299108816	-0.153774780925323	0.792347203565788	-0.210455834967758	0.690292706507411	-0.88658923727059	0.0248456039839519	-0.826043287946336	0.0798967804542738	-0.852363110245713	0.0587415053206507
**Ccr9**	-1.36490688526709	5.14957720489714e-05	-1.31152211742728	0.00487328259229524	-1.3033274755181	0.0073454848577788	-0.364299599670659	0.503939372831146	-0.322739272802821	0.575342956140921	-0.312611728688553	0.561715068978803	-0.764540816250365	0.0718430034156681	-0.70305086092843	0.155247785486684	-0.729604503306702	0.110846619372179
**Gria3**	-1.91397768395972	3.18548577809399e-05	-1.86050398496637	0.00309670471340002	-1.81090077845748	0.019694160278593	0.174926374907631	0.838676136283814	0.207023975952791	0.792249673720355	0.102464719482142	0.901863690923 95	NA	NA	NA	NA	NA	NA
**Klrk1**	-1.60294764302594	2.53263262293687e-05	-1.54213691384251	0.00750448966101928	-1.54390616506878	0.0104065952551167	-0.260564284867909	0.760308591227024	-0.217334787552675	0.774119336551645	-0.241988236145367	0.725006980698915	-0.219086775595893	0.789398503917611	-0.154562631869975	0.839243227971946	-0.319611335987449	0.573006369848996
**Il12a**	1.27674830547824	0.00014357651826384	1.32913521313148	0.00324877130648564	1.27099291758141	0.00501753191818622	0.358475124483686	0.509951393966219	0.394890128843005	0.449446091481 21	0.175794484834808	0.808409586468872	1.03378288450279	0.00840211423803725	1.0955508045812	0.0236276325757296	0.998319950155962	0.0395355847826052
**ENSMUSG00000087060**	-1.74332902396233	0.000120808319020751	-1.68043008304878	0.00514197187644287	-1.75877223282442	0.00378011920431213	NA	NA	NA	NA	NA	NA	NA	NA	NA	NA	NA	NA
**Ccl4**	-2.37915874507638	3.74883454556456e-05	-2.31116439587428	0.00504450083405178	-2.47504194718106	0.0126767356387299	-1.01204680988916	0.222482612831271	-0.978310818790261	0.291813719569158	-1.34333702975512	0.144202619191458	-0.0966201593651873	0.930682036258848	-0.0292562151821403	0.979234500104492	-0.195189237675649	0.802527223384798
**Prnp**	-1.48190933866472	0.000126782590809089	-1.42253924632117	0.00493244186888045	-1.45550656210059	0.00408743546447173	-1.01749145825201	0.0452951688697263	-0.977377887210566	0.0960313624474263	-1.14458736812834	0.0480780987414121	-0.63990345174112	0.21089460503523 5	-0.573582359988975	0.290178135321102	-0.794601218702594	0.0825338649139974
**Chdh**	-1.51864601207252	4.99312800426512e-05	-1.46646822616388	0.00462145430836201	-1.41970571191165	0.0111494415211245	0.0577659639119471	0.940209392210481	0.0944100037941211	0.8975774558252	0.124596862600827	0.849666194025 06	-0.959264460890495	0.0365608681889293	-0.90006991020489	0.102610021797838	-1.0942668485994	0.0506041596492442
**Pik3r6**	-1.40316542976832	0.000121644437474757	-1.34427935499687	0.00558133422325328	-1.42039812424074	0.00408743546447173	-0.471140902959252	0.392597186301836	-0.424221902237414	0.425265956466902	-0.563752021460426	0.235967097879186	NA	NA	NA	NA	NA	NA
**Sertad1**	-1.20095640881262	8.39316321654647e-05	-1.13195026687713	0.00435895174232881	-1.1613536486708	0.00785948118789451	-0.746216196435616	0.0684458421292109	-0.696256228544287	0.125908935904475	-0.680098513244802	0.158077180162688	0.0690983002908074	0.901400202543181	0.139287432177618	0.78068017416499	0.150554657393856	0.716149272904848
**Tbc1d8**	-1.55333520729723	0.000141805703052761	-1.50024574069045	0.00401820414791866	-1.43477065455672	0.00526209763415555	0.13174832418734	0.869205505522859	0.164367238470827	0.809883493157942	0.223522185703268	0.710175571630472	-0.285196980608254	0.67089474497615	-0.228672628152527	0.714201310743127	-0.213395936961178	0.693146985112247
**D16Ertd472e**	-1.13555155551914	0.000126782590809089	-1.07205313032126	0.00633481173146984	-1.10538805262778	0.00375444813645615	-0.964566916901226	0.0105163257387134	-0.908693970844677	0.0518909237094134	-0.937757471037953	0.0457950583713886	-0.885751664343884	0.0115407017575491	-0.81482750392406	0.059592842306098	-0.90801479547687	0.0233589398216083
**Cd28**	-1.29380376080163	8.4035640369639e-05	-1.22798961023467	0.00561907306444238	-1.24847190547436	0.00708912940620372	-0.25887134584848	0.643918682023809	-0.212740321420579	0.711136278963557	-0.349507146700466	0.485791859554304	-0.744865260287229	0.0712921695897116	-0.673791333528621	0.155247785486684	-0.837028825996839	0.066575546495337
**Grm8**	-2.14103870134285	3.95521918748889e-05	-2.08724435242047	0.00284031954605135	-1.88013450740079	0.0310517662487404	0.349339259793682	0.693429823489684	0.386057524106545	0.619117225818656	0.618598313600893	0.373637374625779	NA	NA	NA	NA	NA	NA
**Rasl11b**	-2.723050656815	0.00016143923309813	-2.66117553055087	0.00632071768223431	-2.76581713134926	0.00441771960340657	-3.43201999212104	9.18857173518053e-05	-3.33325486182485	0.00962647731921708	-4.87833571063209	0.000747999146219181	-1.39963335929326	0.122072557662791	-1.32930471763167	0.14664648744954	-1.41211824423975	0.0941961014731531
**Zbtb10**	-1.15001682819343	0.000161297267250464	-1.08836894394965	0.00577728985078937	-1.14752084744265	0.00501999572478232	-1.4352991883139	7.56766109580546e-05	-1.39057319091246	0.00785230665453996	-1.42652325229517	0.0127170408990398	-0.101211532363714	0.85237394384214 2	-0.0354906223296521	0.952360547175145	-0.16962219369711 4	0.673392134262014
**Fcer1g**	-1.31184403752085	0.000149484801014334	-1.25031204486732	0.00783256326489212	-1.34362730893801	0.00419335882178217	-0.719777231275224	0.130891084621383	-0.673649157203163	0.216476035984529	-0.733425516604078	0.150424061522224	-0.42667004764232	0.38800482762472	-0.362528949906293	0.505043258251153	-0.466423369809146	0.27004433472911
**Vegfa**	-2.33444967157322	6.49948767175238e-05	-2.27338375616544	0.00673473647145142	-2.3149850037465	0.0124649117334807	-2.72776206400562	0.000118944475538685	-2.64142316211788	0.0127458978530409	-3.88475322907676	0.0061363371560941	NA	NA	NA	NA	NA	NA
**Phactr2**	-1.15437957460967	0.000133461996010216	-1.09961885138697	0.00545239481192287	-1.10247639498237	0.00779042952823354	-0.0279906747682714	0.963047650141207	0.0122759312855126	0.985153434641876	0.0438016005554781	0.935299324144962	-0.364458436470346	0.398429290167473	-0.303335232717508	0.504381652656792	-0.366380883836489	0.342403061684241
**Cacnb3**	-1.65937256568807	0.00029621251751632	-1.60547703255259	0.0051714889575987	-1.59363343122311	0.00402770925271045	0.391028222562627	0.611127364911636	0.421317075444996	0.533205116765407	0.269977687620775	0.673055080887 09	-0.00286870910477533	0.99665620569104 2	0.0572572030258789	0.942722634182097	-0.0668095763919172	0.917334814211646
**Ctla4**	-1.8364602183621	0.00069148728508973	-1.77856421690835	0.00583382407449106	-1.8728641940038	0.00180178138103099	-1.15710909976258	0.110018361551245	-1.12308883449727	0.129305995317972	-1.5438178156825	0.0264508898207444	-1.11762093980952	0.100898666432856	-1.04941498589006	0.125543857093319	-1.32430252068005	0.021583289835285
**Osbpl3**	-1.25742643852196	0.000107074188470241	-1.20081507633969	0.0055235554898882	-1.18914417407659	0.0129668242502307	0.424710480074711	0.393472668186691	0.463810581684352	0.352298845868987	0.447776012219443	0.322561095815792	-0.287248949394382	0.567307225625876	-0.2238998004466	0.669701381007166	-0.361004375133459	0.429206458952655
**Frmd4a**	-1.4196042978526	6.45900926847945e-05	-1.36722274352737	0.00717170348421043	-1.29549924844972	0.0168300021515279	-0.306394343703712	0.608689991482822	-0.26955584189945	0.677505770486864	-0.158730918608801	0.821398598794351	-0.690950691571118	0.136702819461572	-0.633330609090427	0.247784176231037	-0.65276533126264	0.205461633442695
**Lmna**	-1.3268329924539	0.000238111621259883	-1.2668536167369	0.00698237592404128	-1.3344771164832	0.00472543183163961	-0.233133066740649	0.710251746692709	-0.186387117753347	0.758937745638554	-0.211431444410439	0.685813394563 13	-1.07309195676351	0.010420706322597	-1.00550168620894	0.0437337107087052	-1.0349679786058	0.0278528110113312
**Olfm1**	-1.66367662013805	0.000289484462926413	-1.60398141470295	0.00854925918926377	-1.75766746353366	0.00414489884399951	-0.514508661948663	0.476580906119356	-0.461533778362368	0.541435083095913	-0.589312266788297	0.340634861837551	-0.942115643693883	0.10287540642327	-0.877672521479846	0.180784788575223	-1.03835090957552	0.0642228964514931
**Sulf2**	-1.1581518808232	0.000271679292297116	-1.09961554679219	0.00737256328895345	-1.15984835473541	0.00562626415397147	-0.364417221167044	0.46715569173677	-0.327094097275948	0.523717927747315	-0.476672570153365	0.285929767223559	-0.734570042077894	0.0608141653841906	-0.67191189970722	0.131165656317074	-0.709554311692312	0.0817924028462642

**Palm**	-1.09932189739869	0.000200837229414744	-1.04463220900781	0.00583382407449106	-1.02645791692046	0.00965408441077984	-0.248299573084996	0.614576811776348	-0.209532631467053	0.668273345283901	-0.19194889732152	0.684114482727949	-0.961936993502358	0.00472126324709153	-0.903588867407321	0.0331444976037381	-0.882719682569893	0.036217965712668
**Gpr65**	-1.07144273636291	0.000345660262193752	-1.00929250514636	0.00763254673281152	-1.07474345720467	0.00451163278639712	-1.33038175280802	0.000170231810065701	-1.28175647177576	0.0100530659025368	-1.35464213281438	0.01095490859706	-0.899053130461833	0.00996393430164051	-0.827969386969908	0.0487461012672929	-0.912418994940529	0.0239694124850124
**Ngp**	-3.04792116842629	0.000129385259456121	-3.01265066220347	0.00811660933259695	-2.28530925189522	0.0121689366004148	-3.84162795991837	4.51665136787884e-05	-3.81411441185389	0.0185827775753221	-3.15597960022915	0.0230085342577865	-4.28275195403292	6.83549331055715e-07	-4.2327244070149	0.00815777762838474	-3.42566684976091	0.00905106620742008
**Igkv8-30**	-1.23205455148443	0.000239234298622465	-1.1753784002616	0.0135082612030099	-1.27513754456037	0.00402437985678596	-0.188023086009369	0.755956725444839	-0.139747750131315	0.83410263800364	-0.192037369763505	0.714736578352379	-0.719074548826485	0.0903299817738636	-0.651642670604741	0.209129361996107	-0.828652250331902	0.0582044941116967
**Klra7**	-2.7766763908853	0.000179296816459652	-2.68471675673236	0.00673473647145142	-2.89150917107837	0.0114274609321933	NA	NA	NA	NA	NA	NA	NA	NA	NA	NA	NA	NA
**Dnajb4**	-1.09371347987809	0.000210357760810709	-1.03876747710207	0.00632071768223431	-1.03144473946937	0.0107630653199345	-0.571938961812843	0.164653312877736	-0.529081428844631	0.228940782606587	-0.583286489011622	0.199940017428141	-0.0911408202913813	0.863984978989586	-0.029464021988818	0.958925809744713	-0.0220322004648455	0.962388691491902
**Hs3st1**	-1.7527877313857	0.00158087878672978	-1.69123196330987	0.00869834327874961	-1.97008936359489	0.00111468110568619	-2.52241968604952	8.53854365410017e-05	-2.46891859860905	0.00717976638997489	-2.45193556843219	0.00571403299705752	-2.19575375090261	0.000303180333046978	-2.12379493209245	0.00896077094455241	-2.16816443777786	0.00365839096819856
**Il1r2**	-3.36123319352434	5.87966460901183e-05	-3.29838282494514	0.0081667470481865	-3.2127117749002	0.0320627535312718	-2.5500661736684	0.0170083517489357	-2.46505473727307	0.086072001886295	-3.60136103506605	0.0489471366914699	NA	NA	NA	NA	NA	NA
**D11Wsu47e**	1.02222762917678	0.00049073828463728	1.08177722821151	0.0043773881673312	1.04761129536926	0.00862851835263256	0.78672346949986	0.0380670028255372	0.832798367063004	0.0511817418372117	0.846595107517114	0.0702699130307406	0.967369802937679	0.00334760751430712	1.03226738392685	0.0142224639383495	1.02106892789234	0.0174903288475954
**Hlx**	-1.29103081101005	0.000244685921451531	-1.23227785615594	0.00665430685354208	-1.275782441154	0.0114766078461825	-0.0733922374760957	0.917605473349281	-0.034678150449345	0.962533927574941	-0.102678976212363	0.868279029056309	-0.0556875576934516	0.93302306437913 1	0.0068730774659 0032	0.991709916053324	-0.171212205867773	0.7226593705163
**Il12rb2**	-1.60584813482509	0.000342229296427887	-1.54506017717142	0.00751224960804149	-1.59507960000145	0.00754688846737107	-0.470789589369033	0.504903683916716	-0.431411526096327	0.547696036713125	-0.66589627247658	0.259226330507705	-0.687863812373696	0.247815294243034	-0.620478670003274	0.320187555459664	-0.854848349201681	0.109511550476197
**Fabp5**	-1.06728586561598	0.00029945538725386	-1.0135274157345	0.00714913554651359	-1.03810445530867	0.00951290275386434	0.855260601457626	0.0217547562759803	0.893448050219154	0.041576030809157	0.840218789266398	0.0277351424659676	-0.346205884903798	0.413321024813042	-0.283428600857337	0.517425082322992	-0.406365966925871	0.287322330507626
**Epsti1**	-1.20483114111525	0.000797416177213634	-1.15156995778003	0.00665430685354208	-1.1703561630129	0.0038756120838036	0.0723751435745265	0.920581824070295	0.106945888776981	0.854725291964856	-0.0105172526123837	0.98620937014665	-0.186032502499569	0.75787277030023	-0.123368723112745	0.824143846688636	-0.224893087392084	0.61857435204252
**Tspan3**	-1.53483426784458	0.00081564227690017	-1.4779664911047	0.0056698847999255	-1.48349430938981	0.00471865112760224	-0.0771693249807923	0.933870963813679	-0.0479527016515824	0.955591358972648	-0.228426043203343	0.709227894514271	-0.28299914696036	0.706741999142883	-0.222664675017637	0.734872285707172	-0.353666166678748	0.483767978289106
**Fosb**	-2.12911716453508	0.000856628642410652	-2.06214767856199	0.0109208771617732	-2.45498203471137	0.00235735940471891	0.351283841902845	0.757474678327364	0.383885757743914	0.700372467284978	0.24953512676007	0.735766552754279	1.68413646460435	0.0260403675247897	1.74549478087412	0.0506839557627486	1.64842936737862	0.00726089061074744
**Igha**	-1.86310387910589	0.000275555764650425	-1.80148330095511	0.00811660933259695	-1.59596956061195	0.0100519910942612	-0.202604750649119	0.834213798553321	-0.157436831271657	0.86316978464481	0.0493376377793323	0.957264870282747	-1.64279620453188	0.00601667032547097	-1.57337186688094	0.0392549702139373	-1.52738081435602	0.0262447659328138
**Rasip1**	-1.6102400196681	0.000237869102141912	-1.54381818921889	0.00565961832299351	-1.51957709119693	0.0169291169886089	NA	NA	NA	NA	NA	NA	NA	NA	NA	NA	NA	NA
**1700025G04Rik**	-1.46165166676055	0.000288350180724417	-1.39794971557957	0.00668763962045129	-1.43868057010949	0.011998855904816	-0.0796777109106244	0.92179992774469	-0.0304185641465018	0.97071028334936	-0.0966056691598413	0.88819192331466	-0.44401997389689	0.44445987990151	-0.37265730142333	0.530408868528338	-0.593084481271279	0.245008685260958
**Eno3**	-1.06504449206717	0.000210357760810709	-1.0069639992391	0.00764920770459561	-1.04669036211204	0.01551997661344	0.595417971789457	0.128288408900275	0.631660215704955	0.137837850850225	0.388776186639557	0.380629806920722	0.0561635325296 46	0.915152381548181	0.120369236802979	0.806845330355522	-0.00416654667041861	0.992974183379758
**Ighj1**	-1.47850303742253	0.00029945538725386	-1.41893672355297	0.00728166074737805	-1.37852018991075	0.0115804430130947	-0.30150349229742	0.658111482317494	-0.258652363484991	0.684074308443701	-0.273216019533553	0.636413184497177	NA	NA	NA	NA	NA	NA
**Scin**	-1.6560281813386	0.000240764788968668	-1.60259913441645	0.00719888555784092	-1.59207569578747	0.0170253342700428	0.0289730479399247	0.974526020103657	0.0628776422002426	0.948262778867156	-0.0420069407573114	0.957489634272801	-0.193090440530722	0.80461320307650 5	-0.133910934913467	0.870711406270818	-0.437853328408847	0.446439102772867
**Tmem150c**	-1.52728057487853	0.000259884789845115	-1.47187147386596	0.00708501366422966	-1.47017183486915	0.0161039558215779	-0.265694188367101	0.715995303017258	-0.224328180697385	0.755301172970668	-0.285217551166197	0.670936048458067	-0.166346138628542	0.817262116731704	-0.10545275201349	0.891669690437658	-0.309829233880338	0.575363246405132
**Ltf**	-2.80772646754777	0.000228160654528222	-2.76988943669052	0.00930856106206629	-2.08601198657193	0.0143441094120014	-3.5629147972535	7.79392815752509e-05	-3.52675768011935	0.0194273858772079	-2.80018735084057	0.0293359867470452	-3.92708002372655	1.70302373826708e-06	-3.87819021524574	0.00827183148337801	-3.19993950203202	0.00932106195007179
**Klrb1f**	-1.3852111633482	0.000200407882065939	-1.32282423127212	0.00673473647145142	-1.27844922660777	0.023018047175563	-0.307639347263596	0.621476521139679	-0.264117406599528	0.672998739770068	-0.287940214696803	0.657244438226 44	-0.15792013468914	0.807784883749887	-0.0896988666984012	0.896033254857874	-0.141704016679189	0.807204670204084
**Palld**	-1.35725995949512	0.00029945538725386	-1.29933811191109	0.00751224960804149	-1.30266813284049	0.0143441094120014	0.425579558993573	0.458022703932433	0.465960004514216	0.396808309143573	0.444975260341684	0.340329678686309	NA	NA	NA	NA	NA	NA
**ENSMUSG00000107872**	-1.68236151095369	0.000144484872131532	-1.62131462285275	0.0078687528760644	-1.53384564341682	0.0298612606818476	-0.0569665488933233	0.948636487236207	-0.0165133348858027	0.987161557603721	-0.0885302163798602	0.920078659852302	-0.45162209620278	0.494271634345311	-0.385183166064623	0.592866687557955	-0.445885472497312	0.495617318188896
**Heatr9**	-1.28877726418281	0.000298377734430276	-1.23569277823115	0.00673473647145142	-1.26450962020272	0.016925176285641	-0.0194731839623907	0.977962871897515	0.018898703743618	0.979798138303763	-0.087861599783352	0.892752059811434	0.324923822247833	0.536215997438035	0.386959401169726	0.427851521664269	0.294381603082925	0.508534795864125
**Cables1**	-2.41875072448336	0.000156417214883639	-2.34084922606443	0.00545239481192287	-2.11640397641542	0.0443150932687591	-1.49087447546435	0.0794316388390542	-1.44941954022079	0.119547270371625	-1.28511100283504	0.235853854574022	NA	NA	NA	NA	NA	NA
**Pxdc1**	1.00090390803086	0.000906977117187713	1.0608844129728	0.00714913554651359	1.06229494570209	0.00610470646602299	0.438686724278016	0.320205771462733	0.47837561505703	0.285337660051532	0.399686787426412	0.415434964148782	0.484853827036805	0.22212217804829 6	0.550533154942432	0.187956981547219	0.588685876641405	0.151888732625644
**Lrp8**	-1.34079469922368	0.000347976194639316	-1.28460285199323	0.00761229406835758	-1.29079209113814	0.015239890834081	-0.24052743241514	0.713234840449916	-0.202066646073466	0.753169420622554	-0.245676505496609	0.685963462114779	-0.231853174104711	0.704777248801226	-0.169311849416933	0.784205592861075	-0.337508621342353	0.501529936571088

**Sfi1**	1.35989369793882	0.000389491314094724	1.4124537633575	0.00664842113040577	1.37286465130514	0.0162387905801429	1.1824386972968	0.0142189765341452	1.21502022835775	0.0399781043679533	1.12943544976268	0.0913417391492545	1.28341883445917	0.00317661455427264	1.34111094974266	0.0197108872211615	1.22351586688371	0.0447788061930473
**Tmppe**	1.30261397731075	0.00052753254799087	1.35823671963721	0.00723181306300844	1.30881238431677	0.0126449460745021	1.40322657428738	0.00188978315885347	1.4407763238052	0.0153486220924168	1.47167139996567	0.0157856621201605	1.36732496057739	0.00101686872915241	1.426116004056874	0.0110458299950798	1.41128133078195	0.0117465005322007
**Klrb1a**	-1.89479360765186	0.000196703743905487	-1.83273397909484	0.00683150779396539	-1.70328174992453	0.0365209617534089	-0.723567030435736	0.335937274851017	-0.675701988717362	0.396855883377487	-0.674896114404288	0.42635189257796	-0.185583469301902	0.835490782640452	-0.117929744632814	0.899343291473177	-0.152281369732724	0.84102016299471
**Pls3**	-1.20933733126551	0.000790937525921508	-1.14697488497402	0.0109213044115088	-1.22100148065462	0.00653096047053452	-0.389293178488994	0.488523616685384	-0.344189773595374	0.546699294570273	-0.422251619704104	0.375385183855166	-0.535291651612592	0.262877300096581	-0.467433210433267	0.354770482284872	-0.726430239981724	0.0867964777491087
**Pdxk**	-1.21754108594123	0.000370010315806847	-1.15500987462845	0.0103094015345444	-1.12527805912563	0.017012583536674	-1.21143106072778	0.00416861048837472	-1.15812729053479	0.0384410040806681	-1.14901904687471	0.0606821962835477	-1.07636620237755	0.00650284284733504	-1.00816257260788	0.0460638339567121	-1.01710796120292	0.0469462095710456
**Laptm4b**	-1.4549349280619	0.000289484462926413	-1.40236840708986	0.00826198698005733	-1.35112324578537	0.0283528502949412	0.445227054200502	0.479286881369574	0.472872349898816	0.454329545251682	0.290084634492987	0.641560404899948	-0.263705917489165	0.683981838095664	-0.206261409160831	0.758756501719156	-0.264566377175169	0.65789909822558
**Arhgef10l**	-1.37510344262182	0.000523143694308202	-1.31204234927966	0.00906010814390769	-1.38083969489899	0.0145830705070256	-0.000405491961662945	0.999786915057498	0.0420007440164431	0.959342456847713	-0.173196742542047	0.796920471767276	-0.530761392933467	0.329466070162372	-0.465514425747532	0.41012451663925	-0.562232711919049	0.272662299976662
**Cxcr4**	-1.28669483801374	0.00168789558440245	-1.22723360747617	0.00839845727698544	-1.2448631110007	0.0049847273819326	-1.67066630321859	0.000509166017650362	-1.62418551195146	0.0095390040022465	-1.55465493504846	0.00851576833975158	-0.641279319644892	0.233862412779495	-0.57805591190718	0.253077509043353	-0.57409053917492	0.196520820501286
**Dmrta1**	-2.75168664501208	0.000523143694308202	-2.68586461805723	0.00998395805253311	-2.8226968161233	0.0138448372228722	-2.2137901547686	0.0280945190636139	-2.10664735248737	0.0744994053522871	-3.44377785980058	0.0211384659135404	NA	NA	NA	NA	NA	NA
**Xkrx**	1.10609993532596	0.00111168876688231	1.16167031542896	0.00863264395873153	1.13945410925077	0.00758313363390801	NA	NA	NA	NA	NA	NA	0.664176378308035	0.119276672399093	0.724719177231645	0.119600697645457	0.728703635847716	0.103368624141227
**Il15ra**	-1.09481366435475	0.000615072535435887	-1.04131823103081	0.00854975169815223	-1.06073592536187	0.0148454581199427	-0.0171711414064277	0.978261384235702	0.0196425366485869	0.977151407746554	-0.121646033646263	0.827822318513158	0.0337573136971505	0.957204462200966	0.0961595489857979	0.863090861742602	-0.00346229855715487	0.993311711733261
**Stap2**	-2.0402823973775	0.000693589142547658	-1.97709890500649	0.0160369887382978	-2.23823259688268	0.00708912940620372	-0.687503520995785	0.457821634142461	-0.637504157426079	0.532456952870625	-1.09874501791301	0.170111752706325	-0.30425563697457	0.758810085670877	-0.242227264838718	0.8210141612373	-0.546440455649657	0.381312512844868
**Tnfrsf9**	-1.15900985463772	0.000665655516629512	-1.09953538685562	0.0109592889304637	-1.14526238684835	0.0111711134628934	0.0240263029817201	0.970894615695564	0.0630801996135091	0.924799373788486	-0.0609825196180749	0.91907342846232	-0.325191764775676	0.521742559391639	-0.260181391590559	0.617287254280538	-0.464399512592449	0.277767544764108
**Pmepa1**	-1.28144727299848	0.000722243848154508	-1.21937980505649	0.010888514622585	-1.249575551783	0.0107050820854816	-0.754146956178458	0.140207476440998	-0.704833462981234	0.208046332335359	-0.728804333866065	0.182085025784465	-0.87507742424893	0.0567003153145149	-0.8039444864748	0.123523764163384	-1.00379232808078	0.0484405370063484
**Ighg2b**	-2.08499864873884	0.000371273182651918	-2.0417145950581	0.00862294794005553	-1.72042494736161	0.0280504630361148	-0.476431198695772	0.630210818342729	-0.445475622237585	0.641046968130767	-0.0730188395699263	0.944281158870328	-1.24638054173003	0.0938197314261342	-1.19328972863011	0.145025998868171	-0.961577028487547	0.207830952702895
**Coro2a**	-1.64664234964166	0.000697191681949997	-1.58143073211527	0.0137904691876379	-1.83509439496716	0.0097701241722403	0.322399758031476	0.69669576270978	0.355381277274391	0.662430321641101	0.208303603175985	0.760013461457831	0.0516334148678984	0.956981403120788	0.112211643147494	0.900946933868135	-0.00337800479742522	0.995531421228426
**Cdkn1a**	-1.61011129676689	0.000410051264370362	-1.55031421988657	0.00842006605790433	-1.58890419271243	0.0295776060870866	0.579770857783002	0.391504433656864	0.610953077306759	0.362952037183822	0.319881508280707	0.637494755322156	0.260006293506361	0.722183743138787	0.319325866961141	0.640936633670818	0.313208617480315	0.5994626235545
**B3galnt1**	-2.0279936903377	0.000868185818477836	-1.96311793839842	0.0112065697128045	-1.96854026688745	0.0121780256900324	-1.43411988298793	0.0696331057547758	-1.3579522326048	0.109152629077672	-2.08983190473666	0.0409974110929067	NA	NA	NA	NA	NA	NA
**Eno2**	-1.61373397882918	0.00054099564206331	-1.55770745071576	0.00999856822727384	-1.5915019477719	0.0219268059214746	0.031481580262725	0.972220942311153	0.0671551086241102	0.945636025563417	0.0180319509915881	0.982483394905436	-0.0952886998391728	0.908354481187724	-0.0355920878062079	0.967321178036833	-0.110706594588606	0.867906212974531
**Zfp882**	1.21861910758948	0.0012022841288552	1.28075418829154	0.00991845708580238	1.23615706911773	0.0100519910942612	NA	NA	NA	NA	NA	NA	NA	NA	NA	NA	NA	NA
**Maged1**	-1.13475345785714	0.00088749774616901	-1.07948108150216	0.0121419902523307	-1.11912074390253	0.0118527579936529	0.000959933897360996	0.9991049015002	0.0410242418112929	0.954373046741328	0.00369746151460095	0.993870056837843	-0.647461456381943	0.132932221693873	-0.584246928455449	0.215302523821866	-0.743697200134368	0.0890441401031178
**Tnfsf12**	-1.83257004914823	0.000760977295530518	-1.76376260654238	0.0103419272987679	-1.81869106323575	0.0173523953736081	-0.119661030219426	0.911207207526181	-0.0719841990608385	0.947052602246831	-0.0652581636647454	0.941728047849832	NA	NA	NA	NA	NA	NA
**Upb1**	-1.77391594058502	0.00081564227690017	-1.7192933815103	0.0139442948380235	-1.72600033975277	0.0139721170471235	-0.769437869683325	0.315286466830105	-0.711543578638203	0.396804957225619	-0.997884426227254	0.221555261968455	-1.03201006633669	0.119335386501816	-0.967579127682697	0.199696655444222	-0.965533346216908	0.138215289549622
**Sema4c**	-1.62911330112122	0.000906977117187713	-1.56175820243961	0.0128303787046969	-1.60316012688854	0.0154487055392317	-0.412294443868835	0.604537713874041	-0.363124971967996	0.646473717955188	-0.495224760260252	0.467460819828553	-0.544314377125506	0.430321907597362	-0.477228654820335	0.495057166146015	-0.540363076183194	0.339666474568715
**Dapk2**	-1.7421452569267	0.000504151127731648	-1.68362969729422	0.00929318568686523	-1.63801698343837	0.0387405383398693	0.117028024497576	0.904351305006813	0.150044932740461	0.865634855837405	0.00128301445965108	0.998663776757896	NA	NA	NA	NA	NA	NA
**Strip2**	-1.69497793670586	0.000450613186676095	-1.63851517507593	0.0158089761442511	-1.64446351503322	0.0264082201120163	-0.0739315956587788	0.939518429686817	-0.049803470940303	0.964112856329413	-0.225070831612401	0.787678871564803	-0.157037715066478	0.853351519954868	-0.101230944842167	0.916007156332835	-0.136900229010129	0.848116878415031
**Parp11**	1.01336929687012	0.00137214221564945	1.07353884767563	0.00756396243603009	1.01490002242911	0.0182304758347688	1.37540364646524	0.000153425707710491	1.41753031982786	0.00595254201893344	1.30626014455796	0.0117433883103347	1.14010202136861	0.00102105648293747	1.20443217403738	0.0097975948913897	1.14060569372283	0.015615863626166
**Cd300lf**	-1.0566879784904	0.00107749173739208	-1.00008817554282	0.0161661669749625	-1.04557477834765	0.0113590255862099	-0.782240739365391	0.0636830154395587	-0.737093073607002	0.13580965761257	-0.787953498518547	0.109403623649178	0.0291976507311793	0.964531128797426	0.0918010348341322	0.877676543617382	-0.00569666800430877	0.989744440986673
**Itgax**	-1.4965480539983	0.00284365577445068	-1.43610954856477	0.017796186038713	-1.62303755369537	0.00417191728187469	-0.587316046712369	0.442826860850898	-0.544756252809704	0.464941687425684	-0.631467433926131	0.288080099484374	-0.378195406248052	0.623712461906136	-0.315907048084467	0.671886717570836	-0.416046715845417	0.429206458952655
**Lima1**	-1.31074178162137	0.000517470187956626	-1.25733954231345	0.0144313573333112	-1.1878603501462	0.0326639015608921	0.0921942300329588	0.901373229134279	0.126918251712629	0.861340668280757	0.230640046997846	0.716195475193773	-0.226001539602753	0.712988112861361	-0.169159398934952	0.803165567413656	-0.138286726178788	0.816464373145828
**Mfge8**	-1.28070399990437	0.000817689020356757	-1.21988128659927	0.0144313573333112	-1.26381713861982	0.020834928693795	0.588091596185319	0.280650990328808	0.620979813000974	0.28369974508069	0.155098520481641	0.813514289121518	-0.949942513060077	0.0374772117796575	-0.884533928425484	0.108449597248141	-1.0474739253092	0.066575546495337

**Ugt1a7c**	-1.77803466797297	0.00140084487266778	-1.71026049951051	0.0160640307032691	-1.78241316922572	0.0114766078461825	-1.40236553659487	0.0492843505599057	-1.33117013553828	0.103363492134094	-1.68918547754468	0.06399354181518	-0.566924757271881	0.473481974003481	-0.496360209990133	0.525457455482362	-0.593822893493217	0.332724062567811
**Lyz1**	-1.53775195749997	0.000741571942088551	-1.48036685032755	0.0103094015345444	-1.40328858547137	0.0342791293534177	0.334346967108906	0.657621971024979	0.370381621940218	0.603532060840417	0.336960498938362	0.598526442394484	-1.93608550118642	0.000111340446842104	-1.87346742901805	0.00827183148337801	-1.81372701677558	0.0210976311282157
**Crtam**	-1.37924545570256	0.000792142051636077	-1.32651748376075	0.0100456532772056	-1.23104966514877	0.0335999705356039	0.232706278875525	0.744888674194147	0.275166255416679	0.676310729698147	0.412363411516175	0.458959246284623	-0.415957460102223	0.483929878038049	-0.352577664550037	0.560127230185316	-0.35885243974642	0.515930261444621
**Rhoc**	-1.7782952919471	0.000885672186398468	-1.71668463012439	0.010888514622585	-1.7414381120883	0.0309404097777033	0.90628674718394	0.212203134717568	0.933904638494765	0.200320090843929	0.269952219286283	0.722068355939411	NA	NA	NA	NA	NA	NA
**Arhgap28**	-1.47178161057585	0.000960646590434084	-1.41533463738647	0.0132401475917592	-1.47639207837578	0.0242882591500052	0.220456116568824	0.780646238299242	0.251611688910995	0.738509331442512	-0.183191580389755	0.81229570770037	-0.307011251301765	0.659016415878194	-0.245135111663369	0.733747654083562	-0.466993076755215	0.428699483282572
**Ifitm6**	-2.28710232542846	0.000763385820812633	-2.24302398540536	0.0141343164627232	-1.94636344045227	0.0288101956120599	-3.12337391872867	9.16999678147932e-05	-3.05678312464859	0.0154463378739369	-3.64419312382996	0.0174379361874671	-2.74357256122042	0.000231703602917219	-2.68602988264339	0.0141018284008989	-2.35165553759525	0.0309302760825675
**ENSMUSG00000095200**	-1.06550766547168	0.00174436752224415	-1.01045841481031	0.0185083538764463	-1.03183243564595	0.00963504534934042	-0.750694573523307	0.092144585466518	-0.711724947220146	0.162426699707764	-0.778821766132213	0.0945234661699804	-1.30766473412049	0.000421977473338921	-1.24367308243741	0.0148692264093131	-1.38581017664197	0.00624828236291838
**Rtl8c**	-1.27248080173839	0.00178743131053341	-1.20741502315383	0.0180317356063225	-1.31566639083573	0.00978452699190465	-0.0544613452567471	0.94475207321815	-0.00437675583911618	0.995616322185165	-0.0249642144543022	0.968895346048455	-0.682302867768397	0.182960528920823	-0.612462278514422	0.2529401128444	-0.741124958568247	0.109678286537317
**Pfkfb3**	-1.19048433238288	0.00379202672975857	-1.13625333793237	0.0116853087915599	-1.12676915572965	0.00713960111357622	0.00169138125549746	0.998638246692943	0.0346638741966944	0.962300777052772	0.0101282172301076	0.986965715977297	-0.181953979037318	0.793437968724694	-0.122093899122713	0.841044431685756	-0.195836387442637	0.681426848878359
**Adamts14**	-1.45692869659494	0.000578094205030769	-1.39613699575333	0.0204228915735425	-1.45598310552414	0.02683029421574	-0.0484002040870019	0.962799767969992	-0.00621592236773607	0.994586820526896	-0.146654364782067	0.860186386092133	0.257074506579149	0.769173006214342	0.321926982011335	0.663875915523219	0.153216538941949	0.81352135407154
**AI467606**	1.06450581717434	0.00289311010688256	1.11670486588603	0.0138869199449606	1.23379114451732	0.00793305938065881	0.965150189256619	0.0308787146080657	1.00748089663895	0.0533075532551058	1.17905180165602	0.0365605512048669	0.622611935101553	0.166563840478013	0.683557781659779	0.162906720657545	0.809953099591887	0.104256917445024
**Obscn**	-1.49265682067235	0.0012133395336947	-1.43455574038156	0.013252655958697	-1.46896505074679	0.0203390844062468	0.298537793988035	0.701024459713662	0.345974152156176	0.627518554261658	0.226519942403987	0.735610447080078	NA	NA	NA	NA	NA	NA
**Slc9a3r2**	-1.23244686975427	0.00108806235015916	-1.17684485140069	0.0120414451078053	-1.2295652284226	0.0254979927034338	0.656868378856477	0.203826946537776	0.694477295043886	0.190370558563505	0.525062537218294	0.290832551737145	-0.379245308732032	0.48750341642461	-0.316031852976562	0.571696269877621	-0.40719869112045	0.430971328308308
**Samd3**	-1.39297245996221	0.00100743887551081	-1.32866512936234	0.015818144930323	-1.33727344310954	0.0222557832672052	-0.234007850516911	0.753470794288044	-0.184488372409003	0.809883493157942	-0.178295019296438	0.809938538360219	-0.196974008031918	0.783891107345054	-0.131394604847815	0.865421758895182	-0.176176225993675	0.765389976516891
**Gng12**	-1.23816714804689	0.00155598532744131	-1.17747052891444	0.0150032488315969	-1.23698993786591	0.0158470070587525	-0.546113785354747	0.334096626692246	-0.505783858014539	0.391914187653577	-0.594171875800697	0.280086209881903	-0.366130452717731	0.521875568858741	-0.303991080105812	0.601808250212954	-0.345587835648828	0.472377850664852
**Slc2a6**	-1.1149337777331	0.0013776590176373	-1.05681097916766	0.0135452639480027	-1.07935089448096	0.0210982403818756	0.204597928521585	0.73300662022691	0.244130174782148	0.659116902258322	0.156963127745768	0.775591668348481	-0.0500252472135744	0.939240701502754	0.0142940943798034	0.982868479935305	-0.0579888716304457	0.911840233025313
**Cd177**	-2.53376861624381	0.00100540066047798	-2.49325443051943	0.00828867348889874	-1.95947862786249	0.0491631894006436	-3.35035930736709	0.000224771582316625	-3.29320418143382	0.0130323505756666	-2.84425379709036	0.0854580003638557	-3.45404144901268	3.93739079326087e-05	-3.40162953993406	0.00776135533548196	-2.78938977788362	0.0370455128750621
**Dyrk3**	-1.87675760717959	0.00149319872554307	-1.8184663132337	0.02485243146643	-1.89996572406153	0.0120753702907693	-2.4100437438796	0.000534685335719834	-2.33170920285564	0.0252434363233612	-3.85652880283048	0.00978763766536479	-1.0580337307528	0.151068101654837	-0.992610310294279	0.234827942677807	-1.00795495761157	0.199712666809513
**Zc3h12c**	-1.22575489558524	0.00153121287116697	-1.17033013611991	0.0175713061591177	-1.19707721998255	0.0166733474483664	-0.217839484893795	0.746706726648594	-0.187040434913486	0.782189227108815	-0.435868744271187	0.424431929135986	-0.468405588825468	0.380783914573815	-0.405501649415648	0.48080578231914	-0.505957818704047	0.287893942893931
**Myom1**	-1.98162001206334	0.00131805221609686	-1.92647662367436	0.0125507678718825	-1.93065284627839	0.0279921461548721	NA	NA	NA	NA	NA	NA	NA	NA	NA	NA	NA	NA
**Anxa1**	-1.87377966092282	0.0012741443088203	-1.83035030307246	0.013782747988037	-1.42611722119342	0.0267407982193012	-1.5372488268841	0.0403128405636257	-1.50634342809033	0.0902981034793485	-1.17489019464983	0.133481952147349	-1.94772133275231	0.00297568205642148	-1.89575166314615	0.0287968296382632	-1.47918068317391	0.0439170348658273
**Plxdc1**	-1.38387953386531	0.00201434638466887	-1.32055583555099	0.0177716536637906	-1.41785459427203	0.0134418193362435	0.0792524573714419	0.926859120729869	0.119204884866081	0.875090078278252	0.015739751397734	0.981839096081323	NA	NA	NA	NA	NA	NA
**B3gnt8**	1.19146366596456	0.00321558118928132	1.249349492097	0.0143762302578506	1.22756694241103	0.0104700980429014	0.200972168064496	0.780122480485448	0.250963371550262	0.706197122130576	0.27152846660974	0.710049399386129	0.494875875448214	0.371032291169614	0.556384959602954	0.319775117143343	0.461874100201616	0.407871588252496
**Srxn1**	-1.33479548822779	0.00210416394862667	-1.28172034154146	0.0168680843448356	-1.38527058579665	0.015639622948752	0.39035932824799	0.563461406658022	0.424004250754293	0.511771676902767	0.228588769449191	0.692755083483182	-0.13783379621478	0.851675977590641	-0.0772139559563914	0.917285583347869	-0.145412538099175	0.794589391486762
**Rab7b**	-1.4771393547767	0.00194456371087783	-1.4143064160086	0.0213939569655473	-1.60874344399086	0.013429611455716	0.160484259712542	0.856284630369655	0.203288441989889	0.811428393780193	0.135774209378294	0.851389107397631	-0.74016413237432	0.229461824012874	-0.672227036328409	0.330751748428653	-0.829542029378792	0.157092996769518
**Adam8**	-1.31533893011358	0.00333966800158869	-1.26384695607684	0.0122708314638107	-1.23768915039949	0.013691065277304	-0.105823116069717	0.903130029119121	-0.0740066638816636	0.922547267501945	-0.0631108181649807	0.928097516811666	-0.107309082743121	0.893382574350111	-0.0496404444306152	0.947966332242965	-0.0919031968153247	0.869125880345664
**Slc7a11**	-1.07839828988752	0.00201217047853174	-1.02164261804338	0.0177487649067322	-1.06899071502757	0.0172685959302165	-0.0849111431063148	0.900031219267568	-0.0430791095639592	0.951512532961315	-0.0609654446801867	0.91907342846232	-0.189913731656325	0.733508491127171	-0.123503057647068	0.830620643902788	-0.336246190310116	0.424781359080238
**Rab26**	-1.66808675259611	0.00113309735963615	-1.61076137368597	0.0117250207233363	-1.60305015832244	0.0469799665271426	NA	NA	NA	NA	NA	NA	NA	NA	NA	NA	NA	NA
**Id3**	1.09047710246994	0.00387147779293315	1.15094777336171	0.0122708314638107	1.12375924210903	0.0137884219689356	0.996822809712224	0.0339697342636836	1.03709547066383	0.0501335193998425	0.736427533382717	0.175038931641116	0.917343010971102	0.0362742880212545	0.986701486232129	0.0497646793273478	0.961993742184199	0.0509732196251139
**Trim13**	-2.23013559128054	0.00122497074442602	-2.16641592041685	0.0145573873657113	-2.08154110777548	0.0379828609870941	NA	NA	NA	NA	NA	NA	NA	NA	NA	NA	NA	NA
**H2bc8**	-	0.001516604	-	0.013306425	-	0.033731115	0.933643048593	0.100972753324	0.97202063193	0.11212305408	0.57924605185	0.29450068905	-	0.9176158693506	-	0.988605358757	-	0.874945787439

	1.40546209929819	0401597	1.34142130395939	621274	1.39878766460561	7655516	489	188	3971	8574	3309	8983	0.0806964733932836	65	0.0125080191165854	783	0.104711058094386	467
**Arhgef40**	-1.61239216933575	0.00277245643121779	-1.55711169999487	0.013306425621274	-1.5073511778242	0.0239361018268205	0.205757559498059	0.834213798553321	0.234597838551406	0.777170518135349	0.236150689181587	0.744116125141696	-0.288348243892862	0.742284703404561	-0.233128571115966	0.771061157689552	-0.311575484734435	0.625081813668477
**Adat1**	1.61616789333183	0.002546563449883	1.66486860013968	0.0157722773210707	1.57576509831853	0.0233991982838952	NA	NA	NA	NA	NA	NA	NA	NA	NA	NA	NA	NA
**Smim3**	-1.15867304997621	0.00286643826051202	-1.0987591640568	0.0208575314386643	-1.13461748194594	0.0160302029570764	-0.838141299934062	0.0970331199397327	-0.789163534658895	0.152124152164677	-0.782807451933772	0.148025314830987	-0.819426985725277	0.077632236748503	-0.751519169330345	0.135481932568879	-0.837116401966234	0.0756300035023848
**Ighj3**	-1.16732289125797	0.00244587696361803	-1.11183872410954	0.0203803799862369	-1.12655925855045	0.0192347888949642	-0.137375155457966	0.842978994057625	-0.0958204181650928	0.888594003909195	-0.0375352546690533	0.952626000718368	-1.24966977460301	0.00334005017672601	-1.18565361127775	0.026861834137238	-1.21266725435218	0.0229636292006764
**Stk32c**	-1.65368317153912	0.00225825323387681	-1.58954979293953	0.0177397028579971	-1.69946285131288	0.0247956034026489	-0.567916352469005	0.492620561508685	-0.528013943956174	0.523205048714656	-0.662354390605516	0.388387786837837	NA	NA	NA	NA	NA	NA
**Tmbim1**	-1.2510987231737	0.00313174838030689	-1.18888121758737	0.0219522970865145	-1.25102712263823	0.0153237393132371	-0.359898333371531	0.587679764071414	-0.316550614234566	0.621469332155159	-0.431253885671313	0.426505206015418	NA	NA	NA	NA	NA	NA
**Asph**	-1.08746202963048	0.00409957092644175	-1.03412135271339	0.021901140804179	-1.16438978063999	0.0123423052851024	-0.154072622882935	0.820985465470716	-0.108627153051667	0.868333123059486	-0.15642724095285	0.787890596663163	-0.619689328633447	0.19237169195156	-0.553975068393345	0.270864641347474	-0.618273322578585	0.161069700571351
**Ighe**	-3.84189727930805	0.00982604816238861	-3.79110687996035	0.013252655958697	-4.26673696987306	0.00954597616458863	NA	NA	NA	NA	NA	NA	NA	NA	NA	NA	NA	NA
**Wdcp**	1.60827636733243	0.00385746377492893	1.6561099462017	0.0192102437182932	1.83553106465667	0.0177481527692009	NA	NA	NA	NA	NA	NA	NA	NA	NA	NA	NA	NA
**Insm1**	-1.23888294187827	0.00270183583751278	-1.17629178105338	0.0187960353437691	-1.25814153570699	0.0290437833009546	0.611209416668204	0.280317785092256	0.656248009116741	0.246376240748803	0.528651956739429	0.304825899957182	0.365522334225323	0.536215997438035	0.43302068969699	0.432093566200601	0.241469076070326	0.635077334992522
**Coq10b**	-2.24272398127306	0.0194845816531494	-2.16825375042187	0.0265801259052917	-2.39751733406251	0.00297042564487324	-2.50698870531538	0.0320701192255931	-2.40771328206607	0.0525379510219477	-4.36715792762344	0.00830379343178575	-1.35400797237544	0.266775012610049	-1.28364724873555	0.222290755975899	-1.33699442923101	0.0829802438654505
**Mycl**	-1.21912356978975	0.00566165446113693	-1.16156022937493	0.0296909868586502	-1.36728885110827	0.00926490096969248	0.142137424759352	0.859582659673207	0.184580679456854	0.804893627217937	0.107251977117242	0.862518893181915	-0.574066624323972	0.326909522106061	-0.510222519351503	0.408747289405381	-0.665054861212045	0.16092415169802
**Avpi1**	-1.35030049482471	0.00281350639366088	-1.29245786158761	0.0197642717044128	-1.28426516002169	0.0296842321304192	-0.496919351315263	0.467722760194934	-0.449140482949172	0.516835297620421	-0.364494512575776	0.590313548948248	-0.241856778674321	0.73681856207095	-0.176602597635277	0.805741307888763	-0.278961975145654	0.624669717797973
**Ptprf**	-1.11409431716348	0.00406886499541319	-1.05749729246092	0.0242546626475979	-1.11679407116373	0.0182304758347688	0.168621973795933	0.802911790355791	0.209617451286695	0.728596478721705	0.0661997633694834	0.911598491623973	NA	NA	NA	NA	NA	NA
**5730409E04Rik**	-1.13693629172831	0.00356494292009874	-1.07012351789249	0.0235036724959241	-1.11604796185609	0.0220043835326129	NA	NA	NA	NA	NA	NA	NA	NA	NA	NA	NA	NA
**Emilin2**	-1.09837379506998	0.00401316856621514	-1.04012474247704	0.0336696654243538	-1.07351586575875	0.0137157957711063	-1.00530186878201	0.0358115937398229	-0.954665875687192	0.110989310406349	-1.0775279230648	0.0605490848775693	-0.741726608608814	0.112836897635484	-0.679031241958505	0.21859266147113	-0.706946214327635	0.108234930178463
**Zfhx3**	-1.54092090460681	0.00456842180409921	-1.47893339297489	0.0244631450222759	-1.67922473832859	0.0174552217221314	-0.149803017270391	0.882718796711588	-0.116257233974152	0.900572140439754	-0.391618479904104	0.586915089598797	NA	NA	NA	NA	NA	NA
**H2-M2**	-1.19368523841696	0.00604566123960253	-1.13528774170786	0.0180095321153682	-1.18197534870621	0.0181914555861832	0.917425739722996	0.101434786977913	0.952547188096456	0.0859331385188507	0.725078897927545	0.159945245238893	0.135458014587559	0.856159768283142	0.199145281271679	0.74598483687547	-0.00600243090967837	0.992361346089471
**Klk4**	-2.71900263166835	0.00349502466973928	-2.64468573538887	0.024880835251284	-2.99026067415056	0.0229720312821807	NA	NA	NA	NA	NA	NA	NA	NA	NA	NA	NA	NA
**Bend4**	-1.35910651839278	0.00325907843727569	-1.29961817567506	0.0212172859288308	-1.30240541934385	0.029004553504824	-0.0984174788707228	0.912636530765282	-0.0570468991179883	0.949142593984235	-0.108265465256343	0.88466526210672	NA	NA	NA	NA	NA	NA
**Ildr1**	-1.75275149696596	0.00630953057339154	-1.68390373347827	0.0306108942523451	-1.81503564256615	0.0114766078461825	-0.846280771397171	0.353375232586572	-0.827771998920248	0.382698218755274	-1.63188508243322	0.0535478473668574	-1.6152076140658	0.0261942165281169	-1.54216550013879	0.0742358818866263	-1.57906129538433	0.036471668490158
**Dnah2**	-1.62150980262879	0.00552651111735183	-1.56742217699189	0.0263150831716867	-1.60011319040355	0.0160972719398619	-0.731844889017573	0.388163471166588	-0.698935116560363	0.408857899091202	-0.84620072955726	0.227448089566984	NA	NA	NA	NA	NA	NA
**Ifitm2**	-1.50202676576121	0.00312222394308225	-1.44649773894625	0.0265671617079814	-1.44875044585038	0.0285042852295957	-1.00115503102388	0.135311481669491	-0.970868760590908	0.215612677962068	-1.58293020082918	0.0658740742705161	-0.705412178674532	0.29410563303566	-0.644973048288905	0.389054177959926	-0.620793134484933	0.324428196720702
**Cxcr6**	1.09407673808389	0.00657990649129542	1.15031414496378	0.0188532845956126	1.18498987377084	0.0203390844062468	1.48438742272316	0.00131049027411846	1.52444898119237	0.0121503263811975	1.53965929951449	0.0120807210102123	0.330021260195747	0.579289558186422	0.396128768136923	0.484981461405183	0.4658190955756	0.432246258005377
**Eepd1**	-2.28415473803584	0.00404897407187068	-2.22197548712097	0.0365304212475873	-2.35425893846993	0.0181914555861832	-1.37501822767771	0.335119449399405	-1.30782695331347	0.299717263745541	-3.38160613762802	0.0415393258077381	-1.16849466304768	0.391626000498771	-1.11274067406818	0.336626292255315	-1.22812692135264	0.194932979832227
**Ifit1bl1**	2.0774765604877	0.00480758205208196	2.12787489243108	0.031680967822264	2.45640014654816	0.0201581615508652	NA	NA	NA	NA	NA	NA	NA	NA	NA	NA	NA	NA
**Il3ra**	-1.27782591697327	0.00524361501081047	-1.21244992767361	0.0272639259676459	-1.3019335169804	0.023125745174876	0.181798545922996	0.821637221562661	0.230549741567319	0.744461873331411	0.201503176691537	0.745808141009239	NA	NA	NA	NA	NA	NA
**Loxl3**	-1.3986609657046	0.00429366089106216	-1.33920531237712	0.0252832015351199	-1.4164106392008	0.0320666163981647	-0.29445981778666	0.720032371536037	-0.257956384834046	0.749634930614835	-0.508421928088317	0.472345580523959	0.290900253225274	0.701546793433826	0.351698102737054	0.62107011912274	0.208975874601856	0.729001089392341
**Serpinb9b**	-1.65249119742247	0.00746436370094355	-1.58097947860438	0.0274439025708944	-1.61737161450185	0.0188802154209773	NA	NA	NA	NA	NA	NA	NA	NA	NA	NA	NA	NA
**Gpr157**	-1.26789138247832	0.00495344462419436	-1.20542078661534	0.0269601504045026	-1.25971354715092	0.0310294503862844	-0.242370037395107	0.752847091399722	-0.201740690722379	0.79071898806054	-0.42925780099606	0.506260128940416	0.0150539217360533	0.987872568344387	0.0807232878477627	0.91977732966792	-0.162492119662298	0.784093748745264

**Lgals1**	-1.358367764219	0.00706833982671505	-1.29852332115409	0.0212172859288308	-1.25636521264146	0.0285929165653269	0.166840868679945	0.857270550827729	0.212581245647417	0.779486944726586	0.231696733297515	0.737042966910361	-0.453115888271146	0.538695223035506	-0.38514610074707	0.569708241591579	-0.618327790128323	0.2811829309755
**P2ry14**	-2.0530774539942	0.00438441669426712	-2.00082697402196	0.0386719516330419	-2.04298696256716	0.0266129704797845	-1.75806955447006	0.149571809759629	-1.68466418907065	0.146373333737761	-3.4228633581317	0.0455459133197927	-1.06250822629738	0.39980843548798	-1.00615436830225	0.343636021131999	-1.01412696046581	0.264206022213242
**Pdcd1lg2**	-1.20883129429352	0.00629448087342782	-1.14125922969257	0.0321967233451127	-1.17984915343451	0.0223072231620282	-0.691857670716358	0.252911462243972	-0.636176634911896	0.307877752287073	-0.726904670361523	0.224742987450069	-0.793578384464309	0.138608113061469	-0.723203133680825	0.196645603797421	-0.752834450838608	0.132519643933559
**Zfp324**	1.45493712006122	0.0135271164811334	1.50546294912455	0.0382022889120511	1.95328843844878	0.00909796157168972	NA	NA	NA	NA	NA	NA	NA	NA	NA	NA	NA	NA
**Kifc3**	-1.0906531825489	0.00704051326579639	-1.033715262233	0.0321443780906595	-1.11846949378375	0.0221564795797524	0.00759731112192893	0.992434868470945	0.0506684977184475	0.948994471901732	0.0159912911503985	0.979696660653879	-0.415300087602073	0.453478861558905	-0.352146132763791	0.521690603748814	-0.390647243345764	0.402523888415536
**Cd14**	-2.05004615201975	0.0140568898930898	-1.99090048834609	0.0297249749888637	-2.39495641342365	0.0125790962578883	-3.00452217035307	0.00184307508812541	-2.93427675877553	0.0229693570050341	-2.83373324162183	0.04415393684749	-1.43917380505706	0.157481135734847	-1.37314192769556	0.17853753346192	-1.2449881905601	0.116464448332602
**Pla2g4f**	-1.58134067714063	0.00484576900653509	-1.51917897348553	0.0274307822956054	-1.60133514896129	0.0401747268781525	0.0303125699633836	0.977767823770427	0.0660628037078223	0.952282493825649	-0.044009553882287	0.963759935234916	NA	NA	NA	NA	NA	NA
**Cpq**	-1.16725544220692	0.00805262060730498	-1.10509388108446	0.0382022889120511	-1.26463203554202	0.0174484769952837	0.0825627794162094	0.921364745931358	0.124232949138114	0.868270856876433	0.0806398276778474	0.902361841743903	-0.443628355461384	0.462538646435263	-0.378554423440011	0.535742123427315	-0.50355438670214	0.299844707150844
**Pik3cb**	-1.07608307070908	0.00859816488638721	-1.01434098975405	0.0383335585446413	-1.16537833127877	0.0178838722551379	-0.222298614146299	0.748128917285134	-0.179582624975379	0.796318445169926	-0.217418426605804	0.71222026259624	-0.286145294834084	0.640371318521925	-0.222715744691337	0.726027886514748	-0.368741961888701	0.435483817767735
**2200002D01Rik**	-1.6245298612867	0.0053506298991126	-1.56495704959801	0.0285928213102164	-1.62384877585154	0.0406001782780473	-0.0263068684176132	0.981344430276997	0.0274760801582626	0.98137775774201	-0.0830869060769357	0.933919358788956	NA	NA	NA	NA	NA	NA
**Bbc3**	-1.1887702093805	0.00999252709076655	-1.12658788305799	0.0381181536670625	-1.31357801856626	0.0166904457857899	-0.690587460300329	0.272325736809167	-0.645940519472801	0.336611398910492	-0.757492720287001	0.211427500892047	-0.461882701355549	0.468311887593284	-0.393182271793365	0.549454040600751	-0.58783338096853	0.249021298258314
**Fndc5**	-1.28197573195345	0.0091197081537811	-1.22631230436793	0.0342487793042432	-1.34702141091176	0.0212321020728175	0.232782092020383	0.779459765263883	0.267708141089088	0.722395255100507	0.130413520566495	0.849456080888124	NA	NA	NA	NA	NA	NA
**Adam23**	-1.17129556180152	0.015835071289892	-1.11394421853924	0.0300830086353313	-1.20126435693264	0.0144788646977032	0.12913354588823	0.886346265624896	0.161022026336989	0.824776370727615	0.0542408011321023	0.935749834858659	-0.205594737319051	0.795687306424307	-0.144791195984118	0.838887170695593	-0.267080460257238	0.597392002033831
**Cfap126**	-1.33759736284591	0.00630953057339154	-1.26889111610365	0.0259776089370918	-1.22468847295776	0.0427153418239365	NA	NA	NA	NA	NA	NA	NA	NA	NA	NA	NA	NA
**Ccr2**	-1.16565533716043	0.00410333396394733	-1.11211402293802	0.0438521305881802	-1.09588875385254	0.043647748822754	-0.162944460671641	0.82553643444878	-0.117645484152379	0.88497241935072	-0.0825204777837	0.91617128633446	-0.596525064045755	0.260843942163518	-0.536569514205362	0.405598290264214	-0.691228432726973	0.217351801500141
**Cttnbp2nl**	-1.39950783873405	0.0049452610187276	-1.34288926105691	0.0366566833206568	-1.40885309679344	0.0442111386131611	0.265409636135612	0.754981161278191	0.295256054541144	0.731425651957846	0.107190166385681	0.898626551491742	0.107227152713724	0.901400202543181	0.165688274328736	0.854663800239109	-0.0822602829292229	0.913217953357598
**Rnd3**	-1.12745253998684	0.00866853819359588	-1.07304966990282	0.0417024675745202	-1.12552111166851	0.0224144514485798	-0.735448581575377	0.201997792314098	-0.687559718388301	0.286984301811403	-0.797365805657358	0.184637844996997	-0.497560284618339	0.387601045394307	-0.436679980740292	0.487552479505663	-0.540111360970531	0.264414062203894
**Zfp748**	1.05254921920617	0.0176518942603644	1.1087298247309	0.0400251110826795	1.08516011065448	0.0115247460257577	-0.612185960259173	0.326560315643374	-0.55390643242106	0.411333560815524	-0.742000641119215	0.301995235355384	0.337226028781936	0.600836802878412	0.402158347333468	0.530868298684158	0.385368117430173	0.463330704912201
**Tnfrsf4**	-1.10377535079817	0.00540945275697467	-1.04133337147626	0.0400148916828321	-1.04956640159554	0.0389146601861479	0.302352705873847	0.633960070267723	0.347952147329636	0.594794278363936	0.246090242628752	0.675775132439381	-0.148186393128624	0.823815564283619	-0.0781114921037464	0.916166889072253	-0.373337911328779	0.471706293457559
**Lyz2**	-1.27353496974795	0.00957649702524855	-1.22172337952366	0.0337593026591819	-1.05854998288727	0.0267225792893725	-0.842272546967932	0.20445847036234	-0.803035447648063	0.248496584959181	-0.655577053835755	0.252930250024567	-1.96897097208981	0.000145692918705203	-1.91059960901564	0.0101670721724138	-1.78176370094338	0.0051796811861878
**Camp**	-2.23020185292124	0.0129411354941832	-2.18802390567279	0.022288220327762	-1.94671632798272	0.0350083118721452	-3.40655040928325	0.000888585241082444	-3.32622107723324	0.0162654363471408	-3.49966378705965	0.0612212757042653	-3.67595316023568	9.44966076050935e-05	-3.61685851931274	0.00776135533548196	-3.27953087837184	0.023152815524319
**Rasal2**	-1.31768554337654	0.00842814891881342	-1.25346052116353	0.03563279860833	-1.34452587743876	0.0337311157655516	-0.0417744425345219	0.963762738302943	0.00278568348817414	0.997702840838366	0.0137422406134929	0.986514846800589	NA	NA	NA	NA	NA	NA
**Batf3**	-1.07586679626236	0.0112489272697328	-1.01459011783163	0.0403697888292702	-1.16980553083232	0.0223663906057767	0.204172728163941	0.776719315976751	0.245198208309484	0.714135313978795	0.106124643452643	0.867434240734449	-0.0140497850831896	0.987872568344387	0.0514088708151854	0.946672901113133	-0.187227145761696	0.717517615818998
**Gm12253**	-1.99800489554227	0.00754282891849581	-1.94110959133157	0.0300443735088275	-1.91482901335438	0.048291508365209	NA	NA	NA	NA	NA	NA	NA	NA	NA	NA	NA	NA
**Adnp**	1.30962312180736	0.0108762490086321	1.36422532204676	0.0295861638899296	1.32494809467781	0.0350638218328958	NA	NA	NA	NA	NA	NA	NA	NA	NA	NA	NA	NA
**Ccnj**	1.1475613074928	0.0136258992660562	1.20664697296879	0.031680967822264	1.18076839218248	0.0264344590373635	NA	NA	NA	NA	NA	NA	0.948801245706298	0.0804775370384958	1.0106755527233	0.10083798906465	0.992408817184177	0.0839073464925427
**Zbtb20**	-1.36136557982012	0.00883012061282925	-1.30838851428063	0.0332218370247458	-1.22640961655701	0.0443065466359265	NA	NA	NA	NA	NA	NA	NA	NA	NA	NA	NA	NA
**Shb**	-1.32290537964296	0.0108812915354986	-1.2715328091447	0.0338134406552121	-1.35636566949606	0.0360257892606259	NA	NA	NA	NA	NA	NA	NA	NA	NA	NA	NA	NA
**Exo5**	1.06762099021734	0.0144366799313542	1.11823045129075	0.0342739763398484	1.14685926107042	0.0280504630361148	1.08733700562042	0.0402944507931485	1.1285194984535	0.0613913806307561	1.18631004297504	0.0542487779755861	NA	NA	NA	NA	NA	NA
**Itga5**	-1.08182563365997	0.0124812098783924	-1.02046281280891	0.0451458514028226	-1.07901685157904	0.0254545357345188	-0.359436935030603	0.588336184493792	-0.313795383161954	0.63180503055254	-0.447490068995593	0.419523985083866	-0.723384561401498	0.165554751980749	-0.656594776807248	0.227669982479008	-0.789368518523739	0.100214042476384
**Prf1**	-1.42175268	0.0153746754990301	-1.36405520	0.0322033616028379	-1.36826364	0.0295776060870866	-0.460702597488	0.618168353888822	-0.40711487013	0.626594324811275	-0.49477812712	0.502286991377018	-0.30169698730798	0.743824673612044	-0.2334664705174	0.78068017416499	-0.4764085340168	0.43861674290421

	34755		287504		220719		414		8614		3497		5		48		27	
**Icos**	-1.68783013740168	0.0136206418558666	-1.61782201231217	0.0313606476995839	-1.59750019343872	0.03689752083363	-0.421255171212721	0.710130941070315	-0.358966195581525	0.722395255100507	-0.831949927656923	0.329962727252541	-1.17260348966128	0.16128070708991	-1.09932023088001	0.191847287779818	-1.30458494071471	0.0953439939561535
**Prr7**	-1.1093589414118	0.0101301436765776	-1.04430895353731	0.0344327732043391	-1.09243723199201	0.0487335579574125	-0.0756643612845394	0.925562750340058	-0.026994711262335	0.975361411293156	-0.234916042839242	0.727991782860839	-0.0903084697934426	0.901920073427449	-0.0192693849213065	0.979234500104492	-0.00482994951162833	0.993267223565753
**Enpp5**	-1.32742633307028	0.0163218550143478	-1.2629595058035	0.0479341337686174	-1.41559675908613	0.0224966718400617	-0.25114725849666	0.787850468477945	-0.207542885842709	0.811428393780193	-0.405283731090492	0.54701770770825	-1.27771002305125	0.0393152631777537	-1.21165417388011	0.0796546408632799	-1.3845174235155	0.0366714758516166
**Amer1**	1.06151628993767	0.0212740233316432	1.11361874680973	0.045360325275453	1.16859026922645	0.0189990314344763	NA	NA	NA	NA	NA	NA	0.978901338812726	0.0617370463995623	1.0376961806738	0.0798967804542738	1.07796498231565	0.0435912063171557
**Ptgs2**	-2.90533885174013	0.0402154103068289	-2.84406133088126	0.0367906740787724	-3.83684485753259	0.0129026062518667	NA	NA	NA	NA	NA	NA	NA	NA	NA	NA	NA	NA
**Ly6a**	-1.68195444427542	0.0104291663266006	-1.62129816277912	0.0439684819121301	-1.71895950023764	0.0441237036003036	-0.119760098739453	0.922442601334878	-0.0850023390471951	0.949142593984235	-0.733696135120525	0.413274459150737	-0.80970284843921	0.347075844491873	-0.746289973333848	0.416696305273428	-1.27057437173517	0.124863896264888
**Sestd1**	-1.15539505369917	0.0145774513013914	-1.09560865054922	0.0472453001172216	-1.20856782324109	0.0295083772709541	0.192846169285828	0.811413252540274	0.232132834569053	0.749634930614835	0.109133864646304	0.871874164315238	NA	NA	NA	NA	NA	NA
**Spry1**	-1.2928558778249	0.017688106700613	-1.23268687479686	0.0487777824206263	-1.37672542514498	0.0270248156013024	NA	NA	NA	NA	NA	NA	NA	NA	NA	NA	NA	NA
**Art4**	1.4076980704988	0.020163763760051	1.45364790872017	0.0491775172269051	1.86885766396376	0.0242882591500052	NA	NA	NA	NA	NA	NA	NA	NA	NA	NA	NA	NA
**Klri1**	-1.08367351621122	0.0125751355691723	-1.02235818340972	0.0451458514028226	-1.12236287983338	0.0459525494742945	0.293826998457864	0.672320253621593	0.34405535838493	0.604410373324731	0.276357210454967	0.656295800615398	0.29880613434632	0.640165020696194	0.36359063629621	0.557023304260903	0.26078349756664	0.619561958099866
**Cbarp**	-1.12231906005071	0.0159394129308369	-1.05564545815893	0.0491775172269051	-1.15795104002035	0.0340989764296978	-0.396087141173603	0.576507541123194	-0.354181794091808	0.614337017931677	-0.690395012222954	0.262859802127827	-0.419009557944423	0.518775433898633	-0.348947933217124	0.587661888156135	-0.410756803161751	0.441118020808732
**Hspbap1**	1.13105641155894	0.0227292098332731	1.18311318765769	0.0491289934642974	1.44287644396119	0.0272237199468097	1.40604081534069	0.0148503308380268	1.44086012877977	0.0402707861633759	1.65200361276705	0.0306283912710493	1.14047931734968	0.0391337831500554	1.19889174370342	0.0664853502813999	1.44351839038256	0.0372476882487234
**Gzmb**	-1.62299949783965	0.0304176243653113	-1.56637030360046	0.0498508151331209	-1.63226584368099	0.0229073157539047	-0.677475238955928	0.546661571526239	-0.633390480008277	0.539904512745289	-0.660570718275423	0.391735692173278	-0.58854983818285	0.5822678006924	-0.517269732343975	0.600564610774447	-1.04735643587297	0.134352547736943
**Acrbp**	1.26450615383981	0.0182327707243806	1.32668650951002	0.0417448201952228	1.36235879677942	0.0469106728192579	NA	NA	NA	NA	NA	NA	1.9610034216933	0.000362759117875429	2.02443290947962	0.0096784764327275	2.09823630178839	0.00624828236291838
**Cmpk2**	1.09183947872777	0.0249546495021609	1.1480605018323	0.0457128594761753	1.12709528023443	0.0400844601312282	1.1547642241706	0.0487663313899451	1.18436934566762	0.0762718454907668	0.850891388639925	0.193940966514316	0.740242147299296	0.212976880468631	0.800622232474723	0.205569608053434	0.677170920484179	0.27004433472911
**ENSMUSG00000106617**	1.06218312802312	0.0249979377717142	1.11908695988316	0.0474955404900317	1.13502932137008	0.0394800073740702	NA	NA	NA	NA	NA	NA	1.57312378903559	0.00123551264922115	1.63475095324653	0.0119441286366258	1.64399856392956	0.0079537428751854
**Adcy6**	-1.25441377739555	0.028655777192953	-1.19533351177071	0.0441015198023257	-1.22072245991416	0.0491631894006436	0.525178061857024	0.54088829860455	0.558059988143192	0.444801466910194	0.365436904473209	0.583683456477362	0.0386629655213692	0.972419063408304	0.0971755652516252	0.911178041086946	-0.161253660930126	0.812590385367088
**Crem**	-1.74529753933259	0.0404709475942242	-1.67520978989959	0.0459093723497499	-1.55489341244967	0.0385621734130529	-1.78613306752183	0.0884873133275896	-1.69918154049607	0.104006855653442	-2.39953723337691	0.0799460159576357	-1.26604341667859	0.222365198355334	-1.19179037484735	0.20702164179346	-1.08702662587215	0.166314081544868
**Stk17b**	-0.869872648790632	6.15111668880383e-09	-0.811971789415442	0.000302654145897327	-0.876082262443167	0.000157588183718101	-1.4201905576491	7.79045368075897e-17	-1.37471457922875	6.27406623717596e-05	-1.42652018622512	3.07470296277028e-05	-0.773854849115493	7.5499047792126e-06	-0.707361621329095	0.00412848850360005	-0.787770584869047	0.00230734763312951
**Pde4b**	-0.532648999440891	0.0012741443088203	-0.477652476081334	0.0168791834926725	-0.529622645560476	0.00785948118789451	-1.17127067800002	3.10431683335249e-11	-1.12777547788342	0.000286004448199555	-1.15943271140818	0.000252400878849504	-0.449056828392765	0.0213174558737766	-0.384641686984364	0.0850614366095862	-0.46089377000928	0.0346089018000659
**Mon1b**	0.669665348060673	2.90584772970752e-05	0.728620011123244	0.00121866628697694	0.683397299114223	0.00188675418150331	1.07968236116015	1.28517533006239e-09	1.12292408668814	0.000277821032242069	1.08477318220355	0.000291663258763326	0.485657318379996	0.011711893526158	0.549862213081863	0.0197524052936041	0.509777253888305	0.0275343229995296
**Atad2**	0.605231580514788	0.00036423935159829	0.660878163568374	0.00265063091090716	0.610605080562985	0.00603080504907398	1.0670997459339	1.10628955719247e-08	1.10994226873765	0.000286004448199555	1.03082269597459	0.000825016567990643	0.525583596628291	0.00816334343889054	0.589957534368639	0.0144503358860383	0.530944806008051	0.0300587767125882
**Ncbp2**	0.54322937878539	0.0239799141787167	0.601955849291955	0.0287816981477375	0.535352868422925	0.0568312876540574	1.35339343337721	2.52307802170532e-08	1.39658997655137	0.000508270647533525	1.3298121194504	0.000588206684426498	0.435782402105869	0.125726963699574	0.499331476152763	0.102875276500257	0.446365571660542	0.147283976007627
**Luc7l3**	0.87414371971399	9.85277660238554e-06	0.932884453206027	0.00168950086154073	0.861707938542958	0.00203108653263051	1.27175196571441	1.91138535998984e-08	1.31392270365891	0.000760788030789109	1.26648067634805	0.000801293739156616	0.745838321161425	0.00126656318686906	0.808339548151896	0.0118233423700172	0.765556541108247	0.0125961148569437
**Cirbp**	-0.886479872123734	0.000652745893061344	-0.823931888813414	0.00926688050480075	-0.861723909927923	0.00820308202532589	-1.58436541992642	2.82230721058562e-08	-1.53688175926502	0.00117238426905499	-1.59925046550572	0.00188400605432494	-0.723722914750664	0.0173194241482942	-0.651878609694901	0.0641997538632149	-0.689022276279416	0.0453293859480686
**Scd1**	-0.0675521166777352	0.845344372410808	-0.00949885170460931	0.977976156949205	-0.0499601924018965	0.878202472623	-1.27434678652287	1.4196825398052e-07	-1.22799290009515	0.00248559870310276	-1.25725507741042	0.00153755086762597	-0.687425699153878	0.0062378481308418	-0.622253244197194	0.0506839557627486	-0.730517619752736	0.0224872667698344
**Chordc1**	0.521886826795038	0.0983547386226131	0.57947613332266	0.0968175637598388	0.524024882301184	0.15477121934595	1.50501290954455	1.23838436849145e-06	1.54235151361788	0.00202244079079011	1.39544387556162	0.00352732904278951	0.955850845734428	0.00229439746565749	1.02066195596228	0.0149087264499678	0.889169518415851	0.0315143172746719
**Trim65**	0.882123609380275	0.00074370475797819	0.941495720449987	0.00476342716628252	0.891293975692998	0.0126449460745021	1.38765955261811	1.79919020931934e-06	1.42945516006845	0.00130869316300837	1.23253704141526	0.0058568019021961	1.11655941640246	8.68793115898926e-05	1.18255408095497	0.00401576114285457	1.12551001733522	0.00700658075677624
**Trmt1l**	0.82097668908591	6.2323169271621e-05	0.878342220471976	0.00167641585670076	0.822472331492507	0.00286634924444731	1.07204683064639	6.86973599989885e-06	1.1131719312091	0.00179843306965406	1.04368255738427	0.00274888839778775	0.833509807894684	0.000312652118063915	0.895876101504409	0.0054314062240811	0.861106419107209	0.00624828236291838
**Smad7**	-1.05583559262778	0.00108806235015916	-0.997201533515772	0.0238482009175634	-1.14896595023184	0.00441200632263559	-1.71555574014904	2.00653888972795e-06	-1.67189923043773	0.00751286362845955	-1.81751837871807	0.00282648745730102	-0.84071323892831	0.0279844979987966	-0.772750375452895	0.117539781155233	-0.907846187967119	0.0340416027897206
**Stip1**	0.193748425541423	0.539973520016489	0.251879625239718	0.406518030089459	0.194209212580082	0.52980622476968	1.17838402355723	1.15199931108026e-05	1.22123823631872	0.00296831691924805	1.14779872032336	0.00352732904278951	0.545777388210704	0.0594766999536986	0.612434562742547	0.0707463898447901	0.472696786230462	0.138235440855142
**Hspa4l**	0.506614977412274	0.254064471585802	0.562530794234175	0.232617103961775	0.543751907125124	0.309561620237649	1.83979490534456	7.51766436624151e-06	1.87074418435303	0.00391388345616951	1.59106597229481	0.0106959726775143	1.05171690837028	0.0128943313625831	1.11470684934804	0.0403957798280408	1.00581116657328	0.0729286625340911
**Slc31a1**	-0.221015095422386	0.454347199707137	-0.160696736665843	0.570168930478978	-0.183511582625924	0.506072757253491	-1.15715374001961	1.13038665528975e-06	-1.10966442234169	0.00376140787381638	-1.10629229507508	0.0105276090969099	-0.578287927616756	0.0323718460818784	-0.510011964927646	0.0927351104971718	-0.533318229976824	0.0846576917203684
**Tsc22d3**	-0.863192649485638	0.0756853146712356	-0.802833768586425	0.103362295238981	-1.01150484765674	0.0256540088562016	-2.06708849666925	3.86148877147942e-05	-2.01690946331205	0.00717976638997489	-2.07196502305247	0.00239734273287587	-1.04616814129813	0.0477443176444795	-0.979076659213855	0.0898328978979219	-1.05978265788712	0.0382734925328309

**Hsp90aa1**	-0.0227346187225326	0.970465920787521	0.036592958064703	0.935215191867631	-0.0198337645357844	0.964230106473556	1.47127155469368	0.000200381847747944	1.51482534479722	0.00292728930850382	1.39236662678011	0.00289476305165842	0.448615148889131	0.3430761424683	0.515183891488594	0.20469154929985	0.358494000877136	0.34750631928156
**Dnaja1**	0.576400949760449	0.219140858867611	0.639960387668791	0.126468772809885	0.571170372018686	0.15301339050479	1.69950884908258	0.000216249463775292	1.74514499793044	0.00335723332659133	1.64483907850008	0.00275639919608607	1.1287282665896	0.0137594730605156	1.19579156927584	0.0189383333277912	1.00865156726478	0.0284232532844265
**Ahsa2**	0.484005544457741	0.300556023574779	0.545672467271796	0.257772300032097	0.461712462738459	0.428475742840813	1.79500155801985	2.89139651767802e-05	1.83056446108486	0.00515754769079651	1.54310581717164	0.0164278604101439	1.30561316388342	0.00194444936903858	1.37106499294458	0.0166791148322282	1.21823086474099	0.0387673003057251
**Hsph1**	0.299839546182961	0.658985680684644	0.360324905569698	0.540453883023755	0.331410683538136	0.531922606282107	2.10003717256382	0.00016716271202077	2.14191312962813	0.00717976638997489	2.03617490036294	0.00274753073103107	1.22991939011122	0.0310941722737712	1.29799371992737	0.0506839557627486	1.03952006152986	0.0607965772707054
**Ndufab1**	-0.25436389176578	0.561692507283037	-0.196482614158644	0.655074091671886	-0.26987697391006	0.399008239526939	-1.53209786343455	6.88837871421897e-05	-1.48739221719659	0.00855251471412606	-1.59287159779964	0.00819541138826455	-0.167132956033747	0.755279181073782	-0.102259098027612	0.862639261595654	-0.205219730733112	0.584762524200188
**Cacybp**	0.19515013646758	0.554362425264127	0.255310839504205	0.417516190652517	0.212252453114686	0.506658857660674	1.08822178815942	0.000127658374155549	1.13371242893138	0.00635633829528364	1.07950592156962	0.00736224063655826	0.441000236984287	0.161453281062915	0.508453274276693	0.140242405970852	0.389829917250289	0.241022433676662
**Slc35c1**	0.791494331166843	0.00480758205208196	0.854386678401476	0.0131803568799367	0.811914837078535	0.0188008238307408	1.20291353269472	0.000143162394201969	1.24470271812736	0.00549333371889183	1.13435207801371	0.00954164352112136	0.529202774680429	0.125770729463671	0.595383036491048	0.108540292098998	0.563722248176269	0.131573582622053
**Mat2a**	0.881104760950295	0.000143376809307855	0.944492396826044	0.00239024067965189	0.956089017833802	0.00269701020682828	1.01634772610599	0.000237189558781814	1.06589354139471	0.00558627618344427	1.08487023491069	0.0058660007059689	0.951687504997576	0.000253947246719706	1.02111935502234	0.00519687621506871	1.03521266530587	0.00551619253660457
**Mideas**	-0.453586133502463	0.0931180842538805	-0.393689836777589	0.193267086398653	-0.403426303397139	0.169829759120529	-1.11001622803857	7.25708480362914e-05	-1.05984405403913	0.0108381358458504	-1.0665395161031	0.0103779067901569	-0.701270124779535	0.0121321864516944	-0.635168384547832	0.0706915992048993	-0.684817300644968	0.0459853503212013
**Manea**	-0.572885028870572	0.0459937192639399	-0.512393417034198	0.0958172707584294	-0.56577407943287	0.0290437833009546	-1.11527540730075	0.000323417017313881	-1.0716512551927	0.0113211906817053	-1.30477732542045	0.00245753982576456	-0.494178242275098	0.137495949253923	-0.428505834136823	0.224777992273303	-0.47879754311255	0.0880359649159379
**Zfp873**	0.748326882555261	0.0608252461850914	0.805254073886657	0.0699428574159422	0.81877357275456	0.0786715661842164	1.53092685211651	0.000220812127960876	1.57256103931835	0.00690230065309973	1.5144631692287	0.00662577343258658	NA	NA	NA	NA	NA	NA
**Gm8369**	-0.147861346356307	0.738531035051645	-0.0931270103621738	0.834131867389798	-0.167113978070841	0.588763884474378	-1.35446972129315	0.000128632788589147	-1.30433325201774	0.00989848894610223	-1.37466620035866	0.00880787020273633	-0.447276231521312	0.265733544004631	-0.382460389674196	0.371348627659849	-0.451854677010928	0.172251408005514
**Gm6377**	-1.04800833381916	0.000574724946596847	-0.983840382931851	0.0114803398743958	-1.04821200625263	0.0158452694501517	-1.42241646618034	6.53134635572878e-05	-1.37626085253931	0.00860887550536039	-1.41545795977842	0.022147652450418	0.458657021265819	0.257952433003211	0.524644478618159	0.213877398523302	0.407952857777192	0.281082980317165
**Zfp36l2**	-1.16186764132881	0.0301019705006016	-1.10303594762903	0.0717394550842909	-1.44996250122352	0.00872244795051443	-2.14371502100589	0.000237189558781814	-2.097177205748	0.0178713493572142	-2.16118672014549	0.00369015503765397	-1.65535930902015	0.00334760751430712	-1.58788153140727	0.0347539756584768	-1.65414542184941	0.0109365466330463
**Gpcpd1**	-0.667761111710725	0.0139686722048213	-0.607957000838072	0.0609241539332438	-0.607696324920402	0.05938522877683	-1.16026006656297	0.000113584542580438	-1.11646130253209	0.0121360054497932	-1.13460657502751	0.0126370923564494	-0.465246169742077	0.161287305302014	-0.397600150985443	0.285349489195283	-0.425053468495976	0.231899821256256
**Gpr68**	-0.232764072731332	0.674144775996086	-0.171688506819957	0.750790768963639	-0.209806889126248	0.729504737794014	1.62789127377925	0.000237189558781814	1.66558587639612	0.00717976638997489	1.45510939557917	0.0103133659907063	0.190269933948306	0.770331035726229	0.255292024067288	0.671886717570836	0.125855840416767	0.846869314508219
**Gypc**	-0.758044978180076	0.0135271164811334	-0.697403024681646	0.0495991635218992	-0.740677237433705	0.014749293223348	-1.27576353638753	0.000197002739898611	-1.22504939970728	0.0113934306940083	-1.27908176486307	0.0082313645523111	-0.860236873865584	0.010420706322597	-0.792934136125627	0.0543818682495159	-0.885443599055989	0.0149572986151689
**Zyg11b**	-0.25535275026988	0.430257566937525	-0.196607080209095	0.541930665163805	-0.219493974678705	0.453530468768648	-1.12396829083728	0.000140969757723496	-1.07520510302561	0.00962647731921708	-1.07907323665985	0.0143288164228532	-0.588224423387252	0.0548799036877243	-0.52278078040816	0.129430076662525	-0.551890934276235	0.0921624783223366
**ENSMUSG00000104291**	NA	NA	NA	NA	NA	NA	2.08310042125019	0.000201582729872137	2.11510561560331	0.00767764780306778	1.73754016700338	0.0196360508881233	2.45881687779177	1.19484878534418e-06	2.52350957599469	0.00262816210212776	2.52245704153762	0.00159338639086515
**Pfkm**	NA	NA	NA	NA	NA	NA	2.07223090101827	0.000382499569351684	2.09993787295257	0.0076555522182854	1.74930209092302	0.0157856621201605	NA	NA	NA	NA	NA	NA
**Cebpg**	0.890123196232135	0.00222142299180099	0.949869721537951	0.00963266638316584	0.898790123840194	0.0152467759498978	1.15510923842352	0.000621710566109268	1.1992552893297	0.00871010947035655	1.21172132929956	0.00864498434753717	0.806070654613741	0.0156679331942956	0.870203130877852	0.0329324611934557	0.87275329534175	0.0315163446665958
**Pigh**	1.03694608933561	0.0350790313360388	1.09555893512248	0.0530967975564442	1.06311910594949	0.0913377006310028	1.88761490828791	0.00031639197075557	1.92231473792574	0.00747961087325573	1.64127260356299	0.0211384659135404	NA	NA	NA	NA	NA	NA
**Dcun1d2**	0.419200262870828	0.219839311439881	0.476974439701262	0.155892544040823	0.449140600024957	0.19504108542676	1.16061740759168	0.000458567207695343	1.20139639729433	0.00727476289902964	1.01167032739636	0.0152400922929212	0.389359204359015	0.318409392026939	0.453478453165858	0.237312307191166	0.418212345860248	0.264559389489911
**Rnf138**	-0.701683149933751	0.0334895972427898	-0.640943346149469	0.0818519007475981	-0.703015948769661	0.0270661651128556	-1.27267350220828	0.000428953600064667	-1.21655740148215	0.0151068919902517	-1.29973491272579	0.010359831004158	-0.727788403374017	0.0486272809039517	-0.657788573602049	0.117926825953696	-0.706203827051667	0.046315976702677
**Abhd4**	-0.748970194251907	0.00465930231148728	-0.689645882493694	0.0309052087015357	-0.749422668481052	0.0104791893230011	-1.08406883435019	0.000399341607011865	-1.0413058839532	0.0147125546411502	-1.06269372837037	0.0128168735072873	-0.324593860267887	0.361839753916081	-0.258257541348495	0.501830891714966	-0.317633588154689	0.27787619039497
**Sesn1**	-0.531308658322481	0.356697673065113	-0.477301829631215	0.375293496480221	-0.610990455800881	0.210003957760036	-1.92712829611603	0.000390856909172313	-1.88404068830444	0.0134122367174272	-1.86374435114691	0.0178605033616744	-0.947860113445332	0.102831710290402	-0.882637543433378	0.147711045986719	-1.00742257864323	0.0743367380548611
**AI504432**	0.392074152262385	0.249833094899683	0.448678742625062	0.191319095156235	0.424129506916758	0.246520140469436	1.11970109917831	0.000694377241903065	1.15829485322985	0.0095390040022465	1.063109526625	0.0152400922929212	1.03501675345312	0.000839260256381443	1.09736387865116	0.00950050390327811	1.05091676244619	0.0095319557950411
**Tipin**	-0.532927681062273	0.087886990624278	-0.475784156221551	0.157501457403436	-0.538010070115175	0.0408756579119045	-1.11510721341175	0.000809191424353343	-1.06646736567394	0.0186291033768396	-1.15137518762045	0.0082313645523111	-1.08329108210838	0.000409189478910562	-1.01671548188529	0.0139422803820427	-1.07870931973137	0.00477072088094565
**Klhl25**	0.390046527100402	0.285252687768068	0.44972187739514	0.208711016316844	0.439716802376722	0.239947817657517	1.12628097687342	0.0013578758537915	1.17227611750951	0.0112511071704433	1.18598138476624	0.0092042702893735	0.420137819931708	0.295596397067062	0.486579670936988	0.229287258904783	0.433116450468644	0.280807918919052
**Rel**	-0.969853223127936	0.0103682047422011	-0.910907566111474	0.0257300908545281	-0.913113192059425	0.0188008238307408	-1.37535058278703	0.00158107100545493	-1.3375211433683	0.0151068919902517	-1.42519042187894	0.00819541138826455	-0.515081082508441	0.297062610726217	-0.448551301702618	0.335184006626762	-0.516143442771075	0.212642008533208
**Tgfb3**	-0.949541527570884	0.0866864526868924	-0.88157973416266	0.135294495747814	-0.792189057805903	0.15840454512926	-2.07463117204871	0.000465349698143681	-2.0072031928015	0.0140551943819728	-2.06005341982622	0.0327500112817275	NA	NA	NA	NA	NA	NA
**Dad1**	-0.561045466470075	0.0518452604285837	-0.496216052845463	0.10856090410712	-0.542095024661789	0.0822085527753118	-1.07858055110448	0.000572784153409595	-1.02465984972884	0.0142704837794521	-1.0020938318985	0.0295681258954599	-0.193139647857242	0.640371318521925	-0.122352434961639	0.770396247606537	-0.130302528703424	0.722996505874048
**Smim7**	-0.582552329195934	0.0845970007097241	-0.522078712390288	0.147512035260526	-0.547025223309237	0.110160530183047	-1.21713074269869	0.000833539616129202	-1.16950468826287	0.0168360412205771	-1.23531587037086	0.02145955652451	-0.0236279113607814	0.96990747014913	0.0436308892372516	0.937313646748563	0.00716811581266153	0.985443349015695
**Tnfsf14**	NA	NA	NA	NA	NA	NA	1.94615493112573	0.00196720502029816	1.99219899326466	0.0151068919902517	2.14987109705238	0.0103133659907063	NA	NA	NA	NA	NA	NA
**Il6st**	-	0.001880596	-	0.020483397	-	0.016955075	-	0.000730358966	-	0.01952725208	-	0.02807099041	-	0.1227811277156	-	0.223699737874	-	0.117756726024

	0.849610329963943	88030093	0.792257597316536	6918693	0.801315154902265	5334945	1.08641844987682	477825	1.03934846628926	31373	1.00854735371532	69317	0.528980572618951	14	0.463931128573029	814	0.548063971481243	48
**Mns1**	0.29375000068157	0.570553141052453	0.344351278511308	0.470575604051835	0.308270060014889	0.585197813832455	1.41679026788519	0.00166622640891895	1.45454408864327	0.0130323505756666	1.32169021486901	0.0256727317391519	0.222235812483308	0.719721585537913	0.285481895368906	0.617140512128821	0.261552988303262	0.682559756286907
**Dgke**	-0.629865799283311	0.173194010472799	-0.569702525193829	0.232142074400673	-0.565611170788219	0.182499817384117	-1.54052006720474	0.00126600603941506	-1.48715205746565	0.0209374973754576	-1.60803282827318	0.0264508898207444	-0.926037465690273	0.0519127695507452	-0.858138488334558	0.114045062936036	-0.925509848483332	0.0693329733544506
**Nanos1**	NA	NA	NA	NA	NA	NA	1.33163208597732	0.00378509164798807	1.38042273786364	0.0151035822942411	1.31071061849459	0.0134020732110321	NA	NA	NA	NA	NA	NA
**Fam219a**	-0.604110251601956	0.0769172173149395	-0.546794934300446	0.159164052574128	-0.601218894202545	0.0938788729019053	-1.20494871327106	0.00155862419871757	-1.16036843622413	0.0194273858772079	-1.17706202330489	0.0254310766249173	-0.184644851242727	0.705437052886954	-0.120740915270771	0.804654431349027	-0.164291016947465	0.676028473160385
**Ftsj1**	0.587334063134923	0.0632344041374935	0.648435542233547	0.0767519843093897	0.605233406482722	0.0960306716447006	1.02612778904924	0.00276807271396726	1.07096597815218	0.0186291033768396	1.05532645097616	0.0160023803304602	0.65503576060544	0.0564330258578968	0.721962193656508	0.0709883238946723	0.681791048198435	0.0756565808836603
**Mpc2**	-0.458825772420716	0.282844225649986	-0.401323662626482	0.372127359028347	-0.461371526006622	0.188605272808849	-1.34469298170645	0.00162869621263215	-1.2995429035517	0.0252520301853721	-1.39370930859292	0.0211384659135404	-0.36102495281728	0.475787809261908	-0.296272751800478	0.587661888156135	-0.421967423408307	0.281568075569966
**Uhrf1**	-0.124908180766347	0.782323448083234	-0.0749528054280908	0.867677309129988	-0.0745350166437136	0.869333034937426	1.04938890987523	0.00339811935176515	1.0865414602208	0.0184231577840521	1.03835619555772	0.0179113122298576	-0.483354007278667	0.214560952327402	-0.422904685521866	0.311143474865581	-0.445687358580098	0.316833654573436
**Gch1**	-0.813947467880213	0.0228283229751969	-0.750255266232884	0.0556841606752821	-0.770555137502042	0.0408968823394532	-1.2528617333907	0.00206860108700169	-1.20129513741304	0.0215659313904703	-1.22916203589199	0.0280994642513428	-1.01120473382994	0.00841809023430484	-0.940130846314032	0.0403957798280408	-1.00460678597705	0.0274838753680132
**Fam167a**	-0.380424604811126	0.366520466807726	-0.318879122982522	0.453837713246414	-0.363009184969334	0.309280891736983	-1.2761569605659	0.00167316483317468	-1.21803542748059	0.0248555678832892	-1.25846654712222	0.0303189469315461	-0.789605226134557	0.0520854216970165	-0.720992221572077	0.122107657539683	-0.874812585964698	0.0451835564518103
**ENSMUSG00000098715**	0.138846455970085	0.822718902534627	0.201585128608862	0.707220991336862	0.196592616886188	0.665902219656975	-1.53027972316999	0.00188978315885347	-1.47400155599036	0.0195272520831373	-1.53761085423702	0.0371511373082462	NA	NA	NA	NA	NA	NA
**Shmt1**	0.0779446266996292	0.881936831685641	0.132393112512562	0.771850091737061	0.15745671121129	0.724057681771261	1.07220432914656	0.0064222611888768	1.11517964085944	0.0221838095581644	1.17567542628515	0.0109909137046451	0.102279216615334	0.857511781438698	0.167178256388445	0.745910554197763	0.177120906624857	0.714035340008104
**Slc38a7**	0.0630635742995889	0.885897089074691	0.124208976227327	0.753523793157999	0.0802535639763205	0.816885442686626	-1.04238076424801	0.00191291348077731	-1.0019886969466	0.0252520301853721	-1.02967891835625	0.0348666327416725	0.250609482663032	0.529272864100179	0.318852735349557	0.423569240028153	0.268971629172829	0.407310216582687
**ENSMUSG00000070667**	-0.284004640433441	0.496950436254187	-0.223403605025611	0.598674752396322	-0.272575249247037	0.387334614613009	-1.17178444276869	0.00276807271396726	-1.12380136673845	0.029428355825665	-1.22427905773839	0.0210397086487602	-0.503946002045879	0.225320556767139	-0.436691818280757	0.339638102716568	-0.473979698806965	0.189879112327768
**Ska2**	0.706054372658908	0.149410893097429	0.763379315440071	0.139559083866949	0.62248554183981	0.2429388443712	1.44790672033064	0.00417868921426244	1.48775584799298	0.0163399127586467	1.32071713934322	0.0258148929108197	NA	NA	NA	NA	NA	NA
**Zfp658**	0.15318341824007	0.822718902534627	0.208979452898384	0.735370313817605	0.291163390721636	0.648225000535218	1.45883613723885	0.00559823886892979	1.49285326977352	0.0247574068985899	1.53650460649163	0.0152799538030447	0.684476563298502	0.226635725510249	0.744010565220239	0.212138902223272	0.818419569903387	0.152692444642682
**Ift27**	-0.0227901866970447	0.970465920787521	0.037833654724384	0.941403952625152	0.0101012714505415	0.98364397366481	-1.25384293614249	0.0027380390469013	-1.20594317041147	0.0237682650709855	-1.23333343507663	0.0383991418500548	-0.143834991865867	0.800701609930576	-0.0771081956839786	0.895634658376577	-0.135003127623655	0.768612299636788
**Gm15326**	-0.413415864523036	0.439293423017252	-0.35418020357376	0.515727516436719	-0.410890643730353	0.330725060586771	-1.51275206647997	0.0037075595831773	-1.46028584434581	0.0262998096680483	-1.57396721457984	0.0256727317391519	-0.523853598412942	0.358494712729983	-0.456244345118266	0.418817792208063	-0.578835601403231	0.220317490870698
**Adam15**	-0.95769236849539	0.0234033343385406	-0.894929412936204	0.068692684767738	-0.965640144260134	0.0279996748341016	-1.44055424528895	0.0032257295412921	-1.38845488154456	0.0277481345979805	-1.44693347842298	0.0280709904169317	-0.797724353719179	0.104867837240755	-0.727626022639712	0.174520722810797	-0.839362736966423	0.0711818169624133
**P2ry10b**	0.112154499429104	0.90046938946849	0.172798285325322	0.851561837287676	0.119095000434732	0.86955963349706	-2.03154090242117	0.00286772992853434	-1.95110973997897	0.0450789341974821	-3.38030167437443	0.0200320801214772	NA	NA	NA	NA	NA	NA
**Ehbp1**	-0.099666446335961	0.839051709753973	-0.0417804178372195	0.933101023423558	-0.0744284158634334	0.855147336951819	-1.17117525936429	0.0027380390469013	-1.12616988870303	0.0252520301853721	-1.13609294697175	0.0393667372160238	-0.13852641880963	0.793856609312339	-0.0692084847508217	0.900946769852686	-0.187119473356711	0.648314924001158
**Osm**	-0.663622701696687	0.104105786001593	-0.59743002575605	0.175240558071241	-0.683579946735409	0.102524555826949	-1.32872279990014	0.00311582646369327	-1.27874696517965	0.0248308771913783	-1.31706468845936	0.0357307046082928	0.142516098060568	0.815469412110489	0.211941609363138	0.694784707054692	0.169929784665627	0.703028015571146
**Il18r1**	0.0772682176311097	0.881936831685641	0.137210930563093	0.788047158378341	0.148576136392445	0.759249901306816	1.11056844317846	0.00464579462628729	1.16092138619618	0.0332436280638742	1.20216349024717	0.0196208295134203	0.417067525644631	0.352137086396882	0.483487710216732	0.336280921021979	0.504091643872638	0.267240758376388
**Ifih1**	0.0358633978034681	0.951826501627514	0.0869729884089295	0.86639871324135	0.0770787117597953	0.878202472623	1.11926182412364	0.00695113517098024	1.15555839449887	0.0250654623029296	1.13360654748237	0.0175125791960032	0.368244967667252	0.450003732143723	0.428668435337947	0.372111007109131	0.289694855200396	0.539380366362146
**Ikzf2**	NA	NA	NA	NA	NA	NA	1.92447444573402	0.00561290794151222	1.96491691206123	0.0247574068985899	1.83912922289997	0.0227438363476107	NA	NA	NA	NA	NA	NA
**Gbp7**	0.692119675880979	0.0451437092579062	0.749185908900599	0.0585522505787118	0.690659401489165	0.106436031169169	1.11531418573782	0.00377460684249604	1.1596089321971	0.0195272520831373	1.0473925163047	0.0448373578541601	0.949858405890778	0.00897935176083796	1.01427349898215	0.0277739395691325	0.889941715360659	0.0571723747929817
**Zcchc18**	-0.414232331853253	0.256442907136363	-0.353735700067516	0.32829169594951	-0.479360891859153	0.147269505420595	-1.07625292052082	0.00352041341078444	-1.02722772340655	0.0275338466099966	-1.03949412642069	0.0365605512048669	-0.933964314519605	0.00674947128824237	-0.866058578070833	0.0395083789807301	-0.921675666900266	0.0259306660100788
**Decr2**	-0.484104698968682	0.383312970717016	-0.432438304984621	0.45151128413957	-0.428281034663051	0.42986860306172	-1.64231950167864	0.00316628092408549	-1.59253970374251	0.0259454425404164	-1.74701108551226	0.0432288927246901	NA	NA	NA	NA	NA	NA
**Ighv14-4**	-1.53044228614283	0.0610824438187232	-1.48937622026958	0.117518669092811	-1.20638531358087	0.212288678174549	-2.73588857815537	0.00256235700408724	-2.65133067888921	0.0394021556698672	-3.62146361262779	0.0368578718169805	NA	NA	NA	NA	NA	NA
**Cmc1**	-0.829755415727767	0.0488682510442021	-0.769912496139057	0.116328057473149	-0.817364954760205	0.0344816505370019	-1.32429861651646	0.00601049208266529	-1.27642378804002	0.0392435857032424	-1.49757841923062	0.0172519986740459	-0.236503582793394	0.701546793433826	-0.169423087837163	0.789292313575664	-0.237054846465431	0.569405823419817
**Epb41l4aos**	-0.506296497428296	0.265280213579771	-0.451089031916881	0.320347392613766	-0.490535800562489	0.238764951375555	-1.34178277002378	0.00392865602288945	-1.2958688151393	0.0252520301853721	-1.28759843016264	0.0427503630324673	-0.275288667363564	0.634242726692554	-0.212206636963147	0.702250807561804	-0.269209522695977	0.561839514020647
**Stk38l**	-0.379515183410511	0.449989001156199	-0.323434622698749	0.524342056922494	-0.36363161186151	0.319847741302288	-1.34451562956614	0.00493733934238073	-1.28944295989143	0.0392827034782329	-1.49183956344073	0.0234760364074715	-0.385426650170507	0.498865620264949	-0.320581619355618	0.596887681314119	-0.384026096122745	0.366244359851648
**Jkamp**	NA	NA	NA	NA	NA	NA	1.5485355014622	0.00576753519278455	1.57967896976501	0.0225576325387899	1.31146520126512	0.0455459133197927	NA	NA	NA	NA	NA	NA
**Arl4d**	-	0.893002309	-	0.944936200	0.14660109	0.863915845	1.613296026444	0.009880829220	1.65232205084	0.03238002658	1.73804723193	0.01946307274	NA	NA	NA	NA	NA	NA

	0.113456018820175	851462	0.055158696902176	526002	9794488	326116	43	45156	435	63066	066	78607						
**Dst**	0.809152547864283	0.0725049704319367	0.861434372792448	0.095906471070483	0.854976836165894	0.115541364259452	1.3353195256655	0.00733017427237809	1.3734135423531	0.0317350109293401	1.41443266488615	0.0287084770759859	1.08870135579647	0.0218122400351366	1.14614709358107	0.0544731277564185	1.10045936409443	0.0582044941116967
**Maf**	0.125675120138836	0.800133724655499	0.188547251241461	0.665414994111285	0.134173798278336	0.788023502290588	1.05024415540376	0.00771799054379177	1.09825748079986	0.0256033060375861	1.02323802189045	0.0373943837840022	0.439500677369356	0.318379955736014	0.507897404217772	0.257995702406	0.38121785062178	0.414648326399962
**Igkv14-100**	-0.56733309600008	0.367829552240729	-0.520796059519966	0.451096388736044	-0.270083639358921	0.747377223784191	1.55911100123413	0.0117644523916641	1.59932830089841	0.0486426551176381	1.84711798097243	0.0135253051969111	NA	NA	NA	NA	NA	NA
**Mgat4a**	-0.402261939102607	0.302542232649213	-0.342856145724383	0.374441787707836	-0.416348188898357	0.247169143455934	-1.07978589706986	0.00615303617061255	-1.03510172755928	0.0354903135068567	-1.11282692732032	0.0401753652368965	-0.304016298756571	0.517159423305151	-0.239078689823247	0.616339244816556	-0.395837205125052	0.312584340853193
**Tmem29**	-0.356030081127349	0.3443352922419	-0.296241973669022	0.467786687310373	-0.345438939098689	0.318702342811894	-1.05713425418477	0.00600513303329133	-1.01503870663415	0.0360837806564407	-1.04577088949495	0.0408945447887409	-0.0166648289546389	0.978514936377786	0.0470879611857785	0.932566752543388	0.00750599015543277	0.985236586878926
**Ndufc1**	-0.140921312112801	0.754501354972561	-0.081381095506504	0.866400160596005	-0.144722810542713	0.679432974960101	-1.04595749923381	0.00619062441688026	-1.00459155553053	0.0398912419196628	-1.06038014648241	0.0366722722676089	-0.278719332438949	0.535671355082647	-0.211112305778549	0.661301572477372	-0.289207042005193	0.430300684035691
**Zfp933**	0.53505563151987	0.253392011042603	0.591992831179207	0.211519220452537	0.549957799436587	0.280860157959904	1.23981767561846	0.00872352367602304	1.27772071839396	0.0277694249269893	1.15557795051827	0.0448560231037199	NA	NA	NA	NA	NA	NA
**Arc**	-0.829893570452378	0.0350679245038609	-0.761705735549499	0.0898476881382081	-0.845550548237943	0.100341223892686	1.16957022523125	0.00845998555260691	1.20967071460011	0.0303347571762221	1.01814887773608	0.0430737562044397	0.451578095131682	0.370410516961299	0.519079467282372	0.313760774098552	0.395589806920312	0.413012021549748
**Tmem33**	0.184813304361807	0.715781747235449	0.250059141734047	0.579563275174579	0.216633085877333	0.658429109473387	1.08308637971883	0.0117262661122804	1.12807362477999	0.0301288210893993	1.03844675300024	0.0406146936680316	0.565640826529741	0.211702401548717	0.63384376543225	0.180419561974061	0.588447411564587	0.206394383865439
**Mrps6**	-0.0252983447900527	0.970465920787521	0.0339900504620112	0.950352949295629	-0.0161046165053929	0.979127982116882	1.10673093622062	0.0133405711202156	1.15550391220418	0.0301288210893993	1.09480432718928	0.0366722722676089	0.344293890229214	0.524051866419145	0.413143687407274	0.41187811338948	0.297830959423216	0.576525317412301
**Il2ra**	0.234039925058261	0.665828897177507	0.28957967573324	0.586755193991263	0.260429980595734	0.636922747417626	1.23578115963476	0.00818805872942577	1.27467704293662	0.0377832211942704	1.12858158169545	0.0490073405319887	0.229405626233503	0.710871602909537	0.292166646051992	0.642249595570079	0.0262959117104656	0.96872372713865
**Pik3ip1**	-0.614083175968492	0.101851402016878	-0.553328394449295	0.181617680252631	-0.678732649263202	0.0771040836753913	-1.10095627439415	0.00731452277853452	-1.05475613091878	0.0479495424858402	-1.11193944356374	0.0432522828301804	-0.770095164196849	0.0560746838621437	-0.700529850084799	0.140234490237987	-0.845135285460141	0.0547718221144886
**Ddit4**	-0.231341855994699	0.786837930489359	-0.175074592083969	0.812529396091605	-0.450715445079302	0.33583423570163	-1.75701614271011	0.0122567740431554	-1.69797056834739	0.0409312034393995	-1.77289891802244	0.0317624581223904	-1.46862763730004	0.0274801539598385	-1.40717509657721	0.059592842306098	-1.7143865517519	0.0124366016985227
**ENSMUSG00000103290**	NA	NA	NA	NA	NA	NA	1.64625486020841	0.0127860662374225	1.68719279243476	0.0301288210893993	1.51974934655058	0.0436696374757208	NA	NA	NA	NA	NA	NA
**Mrps16**	-0.0945438294620714	0.856547398666563	-0.0340476811744748	0.944023476819596	-0.0719847801710624	0.864663698134799	-1.06137451477999	0.010338745886897	-1.0158888833436	0.041576030809157	-1.17421522151992	0.039579210998714	-0.161372980713006	0.772692460052522	-0.0936976227311783	0.869307801619787	-0.200846803552113	0.644473339618221
**ENSMUSG00000099681**	-0.445231719518236	0.381727807817971	-0.381722619265366	0.459164377782337	-0.378874955538345	0.405456931520362	-1.30052012603815	0.0115494030253763	-1.25171746066339	0.0428924017549992	-1.44835143350234	0.0366722722676089	NA	NA	NA	NA	NA	NA
**Ptov1**	-0.339738624164466	0.442239220680783	-0.283881514617047	0.510650470697834	-0.310855898306613	0.511180790491141	1.02042340366658	0.0149272138786616	1.05774441400837	0.0359853687298146	1.00520273590689	0.0353828500220038	0.474002924992238	0.301480588396841	0.533329835139666	0.250346299395606	0.511940839967008	0.238158743213073
**Arrdc3**	-0.299932795436138	0.686008491643441	-0.247659911235118	0.69019977347422	-0.426978743080812	0.436047416110573	-1.62374682624221	0.0121433213331377	-1.58487149236892	0.0357104500411651	-1.73841654229898	0.0462926701259006	-0.419744357883026	0.599853455408498	-0.353187221705647	0.617140512128821	-0.455752514172187	0.45824301877039
**Nefh**	0.0584659332397557	0.935817204317127	0.115933702849721	0.857465866293446	0.160210074315797	0.806169421153671	1.27024568987588	0.0149272138786616	1.31143933316826	0.039014260345439	1.27413119602891	0.0368578718169805	NA	NA	NA	NA	NA	NA
**Neil3**	NA	NA	NA	NA	NA	NA	1.40704092769691	0.0231957116935739	1.43732816014175	0.0406847488467685	1.50539852937036	0.023850191822023	NA	NA	NA	NA	NA	NA
**Hk2**	-0.763998530144209	0.0406752625265617	-0.707407583192083	0.0958810770880867	-0.766362503690065	0.0448358987892124	-1.07638725758726	0.0130920378105147	-1.03160250028206	0.049541846105827	-1.08340635735451	0.0430737562044397	-0.731214897313728	0.0839618165818453	-0.665042576674045	0.149093683857237	-0.741414359698804	0.0733290090649336
**Nuak1**	NA	NA	NA	NA	NA	NA	1.3663337578631	0.0200880944575261	1.3999357946637	0.0455992394064165	1.48043102924273	0.0353828500220038	0.782365862212723	0.199642023375884	0.838964826664804	0.193596674729876	0.875239075320066	0.171376235637882
**G2e3**	1.02932554375755	0.048209606070145	1.07853556797946	0.0714874168301967	1.11739228140395	0.0623469572349746	1.32691433790782	0.0278814317010594	1.36484544297532	0.049541846105827	1.45796984786889	0.0382360785218511	NA	NA	NA	NA	NA	NA
**Alg3**	NA	NA	NA	NA	NA	NA	1.44198487483681	0.0307496206872024	1.47801454287158	0.0496525493257198	1.54291075629161	0.0456591647811323	NA	NA	NA	NA	NA	NA
**Mboat1**	NA	NA	NA	NA	NA	NA	1.38579625261395	0.0345775281503496	1.43404699137797	0.0473539259910935	1.41092563156959	0.0443960614659237	NA	NA	NA	NA	NA	NA
**Nfkbid**	-0.315680094828861	0.363889701652917	-0.255760931610467	0.494642599731458	-0.315794373037503	0.429983041910891	0.240751710560037	0.596816606150471	0.279722181176506	0.547890648013765	0.197581426826594	0.702300026833413	1.70024467847701	2.42611643647515e-09	1.76231395990927	0.00198453718894313	1.64518030690441	0.00119575545770063
**Mettl17**	0.74593941880214	0.00029945538725386	0.803960546908804	0.00337447105612909	0.756116777454041	0.00642256641743711	0.576234981566082	0.032645533076402	0.618985479842806	0.0465694050931804	0.583794899242787	0.0770542194542083	1.15649873074049	9.7590494081326e-08	1.21848013041209	0.00198453718894313	1.18891962766568	0.00119575545770063
**Fhod1**	0.762904100066788	6.52049207221243e-05	0.817905043704107	0.00189709736172447	0.781529383820345	0.00286634924444731	0.641263069682303	0.00875052715924407	0.681912447009121	0.0230242855139389	0.648362103274454	0.0401753652368965	1.02807081485313	6.83549331055715e-07	1.0903416437256	0.00198453718894313	1.0495742612401	0.00163955316281712
**Fam193b**	0.499329130975086	0.0753846609004681	0.555816804442689	0.08765643235679	0.536606259626971	0.0827310537365758	0.515400247546155	0.131429815547822	0.553336052296459	0.152124152164677	0.553819827619923	0.143831147845117	1.3151350627362	7.83196890822637e-07	1.37591453763388	0.00258396353296751	1.37260073369786	0.00159338639086515
**Slc38a2**	0.208703019143072	0.440894702384948	0.267722227717431	0.297506104671146	0.211553252985533	0.40931385202773	0.421150157802712	0.137858560452183	0.460999484850886	0.128266796734063	0.38801864986767	0.196827560446388	1.06102028360101	1.38883450633096e-06	1.12429025358937	0.00198453718894313	1.06350055915591	0.00163955316281712
**Spns1**	0.534115687252233	0.0247620523967987	0.593223210649395	0.0322962499390788	0.537200175017372	0.0644870985123671	0.697205995793885	0.0116370967172272	0.74401484967592	0.0299729734355016	0.667678924343182	0.0602444979369605	1.09400973061703	1.94909563698493e-06	1.16039896960508	0.00250337348097098	1.08244399870433	0.0028869794282333
**Malat1**	0.642106319342744	0.381374082321961	0.695340870596801	0.356028658043133	0.849274950800005	0.221553667210378	1.4079144869832	0.0656238886272543	1.44086762528727	0.111142614310651	1.61783845784906	0.0537090520338129	2.86734963056057	3.22008256335508e-06	2.92752950058492	0.00778494899030259	2.82766660279602	0.00211800845496579
**Clcf1**	0.718856979090521	0.019464511510356	0.779714206727252	0.032747147983711	0.725739929132999	0.0496494932556648	0.291580078130428	0.530850553063401	0.336438681770084	0.45911126243569	0.311003138962103	0.541700598566407	1.36577482392073	8.52503762546451e-06	1.42927926151982	0.00324340696680267	1.37433171256764	0.00296411756678496
**Aldh1b1**	-0.906960146053431	0.00704051326579639	-0.849142164170611	0.032955331833043	-0.924928508055018	0.0131765867835818	-0.218048908590286	0.693429823489684	-0.170801150764947	0.749634930614835	-0.332297924449555	0.421579712518614	-1.53518260628309	1.4709622195678e-05	-1.46484390800208	0.00401576114285457	-1.52621098309149	0.00322858031623783
**Egr2**	-0.622645537022327	0.0469302428241345	-0.559818034947021	0.100728970877856	-0.634012738022634	0.105556286964934	-0.0450700129997551	0.938027004776248	0.00101879092659958	0.998432214745866	-0.0575329154744666	0.923655475851989	1.27143828088363	2.97406601415563e-05	1.33558174226288	0.00364018834343927	1.27396783184678	0.00230734763312951

**Hes1**	-0.191729166261672	0.692676258246483	-0.129993094372826	0.785280778565534	-0.179891066972382	0.748215156448011	0.115937195532417	0.856201380941068	0.161318285266793	0.77612774787656	0.151074496543349	0.836464086859177	1.42997702270948	7.29040600250146e-05	1.49390691456381	0.00483492741892895	1.41695838109912	0.00551619253660457
**Cdca3**	-0.608477120702893	0.123846095898074	-0.554264481344598	0.187075227710474	-0.604747890632216	0.106762137642868	-0.076397694644364	0.912318686961015	-0.0333703682095924	0.962866960270064	-0.0293532930888008	0.959199845607871	-1.47533045230068	0.000115202843077483	-1.40847353715746	0.00836551905062161	-1.50661501104965	0.00626198263305467
**ENSMUSG00000097347**	NA	NA	NA	NA	NA	NA	NA	NA	NA	NA	NA	NA	2.53971712505452	0.000118377605943353	2.58959520955987	0.0105372687050837	2.77169312155077	0.00503418430344143
**Lgals4**	0.165797829748914	0.774196317454819	0.226314090128602	0.664305922754991	0.209842919646457	0.694059756121017	NA	NA	NA	NA	NA	NA	1.46797878018047	0.000317089446631751	1.53152935501594	0.00786567942676087	1.54259132637305	0.00315010258888397
**Clspn**	-0.846984662833181	0.0350679245038609	-0.794305890279943	0.0770503436848617	-0.856481206346573	0.0500369623731073	0.39765727397949	0.493150185517321	0.436656084945351	0.440663522484948	0.258723411718897	0.608327476551678	-1.52429843008836	0.000195597405602391	-1.45929876893548	0.0101341298408906	-1.68049771164058	0.00780640302934154
**Gbp9**	0.476530716543285	0.0981032158093056	0.533040173321007	0.0988953798791169	0.507164968512126	0.101732162170019	0.604387701320927	0.0718404252633266	0.64116358282764	0.096690080367187	0.61648196340113	0.105025660062379	1.01265081700103	0.000300840045774651	1.07295936773715	0.0084697324571238	1.03462945689424	0.00624828236291838
**Chkb**	0.164366252543495	0.68759762447758	0.220601703736295	0.561674757873287	0.183773883370353	0.631507951348805	0.13199443207515	0.800180376259569	0.172798493254285	0.724215454858148	0.183560646848821	0.714222465434382	1.11170467399696	0.000362759117875429	1.17304725194734	0.00862272401059574	1.15955613812754	0.0051796811861878
**Siglecg**	0.693272688189605	0.0164879655289721	0.753297329917343	0.0458421958296486	0.684798619817034	0.0610949271251872	0.401274204556291	0.317708924987297	0.444235293935004	0.32480362262954	0.393067851754323	0.394761928228528	1.14234221506131	0.000145692918705203	1.20629516819312	0.00971024106881258	1.0072576502177	0.0176255249150171
**Tmem81**	0.742434169448179	0.0125035021696867	0.801427716144783	0.0342209151606546	0.741552139046797	0.0484713334070701	NA	NA	NA	NA	NA	NA	1.08575530994128	0.000362759117875429	1.15194550941124	0.00815777762838474	1.06993164432507	0.00905106620742008
**Gabbr1**	0.194821363586811	0.654633887821603	0.24948899802849	0.549698596588456	0.247076996170036	0.531808313261855	-0.12639356551618	0.826158704817449	-0.0804111685685029	0.892324194611341	-0.0536542672192133	0.93432630440978	1.17133337681067	0.000537630434785758	1.23147966826885	0.0108339671772526	1.19530354056684	0.00551619253660457
**Gm16861**	0.682769636853984	0.19992734423232	0.745341283068307	0.177383625351528	0.727164650546474	0.247595934219075	NA	NA	NA	NA	NA	NA	1.67540778149817	0.000562772643428655	1.74085065726144	0.00827183148337801	1.64183662539016	0.00991565087221837
**Psd**	0.0851137503783782	0.855058057913781	0.144114302422797	0.72221091353121	0.0943449759710434	0.830334728793392	0.87594789107416	0.0158247041162837	0.915517354528988	0.0366541764740173	0.865783070240474	0.0434549086394805	1.05484118401131	0.00102105648293747	1.1170490159731	0.0101670721724138	1.07306190674212	0.00757323000038996
**Mapk1ip1**	0.53197514966919	0.12745861094255	0.587062412225838	0.120945671600115	0.551888700654839	0.103404501702809	-0.225123078446602	0.667191049320391	-0.180847854071475	0.73156069610134	-0.225080892290357	0.687359341695282	1.07248333632376	0.00142039173863435	1.13465846545175	0.0128191239775675	1.08862473285273	0.00665049737449153
**Ppp4r1l-ps**	0.435534944419085	0.268151259902848	0.493875883750135	0.201558341305734	0.443383325602495	0.337294231653903	0.850234001013657	0.0389828943846188	0.890372770040663	0.0550166323977712	0.772429212443966	0.142593055330873	1.19073891050316	0.000766434091493766	1.25541848725582	0.00896534565645015	1.05760514100778	0.0278647793714065
**Slc2a4rg-ps**	NA	NA	NA	NA	NA	NA	NA	NA	NA	NA	NA	NA	1.89330309106947	0.00112397790415135	1.95234580098721	0.0119281595680136	1.90141299946574	0.0148402972515475
**Ccnl2**	0.585400526539913	0.194154590950542	0.641117168019833	0.139700015954312	0.674209827179116	0.0859942506079494	0.388566410460051	0.523402693490277	0.422169619490805	0.436891363905045	0.457700044529695	0.355144389886817	1.28037509432386	0.00298977183605762	1.34012987392054	0.0131932100801433	1.36324065351517	0.00551619253660457
**Cdc20**	-0.372280738745061	0.353948472276347	-0.31595871928263	0.434269114841785	-0.306322910265503	0.418310336713988	0.489824653261938	0.284429810204798	0.532313334960287	0.255149345079589	0.584711292422611	0.146469182681546	-1.18264477362846	0.00101686872915241	-1.11761710157034	0.0182662589705256	-1.16219485737755	0.0183225071169006
**Tarbp1**	0.928175741494477	0.0215799802126243	0.982461092386245	0.0454299398059161	0.976331864853217	0.0373747537456701	NA	NA	NA	NA	NA	NA	1.28403629620786	0.00225510650924187	1.34449094518884	0.0142224639383495	1.29970664047616	0.0115854260151011
**Dennd6b**	0.825704924477926	0.0531449288105089	0.885854280176715	0.0603647911564462	0.851133054840427	0.062434332426799	0.500905151339276	0.394397698401839	0.539195013004603	0.349564491254757	0.219905454958489	0.753070110944844	1.28489097681874	0.00308876368860062	1.35156528562893	0.0154017076357948	1.30618851222058	0.0117465005322007
**Mir5125**	0.31652929083125	0.649044269606489	0.379716085191462	0.559275828383285	0.262800228000245	0.725238940695751	NA	NA	NA	NA	NA	NA	1.59176427592892	0.00346325969006819	1.65416792638964	0.0182978248156148	1.57710470778723	0.0150706590154617
**Xist**	1.07092585485128	0.0869099915138688	1.12439755028858	0.0945613666937342	1.27924141651501	0.0282972924842781	0.60082757324725	0.496362213025707	0.634727217013966	0.44135217050296	0.809657959095763	0.256632975777417	1.78046494722682	0.00533636833528199	1.83907933412285	0.0256126815112586	1.88441618791457	0.00709919463882484
**Bub1b**	-0.63410323157471	0.140214070984882	-0.58938939898535	0.203733174082524	-0.542598970318579	0.277145903345386	0.824493946865755	0.0918694907667648	0.859929382109738	0.118954089120868	0.89035270042914	0.0805810175706325	-1.3612331418489	0.00103244435338609	-1.30135011595561	0.0208975798725339	-1.26325321672556	0.0484331866933418
**Ttll3**	0.48548382409376	0.213298934909144	0.546044539353108	0.192830510099074	0.511784084414044	0.221706731819131	NA	NA	NA	NA	NA	NA	1.02956886721705	0.00450572130930647	1.09480448379231	0.0165639295909766	1.05653075067181	0.0151291935853212
**1700029J07Rik**	1.37228441401462	0.0590175000631895	1.42020039663714	0.10229616285539	2.13004247527004	0.0360257892606259	NA	NA	NA	NA	NA	NA	2.06696658147204	0.00552426039602677	2.11639960588224	0.032186965880781	2.81306198454867	0.00961486878911595
**Cdk1**	-0.985609097915389	0.0290893303534384	-0.925388625772328	0.0723906097687373	-1.06713299260039	0.0379637294831339	0.435809901225402	0.50186262635956	0.475719898947182	0.444745994781763	0.320577175671125	0.545630723880408	-1.38956730801757	0.00346432192065684	-1.32326983633807	0.0255635675264584	-1.38838283714349	0.0229477129099289
**Ankrd13d**	1.22091958452176	0.0359513849767132	1.26460073662059	0.0662270131072117	1.68677784504997	0.0200579786536246	NA	NA	NA	NA	NA	NA	1.61087034588207	0.00841809023430484	1.66147650022532	0.0306559023956361	2.07922891054427	0.00905106620742008
**4930426D05Rik**	1.17913713639828	0.0329688036115202	1.23645415802235	0.0620130599399368	1.34781453890874	0.0737966175144727	NA	NA	NA	NA	NA	NA	1.69328198091421	0.00314968303908584	1.75277184597856	0.0238327344285147	1.66597856019065	0.036217965712668
**H2-T-ps**	0.519391229429446	0.224533234895287	0.583353102215712	0.18117299723124	0.565302933536845	0.195719437987152	0.552163581310883	0.281415922563013	0.589611590926219	0.259764438221079	0.415475065087752	0.457126183660312	1.08492281723789	0.00757802213127876	1.15057578202194	0.0246231404161631	1.16730025322285	0.0150932444491428
**Neat1**	0.254590224857292	0.543679616672635	0.312026868528804	0.482273931606215	0.342726457279173	0.392943923536323	-0.0758298187654632	0.902565219152485	-0.0320513743874687	0.964576344098888	0.0445630459666628	0.947017434538753	1.00183975617678	0.00495517352846891	1.06365313693497	0.0352462733374478	1.06928084293598	0.0162345581653897
**Ogt**	1.07757804228704	0.0525028162125069	1.13020077571759	0.0642734553554378	1.27366037942696	0.0198305411871964	0.796726600305341	0.277421820676629	0.825205161099605	0.250229568905552	0.951188198281579	0.145028789367374	1.48033671228538	0.0122834774740494	1.53492920758835	0.033961175878255	1.66595512515325	0.00905106620742008
**Tnf**	0.0163674655388383	0.983589447683513	0.072732420070026	0.913126295310703	0.287142792054793	0.681642397803968	0.899770265757153	0.116040956047083	0.936270895104665	0.138006836120218	1.14088898357345	0.0938177264328587	1.27933673743215	0.00914241932743695	1.33989470592784	0.0322861475556123	1.51142509497222	0.0163080851719565
**Ss18l1**	0.619926290203739	0.152938470547135	0.680542923125069	0.151205460086021	0.597375781414484	0.188872405168772	NA	NA	NA	NA	NA	NA	1.095935691015	0.00933261680515	1.16119084395302	0.0251844492286123	1.04434580813901	0.0273552843544206
**Zfp692**	0.696275521286752	0.152938470547135	0.756862143528207	0.147013541893693	0.672716978812487	0.195499768361748	NA	NA	NA	NA	NA	NA	1.22144288077195	0.0106576788820831	1.28320948779399	0.0305912052908445	1.23665543459157	0.0234529796991359
**Pclaf**	-1.11147560955408	0.0235199192550454	-1.05685911060671	0.0620130599399368	-1.19948096881959	0.0310294503862844	0.351133391407998	0.642622838986683	0.398948247899015	0.585907643954533	0.340034564706544	0.556639573858798	-1.38275625680419	0.00886925207646027	-1.31826725605299	0.0445828808682205	-1.50909109394157	0.0237369478924398
**Prr33**	0.536937678447694	0.570907948643391	0.594636056798726	0.544647900389713	0.896694603697305	0.461597737711958	1.89944073708306	0.0304680773683743	1.92543345371365	0.0754290713638757	2.48729061712719	0.0392433653140684	2.03786948833146	0.0101308307671511	2.0881016549437	0.0480165534565386	2.6444476008894	0.0193440723056944
**Leng8**	0.531932794299377	0.294439885888481	0.587696744774503	0.188113563654548	0.644157880405251	0.116848769290611	0.361154083915919	0.594209063234774	0.394402746624783	0.486059977816772	0.401236502322063	0.450857635246317	1.0954780645298	0.0248002074305323	1.15599628806145	0.0306559023956361	1.16112129271875	0.0156586476819015
**Zfp605**	0.60999640154969	0.125826626390237	0.663946940279143	0.129359753625023	0.714068063825759	0.0981052312596996	0.0249103925681249	0.970825092437513	0.0621851311997776	0.930989253347348	0.120635208879908	0.869209450211705	1.00815487234619	0.0120106475998496	1.06740979900199	0.0370692493405793	1.0530812163209	0.0276182019669558
**B3gntl1**	0.468663483412177	0.35098034823979	0.519936065801803	0.298598039174279	0.525195297900528	0.273316743981156	NA	NA	NA	NA	NA	NA	1.09446299754818	0.0183605311567939	1.15456540283429	0.0403957798280408	1.15258920806029	0.023152815524319
**Clk1**	0.0489087438118598	0.945143991116212	0.110612552644407	0.840406497450074	-0.00610692	0.992174290187451	0.359028659241463	0.592644009304818	0.398610215769945	0.487880365108143	0.395617233969506	0.446006756714847	1.08647058292173	0.0244332889840969	1.14658685448776	0.0340819120254755	1.08419038542181	0.0210976311282157

					979128393													
**Zfp182**	0.461539395125578	0.33789138750163	0.517430704170449	0.27665206888735	0.46894276396732	0.299610114340089	NA	NA	NA	NA	NA	NA	1.06491110172728	0.0167122006450081	1.12916303565064	0.0365884834973035	1.00436322534843	0.0340416027897206
**Ppox**	0.210745519546574	0.686474669234134	0.270112340565734	0.573478562564497	0.242573708722369	0.625425425111436	-0.114658471289397	0.869205505522859	-0.0698467373946253	0.921997150822499	-0.0288222035989299	0.970738565002847	1.00732324097128	0.016703370040917	1.07083101214755	0.0394942285906176	1.0104389334059	0.033108294203474
**Mturn**	1.02476668701514	0.00821155454100489	1.08310367040349	0.0202590509052069	0.998030907917585	0.0342791293534177	NA	NA	NA	NA	NA	NA	1.01549428463064	0.01866324098194	1.08361775536222	0.035763726773699	1.04809536785651	0.0391304819542501
**Tepsin**	0.679939136652324	0.124944373065836	0.736884739546124	0.116600133365407	0.709485940431471	0.141767866430371	1.30280346418175	0.00569946051844347	1.33472316077957	0.0230242855139389	0.971393992149803	0.0826954907028555	1.04521658690106	0.0204394534849908	1.10800390038079	0.0403957798280408	1.06793756309212	0.0392920967441445
**Zfp606**	0.92552224481426	0.040304477952674	0.978715008198522	0.0622266989402224	0.949437238748919	0.0748342670066143	NA	NA	NA	NA	NA	NA	1.10802594340552	0.0220696079189329	1.16733838995809	0.0424329671078232	1.15527100610159	0.0422454051382794

Supporting Table 2. GO functional enrichment for Antigen-only comparison. Columns show FDR and annotated enriching DEGs. (Attached csv)

Supporting Table 3. GO functional enrichment for Tolerant/2nM comparison. Columns show FDR and annotated enriching DEGs. (Attached csv)

Supporting Table 4. GO functional enrichment for Desensitized/5nM comparison. Columns show FDR and annotated enriching DEGs. (Attached csv)

Supporting Table 5. Differentially expressed genes shaping each Venn diagram region.

Antigen-only	Tolerant/2nM	Desensitized/5nM	DEGs
TRUE	TRUE	TRUE	Fosl2, Nr4a3, Ier5l, Nfil3, Litaf, Vps37b, B3gnt7, Il12b, Cebpb, P2ry10, Tigit, Rab30, Klrc1, Thbs1, Ifngr1, Fam107b, H1f2, Herpud1, Plaur, Gpr171, Plcxd2, Prdm1, Shprh, Ngp, Hs3st1, Ltf, Tmppe, Parp11, Ifitm6
FALSE	TRUE	TRUE	Trim65, Dnaja1, Ahsa2, Zfp36l2, ENSMUSG00000104291, AI504432, Tipin
TRUE	FALSE	TRUE	Mapk11, Tex2, Ctsl, ENSMUSG00000105160, Bcl2l14, Rab27b, Rell1, Dntt, ENSMUSG00000108292, Cdh5, Snhg20, Rnf125, Ahr, ENSMUSG00000086825, Cd209a, Alox5ap, Lmna, Igha, Sfi1, Pdxk, Lyz1, ENSMUSG00000095200, Cd177, Anxa1, Ighj3, Lyz2, Camp, Acrbp, ENSMUSG00000106617
FALSE	FALSE	TRUE	Nfkbid, Mettl17, Fhod1, Fam193b, Slc38a2, Spns1, Malat1, Clcf1, Aldh1b1, Egr2, Hes1, Cdca3, ENSMUSG00000097347, Lgals4, Clspn, Gbp9, Chkb, Siglecg, Tmem81, Gabbr1, Gm16861, Psd, Mapk1ip1, Ppp4r1l-ps, Slc2a4rg-ps, Ccnl2, Cdc20, Tarbp1, Dennd6b, Mir5125, Xist, Bub1b, Ttll3, 1700029J07Rik, Cdk1, Ankrd13d, 4930426D05Rik, H2-T-ps, Neat1, Ogt, Tnf, Ss18l1, Zfp692, Pclaf, Prr33, Leng8, Zfp605, B3gntl1, Clk1, Zfp182, Ppox, Mturn, Tepsin, Zfp606
TRUE	TRUE	FALSE	Bhlhe40, Dusp5, Tnfaip3, Nr4a2, Rgs1, Atf3, Rgs2, Maff, Gem, Irf2bp2, Cd83, Cytip, Nr4a1, Klf6, Dusp1, Gpr132, Myc, Ccr7, Bambi, Jdp2, Rhob, Per1, Plec, 1700017B05Rik, Spry2, Frmd4b, Irs2, Trp53inp2, Csrnp1, Dhrs3, Ccrl2, Slc49a4, Ets2, Sik1, Dgcr8, Ifrd1, Grasp, Zfp7, Errfi1, Rasl11b, Zbtb10, Vegfa, Gpr65, Cxcr4, Dyrk3, Cxcr6, Cd14, Hspbap1
FALSE	TRUE	FALSE	Stk17b, Pde4b, Mon1b, Atad2, Ncbp2, Luc7l3, Cirbp, Scd1, Chordc1, Trmt1l, Smad7, Stip1, Hspa4l, Slc31a1, Tsc22d3, Hsp90aa1, Hsph1, Ndufab1, Cacybp, Slc35c1, Mat2a, Mideas, Manea, Zfp873, Gm8369, Gm6377, Gpcpd1, Gpr68, Gypc, Zyg11b, Pfkm, Cebpg, Pigh, Dcun1d2, Rnf138, Abhd4, Sesn1, Klhl25, Rel, Tgfb3, Dad1, Smim7, Tnfsf14, Il6st, Mns1, Dgke, Nanos1, Fam219a, Ftsj1, Mpc2, Uhrf1, Gch1, Fam167a, ENSMUSG00000098715, Shmt1, Slc38a7, ENSMUSG00000070667, Ska2, Zfp658, Ift27, Gm15326, Adam15, P2ry10b, Ehbp1, Osm, Il18r1, Ifih1, Ikzf2, Gbp7, Zcchc18, Decr2, Ighv14-4, Cmc1, Epb41l4aos, Stk38l, Jkamp, Arl4d, Dst, Maf, Igkv14-100, Mgat4a, Tmem29, Ndufc1, Zfp933, Arc, Tmem33, Mrps6, Il2ra, Pik3ip1, Ddit4, ENSMUSG00000103290, Mrps16, ENSMUSG00000099681, Ptov1, Arrdc3, Nefh, Neil3, Hk2, Nuak1, G2e3, Alg3, Mboat1
TRUE	FALSE	FALSE	Trib1, Runx2, Junb, Pmaip1, Ppp1r15a, Cd69, Cd274, Adora2a, Serpinb6b, Ptger4, Fos, Btg2, Dnajb9, Anxa2, Grn, Ctla2a, Ccr5, Klri2, Ptp4a1, Serpinb9, Eomes, Mxd1, Fam111a, Snx18, Jchain, Nek6, Ccl5, Atp1b1, Id2, Srgap3, Dusp10, Plscr1, Tyrobp, S100a6, Spty2d1, Gfod1, Fgl2, Gas7, Klf4, Emb, AW112010, Siglech, Hhex, Ncoa7, Samsn1, Bag3, Myh13, Sla2, Rilpl2, Hmgn3, Ptprs, Tlr1, Cox6a2,

	Chl1, Tgif1, Cd79b, Itm2c, Cd7, Mrgpre, ENSMUSG00000053541, Klrd1, Siah2, H1f0, Kit, Clu, Dstn, Cxcl16, Prr5l, Aldh1a2, Cpne2, Lad1, Socs3, Dnase1l3, Chn2, ENSMUSG00000081662, Flt3, Dnah8, Cdh1, Arpin, Il2rb, Mmp25, Plk2, 9930111J21Rik2, Fscn1, Anxa3, Cd63, Zmynd15, Ctla2b, Tmem176a, Gm4874, Jun, Nudt17, Mreg, Pdzd4, Ccl22, Glrx, Peli2, Atp2b4, Tbc1d4, Exog, Aif1, Tjp2, Tmem176b, Klre1, Sh3bp4, Ccl3, Epha2, Gm12185, F11r, Rbpms, Tlr6, Clec12a, Marcksl1, Man1c1, Gnb4, Jaml, Havcr1, Cd55os, Ttc39a, Nrgn, Gdf11, Basp1, Cadm1, Fmnl2, Kmt2a, Ptpn22, Mpeg1, Cd209d, Slfn8, Sytl3, Bst2, Chil1, Vwa5a, Atp1a3, Insl6, Ppfia4, Ccr9, Gria3, Klrk1, Il12a, ENSMUSG00000087060, Ccl4, Prnp, Chdh, Pik3r6, Sertad1, Tbc1d8, D16Ertd472e, Cd28, Grm8, Fcer1g, Phactr2, Cacnb3, Ctla4, Osbpl3, Frmd4a, Olfm1, Sulf2, Palm, Igkv8-30, Klra7, Dnajb4, Il1r2, D11Wsu47e, Hlx, Il12rb2, Fabp5, Epsti1, Tspan3, Fosb, Rasip1, 1700025G04Rik, Eno3, Ighj1, Scin, Tmem150c, Klrb1f, Palld, ENSMUSG00000107872, Heatr9, Cables1, Pxdc1, Lrp8, Klrb1a, Pls3, Laptm4b, Arhgef10l, Dmrta1, Xkrx, Il15ra, Stap2, Tnfrsf9, Pmepa1, Ighg2b, Coro2a, Cdkn1a, B3galnt1, Eno2, Zfp882, Maged1, Tnfsf12, Upb1, Sema4c, Dapk2, Strip2, Cd300lf, Itgax, Lima1, Mfge8, Ugt1a7c, Crtam, Rhoc, Arhgap28, Rtl8c, Pfkfb3, Adamts14, AI467606, Obscn, Slc9a3r2, Samd3, Gng12, Slc2a6, Zc3h12c, Myom1, Plxdc1, B3gnt8, Srxn1, Rab7b, Adam8, Slc7a11, Rab26, Id3, Trim13, H2bc8, Arhgef40, Adat1, Smim3, Stk32c, Tmbim1, Asph, Ighe, Wdcp, Insm1, Coq10b, Mycl, Avpi1, Ptprf, 5730409E04Rik, Emilin2, Zfhx3, H2-M2, Klk4, Bend4, Ildr1, Dnah2, Ifitm2, Eepd1, Ifit1bl1, Il3ra, Loxl3, Serpinb9b, Gpr157, Lgals1, P2ry14, Pdcd1lg2, Zfp324, Kifc3, Pla2g4f, Cpq, Pik3cb, 2200002D01Rik, Bbc3, Fndc5, Adam23, Cfap126, Ccr2, Cttnbp2nl, Rnd3, Zfp748, Tnfrsf4, Rasal2, Batf3, Gm12253, Adnp, Ccnj, Zbtb20, Shb, Exo5, Itga5, Prf1, Icos, Prr7, Enpp5, Amer1, Ptgs2, Ly6a, Sestd1, Spry1, Art4, Klri1, Cbarp, Gzmb, Cmpk2, Adcy6, Crem
